# Genealogical Relationships between Early Medieval and Modern Inhabitants of Piedmont

**DOI:** 10.1371/journal.pone.0116801

**Published:** 2015-01-30

**Authors:** Stefania Vai, Silvia Ghirotto, Elena Pilli, Francesca Tassi, Martina Lari, Ermanno Rizzi, Laura Matas-Lalueza, Oscar Ramirez, Carles Lalueza-Fox, Alessandro Achilli, Anna Olivieri, Antonio Torroni, Hovirag Lancioni, Caterina Giostra, Elena Bedini, Luisella Pejrani Baricco, Giuseppe Matullo, Cornelia Di Gaetano, Alberto Piazza, Krishna Veeramah, Patrick Geary, David Caramelli, Guido Barbujani

**Affiliations:** 1 Dipartimento di Biologia Evoluzionistica, Università di Firenze, 50122 Florence, Italy; 2 Dipartimento di Scienze della Vita e Biotecnologie, Università di Ferrara, 44121 Ferrara, Italy; 3 Institute for Biomedical Technologies, National Research Council, 20090 Segrate, Milan, Italy; 4 Institut de Biologia Evolutiva, CSIC-UPF, Barcelona 08003, Spain; 5 Dipartimento di Chimica, Biologia e Biotecnologie, Università di Perugia, 06123 Perugia, Italy; 6 Dipartimento di Biologia e Biotecnologie “L. Spallanzani”, Università di Pavia, 27100,Pavia,Italy; 7 Dipartimento di Storia, Archeologia e Storia dell’arte, Università Cattolica del Sacro Cuore, 20123 Milano, Italy; 8 Anthropozoologica L.B.A. s.n.c., 57123 Livorno, Italy; 9 Soprintendenza per i Beni Archeologici del Piemonte, 10122 Turin, Italy; 10 Human Genetics Foundation, 10125 Turin, Italy; 11 Department of Ecology and Evolution, State University of New York, Stony Brook, New York 11794–5245, United States of America; 12 School of Historical Studies, Institute for Advanced Study, Princeton, New Jersey 08540, United States of America; Universitat Pompeu Fabra, SPAIN

## Abstract

In the period between 400 to 800 AD, also known as the period of the Barbarian invasions, intense migration is documented in the historical record of Europe. However, little is known about the demographic impact of these historical movements, potentially ranging from negligible to substantial. As a pilot study in a broader project on Medieval Europe, we sampled 102 specimens from 5 burial sites in Northwestern Italy, archaeologically classified as belonging to Lombards or Longobards, a Germanic people ruling over a vast section of the Italian peninsula from 568 to 774. We successfully amplified and typed the mitochondrial hypervariable region I (HVR-I) of 28 individuals. Comparisons of genetic diversity with other ancient populations and haplotype networks did not suggest that these samples are heterogeneous, and hence allowed us to jointly compare them with three isolated contemporary populations, and with a modern sample of a large city, representing a control for the effects of recent immigration. We then generated by serial coalescent simulations 16 millions of genealogies, contrasting a model of genealogical continuity with one in which the contemporary samples are genealogically independent from the medieval sample. Analyses by Approximate Bayesian Computation showed that the latter model fits the data in most cases, with one exception, Trino Vercellese, in which the evidence was compatible with persistence up to the present time of genetic features observed among this early medieval population. We conclude that it is possible, in general, to detect evidence of genealogical ties between medieval and specific modern populations. However, only seldom did mitochondrial DNA data allow us to reject with confidence either model tested, which indicates that broader analyses, based on larger assemblages of samples and genetic markers, are needed to understand in detail the effects of medieval migration.

## Introduction

Few topics in European history are as controversial and disputed as the Barbarian migrations into the Roman world at the end of Antiquity. Historians have debated for centuries the magnitude, nature, and impact of the movement of populations from the borders of the Roman Empire into its heart between the fifth and seventh centuries, a movement that brought the Roman World to an end and led to the foundation of Barbarian kingdoms that are perceived as the precursors of modern nations [1].

**Figure 1:**
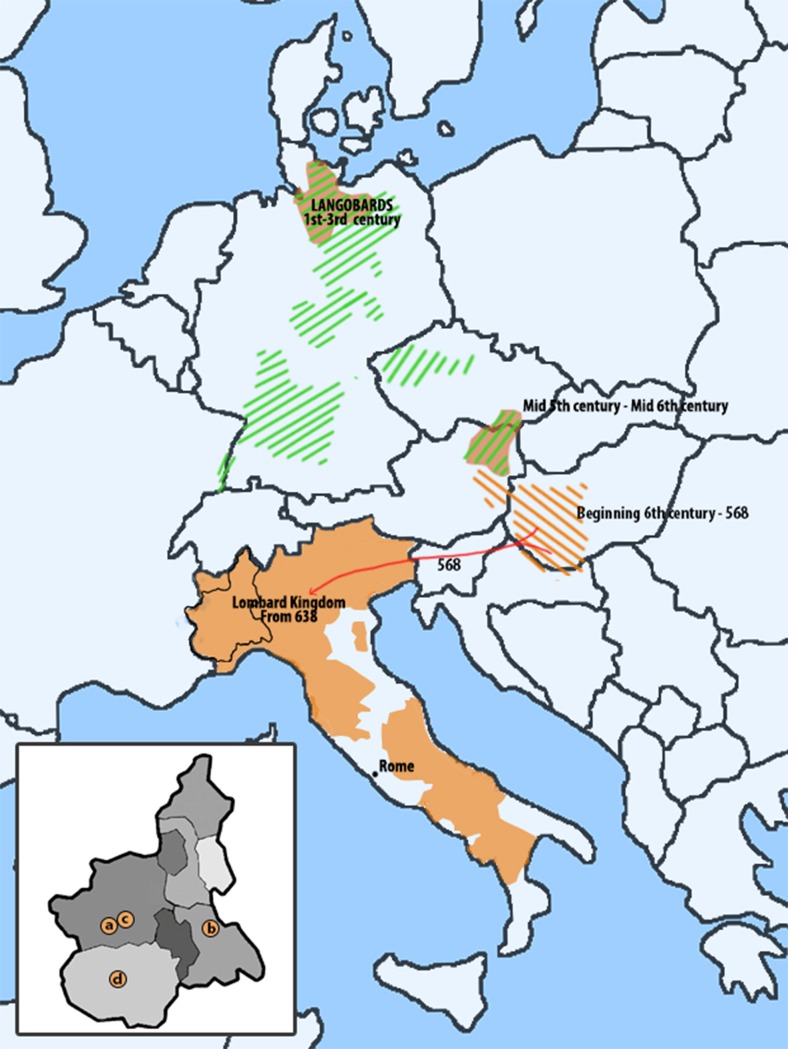
Map representing the Lombard kingdom in Italy (amber) and other areas of Europe in which cultures putatively related with Lombards have been described (shaded areas). Piedmont localities where the ancient samples were excavated in the inset: a., Rivoli; b., Mombello Monferrato; c., Collegno; d., Centallo.

One of these kingdoms was that of the Lombards. According to written sources, Lombards (sometimes known as Longobards) were a Germanic people originally settled on lower Elbe during the first century, who moved into Pannonia (a region encompassing modern day Western Hungary, Czech Republic and Eastern Austria) in the fifth and sixth centuries and then to Italy where they ruled on a large territory from 568 to 774 (Fig. 1). Archeologists commonly identify certain grave goods, settlement patterns, and burial customs as typically Lombard (though this is a matter of great debate, see [1,2,3] (but also [4]), thus suggesting a possible migration route from Northern Germany into Italy, and its approximate timing, the latter inferred from the appearance of these cultural markers in the new territories.

Traditional sources of information (i.e. written and archeological) are unable to answer questions with regard to the impact of these putative migrations upon previously-settled populations, or the nature of these migrations themselves. The earliest sources are written exclusively by Romans who lacked firsthand knowledge of the Lombards, while the most elaborate account of the Lombards, that of Paul the Deacon (ca. 720 – ca. 799), was written over two centuries after the events it claims to narrate, thus presenting a highly subjective image of these complex processes [5]. In addition, the archaeological material, albeit abundant, is generally ambiguous. Material culture is open to widely different interpretations, and in particular it is unclear whether and to what extent attributes such as grave goods and burial traditions are indicators of ethnic and social identity. Moreover, the question of whether the spread of these material markers across Europe during time is actually linked to population movements rather than to a cultural diffusion of practices remains open.

In this regard the parallel analysis of biological data (in particular genetic) from past and present populations alongside archaeological and historical information has the potential to provide us with a better understanding of human population history (for example [6,7,8,9]). Modern-day European genomes appear to be made up of a mosaic of sequence fragments with genetically distinct ancestral origins [10], indicating a complex network of interactions between past populations, perhaps resulting from a mix of short- and long-range population movements, followed by extensive local gene flow [11]. Unfortunately, as genealogical evolution is highly stochastic, it can be difficult to quantitatively associate modern genomic variation with specific demographic events, especially in the recent past, and thus few studies have attempted to address questions about human migrations in the historical era using genetic data from modern individuals.

The analysis of DNA from specimens thought to originate from a particular historical people (i.e. ancient DNA or aDNA) has the potential to significantly increase the power of such inferences. However, due to substantial technical hurdles with regard to DNA degradation and contamination, such analysis has previously been scarce, and no ancient DNA data is yet available from putative Lombards or any other Barbarian population. Fortunately, the ability to extract aDNA is improving at a rapid rate. Given these advances and the rich historical information potentially contained within aDNA, a broad project has been initiated that will attempt to extract and analyze aDNA from samples coming from several necropolises traditionally identified by cultural anthropologists as Lombard as well as neighboring necropolises traditionally seen as non-Lombard in Europe, with the first results for samples from Northwestern Italy presented here. No claim is being made concerning the ethnic identity of these early medieval populations, ethnicity being in any case a function of culture and not of genetics.

In particular, Italy is interesting because of its substantial genetic and linguistic diversity, which has been related by various authors to numerous demographic events involving populations from the pre-classic period [12,13,14,15]. Previous studies on ancient human DNA from Italy showed that both genealogical continuity and discontinuity can be recognized in different regions [6,8,9,16].

In this study we attempt to extract DNA from 102 samples from different early medieval sites in the Piedmont region spanning the sixth to the eighth centuries. In recent years, intensive archaeological surveys have been carried out focusing on the medieval contexts of this region, followed by careful studies of cultural, material, and anthropological remains, including palaeogenetic analysis, already presented as preliminary results [17,18]. We then target the hypervariable region I of mitochondrial DNA (hereafter: HVR-I) and analyze the resulting data alongside similar data from modern populations collected from the same geographic locations in order to test different demographic scenarios, ranging from one in which the medieval population comprises the majority of the current population’s ancestors, to one in which the medieval and modern populations are genetically independent.

## Materials and Methods

### DNA extraction and characterization of ancient samples

DNA extraction was attempted on 102 bone specimens (83 from Turin, 7 from Cuneo and 12 from Alessandria territories) from five early medieval contexts, archeologically defined as “Lombard”: Rivoli Corso Levi [19], Rivoli La Perosa [20], Mombello Monferrato [21,22], Collegno [19,23] and Centallo San Gervasio [24,25] (Table 1, where the GenBank accession numbers of their control-region sequences are reported). These specimens belong to Direzione Regionale per i Beni culturali del Piemonte, Soprintendenza per i Beni Archeologici del Piemonte e del Museo Egizio, Turin, which can be contacted to ask for access to the samples; the permit to analyze them was granted by a letter, protocol N MBAC-SBA-PE UFFPROT 00554324/05/2012, CI34.04.07/74.1 of May 24th, 2012, signed by the superintendent, Dr. Egle Micheletto. In Italy, no ethical committee is required to authorize studies on ancient samples. The experts who analyzed the specimens, CG, EB and LPB, are among the authors of this study, and an appropriate written informed consent was obtained from each of them. The collection and the analysis of their mtDNA was approved by the Ethics Committee for Clinical Experimentation at the University of Pavia, Board minutes of April 11th, 2013.

**Table 1:** Samples analyzed by locality and their results.

**Site (Province)**	**Age**	**N° of samples analyzed**	**N° of samples typed for HVR-I**	**Sample code**	**GenBank accession aumber**	**HVR-I motif 16024–16384 (−16000)**	**Haplogroup / Subhaplogroup**
Rivoli, Corso Levi (Turin)	VII-VIII c.	37	9	riv22	KP137645	284G 294T	H6a1b1
				riv25s1	KP137646	126C 294T	T
				riv25s2	KP137647	069T 126C	J
				riv68	KP137648	129A 356C	U4
				riv70	KP137649	CRS	H
				riv85	KP137650	CRS	H
				riv90	KP137651	051G 129C 256T	U2e1
				riv103	KP137652	129A 256T 270T 294T	U5a1b1e
				riv116	KP137653	CRS	H
Rivoli, La Perosa (Turin)	VI-VIII c.	10	4	per4	KP137667	362C	H
				per3F	KP137668	114A 192T 256T 270T 294T	U5a2a
				per9F	KP137669	311C	H2b
				per36	KP137670	CRS	H
Mombello Monferrato (Alessandria)	VI-VII c.	12	5	mom7	KP137654	CRS	H
				mom9–1	KP137655	093C 126C 153A	T2e
				mom16	KP137656	293G 311C	H24
				mom20 *	KP137657	069T 126C 231C 311C	J2a2c
				mom21–1	KP137658	069T 126C	J
Collegno (Turin)	I phase: 570–650 II phase: 650–700 III phase: VIII c.	36	8	lonI4	KP137659	145A 223T 325C 362C	I2a
				lonI41	KP137660	235G	H2a2b1
				lonI48	KP137661	293G 311C	H24
				lonI63	KP137662	126C 153A 183C 189C 294T	T2e
				lonII54	KP137663	069T 126C 153A	J
				lonIII2	KP137664	311C	H2b
				lonIII20	KP137665	CRS	H
				lonIII34 *	KP137666	256T 270T 294T	U5a1b1e
Centallo, San Gervasio (Cuneo)	VI-VII c.	7	2	cenII1	KP137671	311C	H2b
				cenII39 *	KP137672	069T 126C	J

HVR-I motifs relative to the CRS [29] are presented only for samples that gave results from at least two independent extractions. Samples marked with * were analyzed a third time in Barcelona.

All the specimens were represented by compact bone tissue; teeth were available only for a subset of the individuals, and most of them did not appear suitable for analysis because of root damages due to fractures and/or pathologies. Most of the specimens were not washed or manipulated in any way; complete information on the handling history of each specimen is available. HVS-1 sequences of all the archaeologists and the geneticists who have been in contact with the samples were determined in order to help identify possible sources of modern contamination. DNA extraction and amplification was performed in the Ancient DNA-Palaeogenetic Laboratory in Florence. This facility is exclusively dedicated to examining ancient DNA. Laboratory rooms for Pre- and Post-PCR work are strictly separated and the work was carried out while wearing clean overalls, disposable facemasks, face shields, gloves and over-shoes. Different sets of pipettes were used for DNA extraction, PCR amplification and analysis of the PCR products. All benches and rooms were routinely treated with bleach and UV-irradiated. In order to identify potential contamination, at least one extraction or amplification blank every five samples was routinely used as negative control. The specimens were cleaned by removing the external surface with a micro-drill Marathon Multi 600 with disposable tools, UV-irradiated (254nm wavelength) in a cross-linker on each side for 45 minutes and subsequently ground into a fine powder with the micro-drill. DNA was extracted from bone powder by means of a silica-based protocol [26]. Two μl of extracted DNA was used for amplification of the HVR-I region by PCR [6 modified by using a polymerase with proofreading activity]. Three primer pairs (L15995-H16132, L16107-H16261, L16247-H 16402) were used to target a subdivided 361 bp of the HVR-I via three overlapping fragments.This process was repeated for specimens reporting positive HVR-I result using a second anatomical element and a different silica-based DNA extraction protocol [27]. A third anatomical element for 3 samples was also independently examined in the Paleogenomic Lab of the Institut de Biologia Evolutiva in Barcelona. In this second laboratory, samples were extracted with a phenol-chlorophorm-based method [28] and the HVR-I was amplified using two sets of overlapping primers: L16055-H16218 and L16185-H16378.

All PCR products were cloned using TOPO TA Cloning Kit (Invitrogen) according to the manufacturer’s instructions. Screening of white recombinant colonies was accomplished by PCR [6] and agarose gel electrophoresis. After purification of these PCR products, a volume of 3 μl was cycle-sequenced using Forward M13 universal primer following the BigDye Terminator v1.1 Cycle Sequencing Kit (Applied Biosystems) supplier’s instructions. The sequence was determined using an Applied BioSystems 3100 DNA sequencer. Different clones were sequenced for each individual from whom there were two or three extractions (S1 Fig.). HVR-I sequences were then aligned and compared across clones in order to define the consensus sequence. This consensus sequence was compared to the Cambridge Reference Sequence, CRS [29] in order to define the HVR-I mutational motif. Haplogroup assignment was carried out using the software HaploGrep [30] (http://haplogrep.uibk.ac.at) with a further check based on the mitochondrial haplogroup phylogeny in Phylotree [31] (http://www.phylotree.org).

### DNA extraction and characterization of the researchers

MtDNA genotypes of all experts who handled the ancient samples were determined. After the informed consent of all donors, epithelial cells were collected from the mucosa on both sides of the oral cavity using FLOQSwabs forensic buccal swabs, DNA free (Copan flock technologies). DNA extraction as well as PCR and sequencing reaction setup involving modern samples was carried out in a laboratory that was physically separated from the laboratory where the ancient samples were analyzed. DNA was extracted using QIAmp DNA Investigator Kit (QIAGEN, Hagen, Germany) and the HVR-I was amplified by 28 cycles of PCR as reported in [32]. The entire mtDNA HVR-I region was amplified using a single primer pair named L15995-H16402. The amplification products were purified with the MinElute PCR purification Kit (Qiagen) and then sequenced directly with the same amplification primers (forward and reverse) following the BigDye Terminator v1.1 Cycle Sequencing Kit supplier’s instructions.

### DNA extraction and characterization of modern samples

The 75 samples from Trino Vercellese were collected in 1994 from individuals belonging to the Association “Partecipanza dei boschi”, whose members have been transmitting their membership from generation to generation since the Middle Age, and therefore have been settled in the same archaeologically important area for at least 20–30 generations; the 89 samples from Postua, also collected in 1994, were from individuals with Piedmontese surnames (and for this reason living in the same area for many generations) with the purpose to act as controls for the previous sample. The Authors involved in the collection were AP, GM and CDG. The samples of Trino and Postua are used only for research purposes and their individual informed consents collected by the Authors above were approved by the Ethics Committee of the University of Turin. The 58 subjects from Val di Susa were unrelated on the maternal line for at least three generations. Also in this case, for all of them, appropriate written informed consent was obtained, and the study was approved by the Ethics Committee of the University of Turin. The study of mtDNA variation of all these 222 Piedmontese samples was approved by the Ethics Committee for Clinical Experimentation at the University of Pavia, Board minutes of April 11th, 2013. The samples were anonymized prior to being accessed by the authors. Details about these samples, including the GenBank accession numbers of their control-region sequences, are available in S1 Table.

### Dataset of modern and ancient sequences for comparisons

Previously published and unpublished data were compiled to produce a dataset consisting of 7,249 sequences for 74 modern and 5 ancient populations, in order to test if the entire medieval sample could be considered a homogenous group. The dataset also comprised sequences from the Piedmont region that could be used in direct pairwise comparisons with our medieval samples in order to test for population/genealogical continuity in the region. In addition to the 222 modern individuals from Trino Vercellese, Postua and Val di Susa mentioned above, also 50 sequences from Turin, a putatively cosmopolitan population were included in the Approximate Bayesian Computation analysis in order to account for the potential effects of recent immigration [33] (S2 Table).

### Phylogenetic and population analysis

The evolutionary relationships of medieval and modern samples were investigated through median-joining networks of control-region haplotypes constructed with the Network 4.6 software (www.fluxus-engineering.com) [34] by using the reduced median algorithm (ρ = 2), followed by the median-joining algorithm (ε = 0). Nucleotide weighting (ω) was adjusted to reflect well-known phylogenetic data: i) the C-stretch between 19182 and 19193 was down weighed; ii) all samples were grouped into four major clusters corresponding to macro-haplogroups HV, UK, JT and IWX, based onto haplogroup classification reported in S1 Table. The consensus medieval sequences were compared with a dataset of 79 modern and ancient populations from Europe, North Africa, Near East and Central Asia. Variability at the intra-population level was investigated using Arlequin 3.5.1.2 [35] by calculating haplotype diversity (or heterozygosity) and mean number of pairwise differences (MPWD) (S2 Table). A matrix of pairwise φ_ST_ distances (equivalent to an F_ST_ also considering molecular distances between alleles) was estimated using the Kimura-2p model [36] using the same software, and a Multidimensional Scaling plot was obtained from that matrix using the R MASS package [37].

### Approximate Bayesian Computation analysis

We used an Approximate Bayesian Computation (ABC) framework [38] in order to investigate the genealogical and evolutionary relationships among the medieval and modern Piedmont populations. ABC utilizes simulations in order to assess the probability of data given a particular model and associated parameters (often in the absence of a tractable likelihood equation) [39]. Our ABC procedure [9] can be summarized as follows: 1 million genetic datasets are generated with the same features of the observed one (i.e. number of individuals, age of the samples, length of the sequences) by coalescent simulation for each demographic model under investigation (for a total of 16 million genealogies), drawing model parameters from the associated prior distributions. The patterns of genetic variation in the observed and simulated data are then compared via Euclidean distance using a set of summary statistics. Only the simulations generating summary statistics close to the observed ones (i.e. those associated with the shortest Euclidean distances), are then considered to estimate the posterior probabilities of the various models and parameters. For model comparison we used both the acceptance-rejection procedure (AR, [40] ) and the weighted multinomial logistic regression procedure (LR, [39]). Under the AR approach, the posterior probability of a model is obtained by considering only a certain number of “best” simulations, and then simply counting the proportion of these simulations that have been generated by each model under investigation. This method can be considered reliable only when applied to simulations (usually few) showing an excellent fit with the observed data (i.e. few hundreds, [41]). Alternatively, under the LR method, a logistic regression is fitted where the model is the categorical dependent variable and the summary statistics are the predictive variables (thus taking into account that some “best” simulations are closer to the observed data than others). The regression is local around the vector of observed summary statistics, and the probability of each model is finally evaluated in the point corresponding to the observed vector of summary statistics. The β coefficients of the regression model are estimated by maximum likelihood; the standard error of the estimates is taken as a measure of the accuracy of the method. To evaluate the stability of the so calculated models’ posterior probabilities we considered different thresholds, i.e. different number of retained simulations (100, 200, 300, 400, 500 best simulations for AR; 25000, 50000, 75000, 100000, 125000, 150000 best simulations for LR). Model parameters were estimated by a locally weighted multivariate regression [38] after a *logtan* transformation [42] of the 1,000 best-fitting simulations from a specific model. The models’ posterior probabilities were estimated using R scripts from http://code.google.com/p/popabc/source/browse/#svn%2Ftrunk%2Fscripts, modified by SG.

### Demographic Models and Summary Statistics

**Figure 2:**
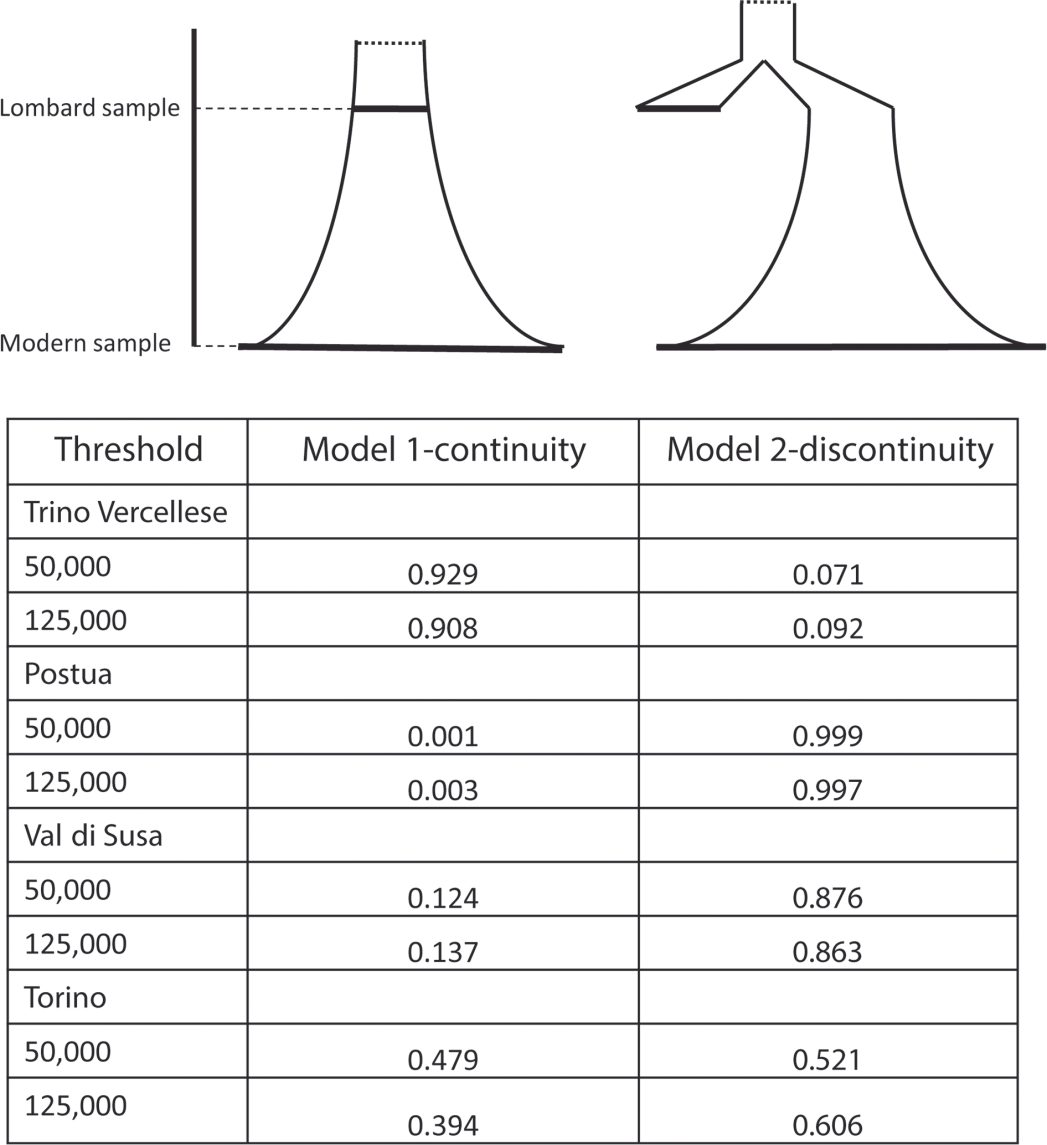
Demographic models tested and associated posterior probabilities.

We compared two main demographic models that differed with regard to the relationships between modern and ancient samples (Fig. 2). Under each model, each modern population (in turn, Trino Vercellese, Postua, Val di Susa and Turin) was independently compared with the medieval sample. In Model 1-continuity the medieval population (placed at 55 generation ago, i.e. 1375 years assuming 25 years per generation) is a direct ancestor of the modern population, and the demography follows an exponential growth that started at some point in the past (i.e. more than 55 generations ago, which is the average age of the early medieval samples). Under Model 2-discontinuity, the ancient and the modern sample descend from two different branches of a phylogeny (i.e. no genealogical relationship between them), with the medieval population not contributing to the modern gene pool, and the latter experiencing an exponential growth until the present time (Fig. 2). We also extended the comparison by including a population bottleneck mimicking the 13^th^ century plague epidemics that reduced the population size by an estimated one-third [43] size (Model 1-continuity plague and Model 2-discontinuity plague; S2 Fig.). The prior distributions were all uniform (log-uniform for the effective population sizes) and are detailed in S3 Table.

We generated 1 million simulated datasets for each model by the program BayeSSC ([44], see http://iod.ucsd.edu/simplex/ssc/BayeSSc.htm); to calculate the posterior probabilities for models and parameters we used R scripts from http://code.google.com/p/popabc/source/browse/#svn%2Ftrunk%2Fscripts, modified by SG. To summarize the data we calculated the following six statistics using Arlequin ver. 3.5.1.2 [37]: the number of haplotypes for each population, the number of private polymorphic sites per population, the mean pairwise difference and gene diversity for each population, Hudson’s *F*_ST_ [45] and a measure of allele sharing, defined as the number of haplotypes of the modern sample also present in the ancient sample, scaled by the total number of haplotypes in the latter (S4 Table).

### Type I Error and ROC analysis

We estimated the probability to reject the true null hypothesis (i.e Type I Error), by evaluating the proportion of cases in which 1,000 pseudo-observed datasets (PODs) randomly generated under each model were not correctly identified by the ABC analysis (both AR and LR procedures, 100 and 50,000 retained simulations in turn). The power of the model choice procedure was evaluated using a wide range of decision probability thresholds to identify the support for a specific model, i.e. 0.5, 0.6, 0.7, 0.8, 0.9. In addition, we calculated a receiver operating characteristic (ROC) curve, as in Bazin et al. (2010) [46] and Sousa et al. (2012) [47]. The method ranks the 2,000 posterior probabilities (1000 from each model) coming from the Type I Error analysis for one model (say, e.g., Model 1-continuity) from the highest to the lowest. For each of these posterior probabilities we know whether or not the data are generated by Model 1-continuity. At this point, the proportion of true and false positives is evaluated for decreasing thresholds, and the ROC curve is built as follows: first, we consider a posterior probability of 1.0 as the threshold for deciding whether to classify the data as coming from Model 1-continuity, having a proportion 0.0 of the simulated Model 1-continuity cases correctly classified (true positives), but also a proportion 0.0 of Model 2-discontinuity cases incorrectly classified (false positives). This represents the lower-left corner point of the ROC curve. Then we set 0.0 as the threshold, and we have a proportion of 1.0 of all the Model 1-continuity PODs classified correctly (true positives) and a proportion 1.0 of all the Model 2-discontinuity PODS incorrectly classified (false positives). This represents the point on the top right corner of the ROC curve. The remaining curve is then constructed by successively taking the posterior probabilities in the list from highest to lowest and plotting the proportion of Model 1-continuity cases that are correctly classified (true positives) and the proportion of Model 2-discontinuity cases that are incorrectly classified (false positives). The ideal curve is built when all the Model 1-continuity cases occur first in the list (i.e. higher posterior probabilities for the true model), followed by all the Model 2-discontinuity cases, in which case the area under the ROC curve (AUC) would be 1. The ROC analysis was performed with the method implemented in the ROCR R package [48].

## Results and Discussion

A total of 361 bp of the mitochondrial HVR-I was successfully sequenced in 28 samples (27% success rate; similar values have been retrieved in other comparable studies or climate conditions [6,9,49, 50] (Table 1). These 28 samples showed the same sequence in all amplicons (except for sporadic misincorporations) and were extracted and amplified at least twice from different anatomical elements (S1 Fig.). Some differences in misincorporation pattern are present between the sequences obtained from the third sample in Barcelona and the ones obtained from the first two samples analyzed in Florence. These differences could be due to the different conditions and treatments the samples were subjected to, to the different extraction methods and, above all, to the different polymerase used in the PCR reaction. There are good reasons to believe that these 28 sequences are genuine: (i) before reaching the Paleogenetic Laboratory most of the bones were not manipulated in any way, and possible handling could be tracked down for the other samples; (ii) the sequences were generated following highly stringent criteria for ancient DNA authentication (see Materials and Methods) (iii) the sequences were compared with the mtDNA motifs of the people who worked in the Palaeogenetic Laboratory (S5 Table) and no matches were found except for sequences carrying a 16311 mutation: this motif is shared between 3 medieval individuals and one of the laboratory operators. To verify this result, analysis on the second anatomical element was performed by a second operator. The result obtained from this replicate, together with the negative controls results for all the steps of both experiments suggested this haplotype as genuine; (iv) we performed each experiment at least twice, starting from different bones from the same individuals, and the result was accepted only if concordance was observed among all (either two or three) independent analyses; (v) all sequences make phylogenetic sense, i.e. do not appear to be a combination of different sequences resulting from contamination by exogenous DNA.

### Mitochondrial variation of the samples studied

**Figure 3:**
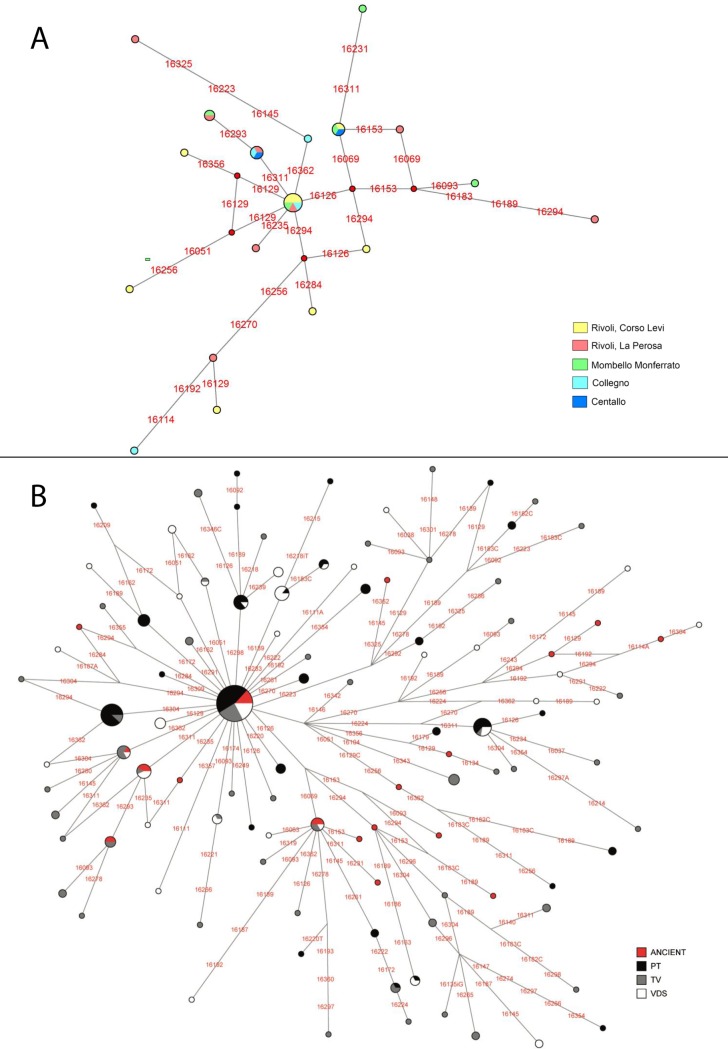
Median Joining Networks. Node sizes are proportional to haplotype frequencies. Variable positions are indicated along links that connect haplotypes. Nucleotide changes are specified only in the case of transversions. (A) Median Joining Network for the medieval sample. Different colors represent archaeological sites where the haplotype is present (see legend). Segregating sites are shown in red. (B) Median Joining Network of the ancient (colored in red) and modern samples (Trino Vercellese in grey; Postua in black and Val di Susa in white). Major haplogroups are named according to the current nomenclature.

Among the 28 medieval individuals sequenced we observed 18 distinct haplotypes with 23 segregating sites (S4A Table). Possible relationship between individuals presumed by archaeologists has been verified using genetic data in order to avoid that possible kinship could affect the haplotypic frequencies. Phylogenetic links between haplotypes and their distribution among the archeological sites are shown in a Median Joining Network (Fig. 3A). Comparing our ancient dataset to the modern haplotypes, the phylogenetic network reported in Fig. 3B reveals that five haplotypes are shared between medieval and modern samples. The 18 medieval haplotypes encompass almost the entire range of western Eurasian mtDNA macro-haplogroups (Table 1). Similarly to most modern European populations, haplogroup H is by far the most represented encompassing 50% of mtDNAs, while the remainders are members of I, J, T, U2e, U4 and U5a, all also commonly observed in Europeans [15], including the modern Piedmontese populations analyzed in this study (S1 Table).

In the Piedmont medieval sample, gene diversity (0.942) and Mean Number of Pairwise Differences (3.484) are similar to, but both lower than, the average found for the other 79 (ancient and contemporary) populations (mean 0.957 and 4.067 respectively, see S2 Table). Among other ancient populations, estimates of internal genetic variation appear close in the Etruscans (0.943; 2.966) [9] and lower in Bronze-age Sardinians (0.830; 1.390) [8], pre-Roman Iberians (gene diversity not given; 2.120) [51] and in a Medieval sample from Tuscany (0.860; 1.971) [6].

Accordingly, we see no reason to suspect that the early medieval, or “Lombard” individuals may represent a heterogeneous assemblage of people of different origins, even considering their vicinity in space and time and their archaeological affinities, and from this point on we felt justified in treating them as a single population. F_ST_ distances place Lombards in the middle of the area in which most of the populations cluster, graphically represented in S3 Fig. by a MDS plot.

We calculated two measures of genetic distance between the medieval and the modern populations from Trino Vercellese, Postua, Val di Susa and Turin, namely Hudson’s F_ST_ and the Allele Sharing (S4B Table). The lowest value of F_ST_ and the highest value of allele sharing is between medieval samples and Trino Vercellese, whereas the highest distances from the medieval samples are with Postua. Postua is the least variable sample: of the 89 individuals analyzed only 27 distinct sequences are found, and gene diversity is the lowest among the studied populations, suggesting a likely reduction in population size and thus substantial genetic drift in this population, at least along the maternal line, which may lower power if testing for continuity. The Val di Susa population shows levels of allele sharing with the Lombards similar to those observed for Trino Vercellese but a higher F_ST_, a seemingly contradictory result, possibly due to the high number of mitochondrial haplotypes, making F_ST_ a less-than-optimal descriptor of their diversity.

### Type I Error and Approximate Bayesian Computation

**Figure 4:**
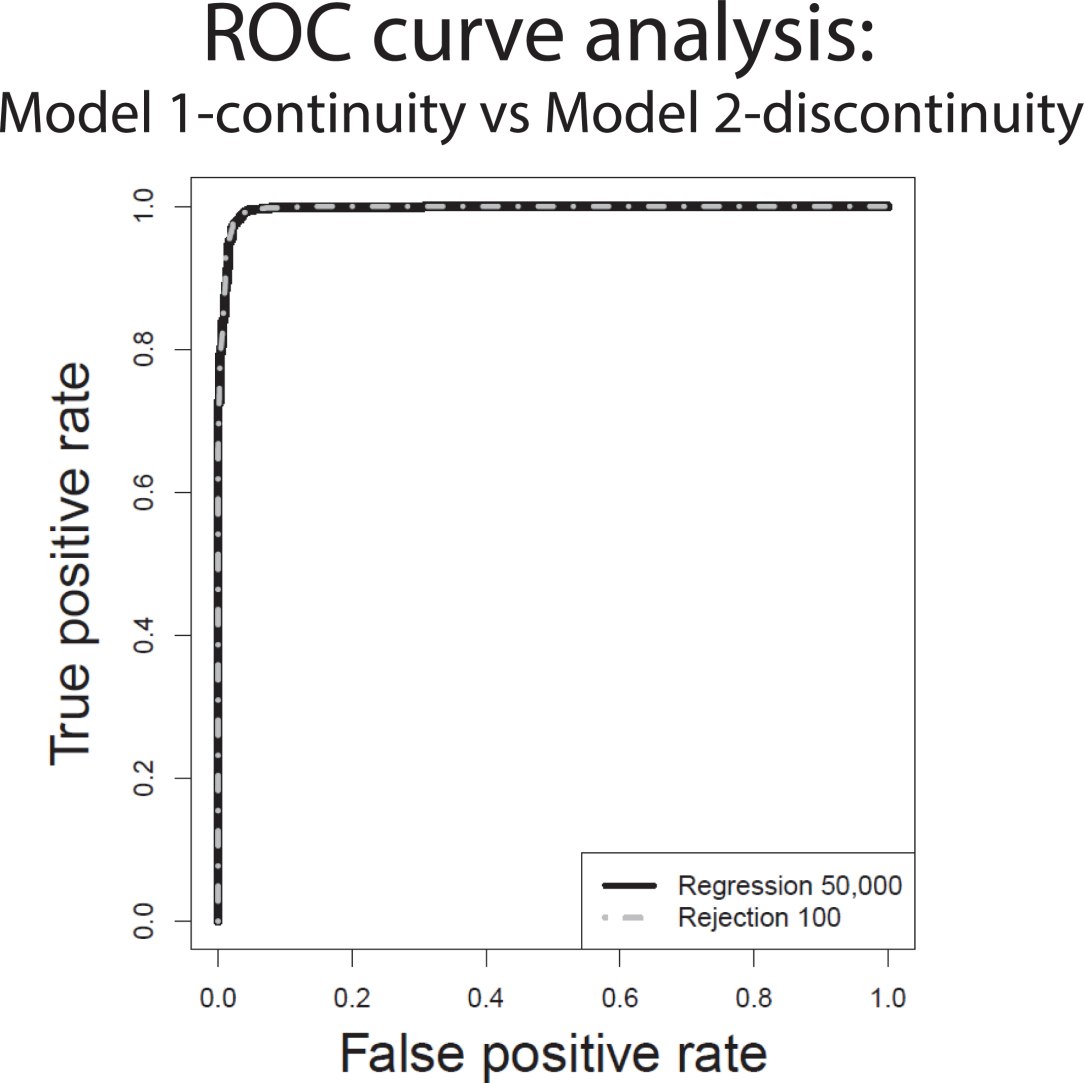
Receiver operating characteristic (ROC) curve for Model 1-continuity and Model2. Dashed line: Acceptance Rejection approach; solid line: Logistic Regression approach.

We compared Model 1- continuity and Model 2-discontinuity (detailed in Methods) to verify whether there is enough power in the data to discriminate between them using our ABC approach. S6 Table (top and bottom panels) shows the probability of identifying the true model when data are generated according to either Model 1-continuity or Model 2-discontinuity, both using the AR (100 simulations) and the LR (50,000 simulations) method. The power of the analysis was generally high; when the threshold was >0.9 (i.e. support for a model is assigned when its posterior probability is higher than 90%) the proportion of false positives was very low (0.013 at maximum). Furthermore, when the decision probability threshold was 0.5, the probability to recognize the true model was never lower than 96%. The receiver operating characteristic (ROC) curve analysis, shown in Fig. 4, confirmed the good performance of our analysis framework. For both the AR and the LR procedures, the ROC curve was close to the upper left corner of the plot, indicating that our ABC analysis efficiently identifies the model that generated the data. The capability to correctly predict the true model was also assessed comparing at the same time Model1, Model2, Model1plague, and Model2plague. Considering a decision probability threshold of 0.5, the power of our ABC procedure was generally high for each comparison, with a decrease of power (depending on the model that has to be recognized) for higher decisional thresholds (data not shown).

We then compared the fit of both models to our data, examining the medieval population alongside each of the four modern populations in separate analyses. We also repeated the analysis including a genetic bottleneck in both models (Model 1-continuity plague and Model 2-discontinuity plague) (S1 File). In all cases models without bottleneck proved to better fit the observed data than models including the bottleneck (S1 File). Fig. 2 shows the results of the comparison between Model 1-continuity and Model 2-discontinuity respectively based on the best-fitting 50,000 and 125,000 simulation experiments, under the LR procedure. We found evidence for genealogical continuity since Lombard times between our medieval sample and modern samples only when considering the population of Trino Vercellese: the posterior probability of Model 1-continuity ranged from 0.90 to 0.93. The principal component analysis (PCA) of the first 10,000 best simulations from each model (i.e. the 10,000 simulation closest to the observed dataset that are generated by each model) actually shows that the point corresponding to the observed data falls in the middle of the results obtained simulating genealogical continuity (S4A Fig.). By contrast, for all the other modern populations the best supported model was the one in which the medieval sample represents a separate branch of the genealogical tree, i.e. Model 2-discontinuity. In some cases the probability associated to Model 2-discontinuity was consistently very high (Postua, S1 File), whereas in other cases the AR and LR methods were at odds (Turin, S1 File), or returned quite different probabilities (Val di Susa, S1 File).

PCA plots (S4 Fig.) reflect the same situation. The signal for Val di Susa (S4C Fig.) is indeed quite noisy, and the observed value of Turin falls on the edge of the variation generated by Model 2-discontinuity with a slight overlap with Model 1-continuity (S4D Fig.), again emphasizing the importance of weighting the simulations with respect to their distance from the observed dataset when assigning the models’ posterior probabilities (LR procedure). Even if it is generally assumed that the logistic regression approach is more reliable than the straightforward acceptance rejection algorithm in estimating posterior probabilities [39], to strengthen these estimates we also evaluated the AR posterior probabilities for each model in a point corresponding to twice the standard error associated with the β coefficients of the fitted regression model (in both directions). For the Val di Susa sample the support was confirmed for Model 2-discontinuity, whereas for Turin the confidence in the estimated posterior probabilities increased with the number of simulations retained, and a stable support for Model 2-discontinuity was reached when considering more than 100,000 simulations (data not shown).

These inconsistences in the probabilities estimated at different thresholds are not surprising, considering the ample degree of overlap between the models we were trying to discriminate. It is probably necessary to analyze more data, both in term of loci and/or individuals, to be able to reject a model with good statistical confidence. Overall these results, along with the PCA plots of the simulated and observed data (S4 Fig.), support a model in which people of Lombard times appear to have contributed little, if at all, to the ancestry of contemporary people in three localities considered, and presumably over the whole area. There is, however, one remarkable exception, namely Trino Vercellese, where the genetic evidence suggests instead the existence of genealogical ties across more than 1,500 years. One can speculate that the existence in Trino Vercellese of an association involving essentially all families since medieval times might have contributed to maintaining a comparatively high level of genetic continuity. Much like in previous ancient DNA analyses, notably in Sardinia [8] and Tuscany [9], this study suggests that the modern population is a patchwork of groups with different genealogical histories; instances of clear-cut, long-term genetic continuity, all the way back to the Early Middle Age (as in the case for Trino Vercellese), or even much earlier (as is the case for Sardinia and Tuscany), exist and can be detected, but seem to represent less the rule than the exception.

## Conclusions

We have demonstrated that aDNA can be successfully extracted from Early Medieval European samples from Northern Italy, and provided the first data concerning the genetic variation in a human group defined by material culture as Lombard. This work also provides preliminary information about the correlation between this group and people who inhabit the same geographical area today. In particular there was evidence of genealogical continuity between this medieval population and the modern sample from Trino Vercellese, a finding that could be at least in part explained by the particular origin of the samples from Trino Vercellese. They were all members of the Comunanza dei Boschi, whose membership since medieval times is transmitted exclusively from fathers to sons. Such a patrilineal rule of inheritance, along with our observation that the mtDNA pool did not change in a significant manner, strongly argue for a genealogical continuity of the Trino population since early Medieval times. For the other modern populations analyzed (Postua, Val di Susa and Turin), the most probable model was the one in which the ancient sample belongs to a separate branch of the genealogical tree. These results were supported by different ABC model selection procedures; Type I error was very low, indicating that there is enough power in our data to distinguish among the models proposed.

Explaining why there is a better fit of models without a bottleneck is a matter of speculation at this stage. One may argue that even a dramatic reduction in population size may have limited genetic consequences if the population is large (e.g., from 10,000 to 1,000 individuals), whereas many alleles will be lost under similar conditions in a small population (e.g. from 100 to 10 individuals). Because here we considered Piedmont as a whole, it is conceivable that the black death plague epidemic, although sharply reducing the overall population size, did not have a substantial impact upon genetic diversity. In principle, many alternative scenarios, incorporating other demographic shifts, can be conceived. However, it would be pointless to model and test them when, based on the currently available data, it proved so hard just to tell apart continuity from discontinuity.

In this study we have only examined a small portion of mtDNA, a single genetic locus for which we can only make very broad generalizations (i.e. maternal continuity vs discontinuity) and test very simple models (i.e. including just a handful of parameters). To describe more complex processes, possibly estimating parameters such as the number of migrating individuals, the number of migration waves, and the presence of a genetic structure among migrants, we shall require more genetic information from the samples examined here, as well as data from other medieval (putatively Lombard and non-Lombard) populations.

To what extent these results fully reflect the demographic history of Piedmont is too early to say. The analysis of mitochondrial diversity is an admittedly limited, yet usefull, starting point for all sorts of broader genetic analyses; if the ancient samples yield little mtDNA, or if there is evidence of extensive contamination, further proceeding in the analysis would be pointless (see e.g. [52]). Therefore, we now know that it is indeed possible to extend the analysis to broader genomic regions, especially in the nucleus. Preliminary analyses are already in progress, and have the potential to identify subtler aspects of historical population changes. For that purpose, this study shows that the samples so far considered probably contain sufficient amounts of amplifiable DNA.

As for our general understanding of the demographic changes accompanying and following the collapse of the Roman Empire, it will be crucial to compare the Piedmont samples of this study with the specimens retrieved in European burial sites archaeologically associated with the Lombard culture. Only then it will be possible to understand the relative weight of migrational processes and cultural contacts in the spread of the Lombard culture. In addition, it is crucial to identify and select a set of modern populations for which extensive historical information is available. Hence, starting from this first successful description of medieval mtDNA variability, our aim will be to increase the number of samples covering the entire geographic area that has putatively been suggested to involve Lombard habitation and migration, and to investigate variability along the whole mitochondrial genome variability, the Y chromosome and at many loci across the autosomes in order to obtain a more powerful resolution of the genetic relationship during space and time regarding the migration era.

## Supporting Information

S1 Fig Amplicons of the 28 medieval sequences. DNA sequences from the clones analysed for the 28 Lombard samples. The Cambridge reference sequence with the numbering of the nucleotide positions is at the top. Nucleotides identical to the Cambridge reference sequence are indicated by dots. The clones are identified by a code composed by the necropolis identification and the burial number (as in Table 1) followed by the number of the extraction (1 and 2 in the Florence Laboratory, 3 in Barcelona).
(PDF)

S2 Fig Alternative models of the genealogical relationships among past and present populations: Model 1-continuity plague and Model 2-discontinuity plague. (PDF)

S3 Fig Multidimensional Scaling plot for φ_ST_ matrix. Populations are labelled as reported in S2 Table.
(PDF)

S4 Fig PCA of the 10,000 best simulations from Model 1-continuity and Model 2-discontinuity. A: Trino Vercellese; B: Postua; C: Val di Susa; D: Turin.
(PDF)

S1 File Models’ posterior probabilities for each comparison for different thresholds. (XLSX)

S1 Table Control-region haplotypes and haplogroup/sub-haplogroup classification of the 222 modern Piedmontese mtDNAs from Trino Vercellese, Postua and Val di Susa. (DOC)

S2 Table Additional contemporary samples considered in the analyses. Sample size (n), gene diversity and Mean Pairwise Sequence Difference (MPWD) are given.
(DOC)

S3 Table Prior distributions of the simulated models. Some distributions, marked by the asterisk, are model-specific.
(DOCX)

S4 Table Statistics summarizing intra (A) and inter (B) population genetic diversity. These values were used in the ABC analysis.
(DOCX)

S5 Table HVR-I motifs of the researchers who had been in contact with the ancient samples. (DOCX)

S6 Table Type I Error. Top panel: Acceptance-Rejection 100 simulations, Bottom panel: Logistic Regression 50,000 simulations.
(DOCX)
