# Supplementary material for: Genealogical Relationships between Early Medieval and Modern Inhabitants of Piedmont
Source: PLoS One. 2015 Jan 30;10(1):e0116801. doi: 10.1371/journal.pone.0116801 (PMC4312042; doi:10.1371/journal.pone.0116801)
Supplement: S1 Fig — DNA sequences from the clones analysed for the 28 Lombard samples. The Cambridge reference sequence with the numbering of the nucleotide positions is at the top. Nucleotides identical to the Cambridge reference sequence are indicated by dots. The clones are identified by a code composed by the necropolis identification and the burial number (as in Table 1) followed by the number of the extraction (1 and 2 in the Florence Laboratory, 3 in Barcelona). (PDF) [file pone.0116801.s001.pdf]

```
                1111111111111111111111111111111111111111111111111111111111111111111111111111111111111111111111111111111111111111111111111111111111111111111111111111111111111111111111111111111111111111111111111111111111111111111111111111111111111111111111111111111111111111111111111111111111111111111111111111111111111111111111111111111111111111111111111111111111111111
                6666666666666666666666666666666666666666666666666666666666666666666666666666666666666666666666666666666666666666666666666666666666666666666666666666666666666666666666666666666666666666666666666666666666666666666666666666666666666666666666666666666666666666666666666666666666666666666666666666666666666666666666666666666666666666666666666666666666666666
                0000000000000000000000000000000000000000000000000000000000000000000000000000111111111111111111111111111111111111111111111111111111111111111111111111111111111111111111111111222222222222222222222222222222222222222222222222222222222222222222222222222222222222222222222222333333333333333333333333333333333333333333333333333333333333333333333333333333333333
                2222223333333333444444444455555555556666666666777777777788888888889999999999000000000011111111112222222222333333333344444444445555555555666666666677777777778888888888999999999900000000001111111111222222222233333333334444444444555555555566666666667777777777888888888899999999990000000000111111111122222222223333333333444444444455555555556666666666777777777788888
                4567890123456789012345678901234567890123456789012345678901234567890123456789012345678901234567890123456789012345678901234567890123456789012345678901234567890123456789012345678901234567890123456789012345678901234567890123456789012345678901234567890123456789012345678901234567890123456789012345678901234567890123456789012345678901234567890123456789012345678901234

CRS             TTCTTTCATGGGGAAGCAGATTTGGGTACCACCCAAGTATTGACTCACCCATCAACAACCGCTATGTATTTCGTACATTACTGCCAGCCACCATGAATATTGTACGGTACCATAAATACTTGACCACCTGTAGTACATAAAAACCCAATCCACATCAAAACCCCCTCCCCATGCTTACAAGCAAGTACAGCAATCAACCCTCAACTATCACACATCAACTGCAACTCCAAAGCCACCCCTCACCCACTAGGATACCAACAAACCTACCCACCCTTAACAGTACATAGTACATAAAGCCATTTACCGTACATAGCACATTACAGTCAAATCCCTTCTCGTCCCCATGGATGACCCCCCTCAG

riv22_1         ............................................................................................................ACCATAAATACTTGACCACCTGTAG
riv22_1         ............................................................................................................
riv22_1         ............................................................................................................
riv22_1         ............................................................................................................
riv22_1         ............................................................................................................
riv22_1         ............................................................................................................
riv22_1         ............................................................................................................
riv22_1         ............................................................................................................
riv22_2         ............................................................................................................
riv22_2         ............................................................................................................
riv22_2         ............................................................................................................
riv22_2         ............................................................................................................
riv22_2         ............................................................................................................
riv22_2         ............................................................................................................
riv22_2         ............................................................................................................
riv22_2         ............................................................................................................
riv22_1                                                                    CGCTATGTATTTCGTACATTACTGC.........................................................................................................................................................CCTCACCCACTAGGATACCA
riv22_1                                                                                             .........................................................................................................................................................
riv22_1                                                                                             .........................................................................................................................................................
riv22_1                                                                                             .........................................................................................................................................................
riv22_1                                                                                             .........................................................................................................................................................
riv22_1                                                                                             .........................................................................................................................................................
riv22_1                                                                                             .........................................................................................................................................................
riv22_1                                                                                             .........................................................................................................................................................
riv22_2                                                                                             .........................................................................................................................................................
riv22_2                                                                                             .........................................................................................................................................................
riv22_2                                                                                             .........................................................................................................................................................
riv22_2                                                                                             .........................................................................................................................................................
riv22_2                                                                                             .....................A.......................................................................................................................................
riv22_2                                                                                             .........................................................................................................................................................
riv22_2                                                                                             .........................................................................................................................................................
riv22_2                                                                                             ............................................................T............................................................................................
riv22_1                                                                                                                                                                                                                  CAACTATCACACATCAACTGCAA....................................G.........T..........................................................................................
riv22_1                                                                                                                                                                                                                                         ....................................G.........T..........................................................................................
riv22_1                                                                                                                                                                                                                                         ....................................G.........T..........................................................................................
riv22_1                                                                                                                                                                                                                                         ....................................G.........T..........................................................................................
riv22_1                                                                                                                                                                                                                                         ....................................G.........T..........................................................................................
riv22_1                                                                                                                                                                                                                                         ....................................G.........T..........................................................................................
riv22_1                                                                                                                                                                                                                                         ....................................G.........T..........................................................................................
riv22_1                                                                                                                                                                                                                                         ....................................G.........T..........................................................................................
riv22_2                                                                                                                                                                                                                                         ....................................G.........T..........................................................................................
riv22_2                                                                                                                                                                                                                                         ....................................G.........T..........................................................................................
riv22_2                                                                                                                                                                                                                                         ....................................G.........T..........................................................................................
riv22_2                                                                                                                                                                                                                                         ....................................G.........T..........................................................................................
riv22_2                                                                                                                                                                                                                                         ....................................G.........T..........................................................................................
riv22_2                                                                                                                                                                                                                                         ....................................G.........T..........................................................................................
riv22_2                                                                                                                                                                                                                                         ....................................G.........T..........................................................................................
riv22_2                                                                                                                                                                                                                                         ....................................G.........T..........................................................................A...............

riv22 consensus
                TTCTTTCATGGGGAAGCAGATTTGGGTACCACCCAAGTATTGACTCACCCATCAACAACCGCTATGTATTTCGTACATTACTGCCAGCCACCATGAATATTGTACGGTACCATAAATACTTGACCACCTGTAGTACATAAAAACCCAATCCACATCAAAACCCCCTCCCCATGCTTACAAGCAAGTACAGCAATCAACCCTCAACTATCACACATCAACTGCAACTCCAAAGCCACCCCTCACCCACTAGGATACCAACAGACCTACCCATCCTTAACAGTACATAGTACATAAAGCCATTTACCGTACATAGCACATTACAGTCAAATCCCTTCTCGTCCCCATGGATGACCCCCCTCAG
```

```
                1111111111111111111111111111111111111111111111111111111111111111111111111111111111111111111111111111111111111111111111111111111111111111111111111111111111111111111111111111111111111111111111111111111111111111111111111111111111111111111111111111111111111111111111111111111111111111111111111111111111111111111111111111111111111111111111111111111111111111
                6666666666666666666666666666666666666666666666666666666666666666666666666666666666666666666666666666666666666666666666666666666666666666666666666666666666666666666666666666666666666666666666666666666666666666666666666666666666666666666666666666666666666666666666666666666666666666666666666666666666666666666666666666666666666666666666666666666666666666
                0000000000000000000000000000000000000000000000000000000000000000000000000000111111111111111111111111111111111111111111111111111111111111111111111111111111111111111111111111222222222222222222222222222222222222222222222222222222222222222222222222222222222222222222222222333333333333333333333333333333333333333333333333333333333333333333333333333333333333
                2222223333333333444444444455555555556666666666777777777788888888889999999999000000000011111111112222222222333333333344444444445555555555666666666677777777778888888888999999999900000000001111111111222222222233333333334444444444555555555566666666667777777777888888888899999999990000000000111111111122222222223333333333444444444455555555556666666666777777777788888
                4567890123456789012345678901234567890123456789012345678901234567890123456789012345678901234567890123456789012345678901234567890123456789012345678901234567890123456789012345678901234567890123456789012345678901234567890123456789012345678901234567890123456789012345678901234567890123456789012345678901234567890123456789012345678901234567890123456789012345678901234

CRS             TTCTTTCATGGGGAAGCAGATTTGGGTACCACCCAAGTATTGACTCACCCATCAACAACCGCTATGTATTTCGTACATTACTGCCAGCCACCATGAATATTGTACGGTACCATAAATACTTGACCACCTGTAGTACATAAAAACCCAATCCACATCAAAACCCCCTCCCCATGCTTACAAGCAAGTACAGCAATCAACCCTCAACTATCACACATCAACTGCAACTCCAAAGCCACCCCTCACCCACTAGGATACCAACAAACCTACCCACCCTTAACAGTACATAGTACATAAAGCCATTTACCGTACATAGCACATTACAGTCAAATCCCTTCTCGTCCCCATGGATGACCCCCCTCAG

riv25S1_1       ......................................................................................................C.....ACCATAAATACTTGACCACCTGTAG
riv25S1_1       ......................................................................................................C.....
riv25S1_1       ......................................................................................................C.....
riv25S1_1       ......................................................................................................C.....
riv25S1_1       ......................................................................................................C.....
riv25S1_1       ......................................................................................................C.....
riv25S1_1       .............................................T........................................................C.....
riv25S1_1       ......................................................................................................C.....
riv25S1_2       ......................................................................................................C.....
riv25S1_2       ......................................................................................................C.....
riv25S1_2       ......................................................................................................C.....
riv25S1_2       .............................................T........................................................C.....
riv25S1_2       ......................................................................................................C.....
riv25S1_2       ......................................................................................................C.....
riv25S1_2       ......................................................................................................C.....
riv25S1_2       ......................................................................................................C.....
riv25S1_1                                                                  CGCTATGTATTTCGTACATTACTGC..................C......................................................................................................................................CCTCACCCACTAGGATACCA
riv25S1_1                                                                                           ..................C......................................................................................................................................
riv25S1_1                                                                                           ..................C..................................................................T...................................................................
riv25S1_1                                                                                           ..................C......................................................................................................................................
riv25S1_1                                                                                           ..................C......................................................................................................................................
riv25S1_1                                                                                           ..................C......................................................................................................................................
riv25S1_1                                                                                           ..................C......................................................................................................................................
riv25S1_1                                                                                           ..................C......................................................................................................................................
riv25S1_2                                                                                           ..................C......................................................................................................................................
riv25S1_2                                                                                           ..................C......................................................................................................................................
riv25S1_2                                                                                           ..................C......................................................................................................................................
riv25S1_2                                                                                           ..................C......................................................................................................................................
riv25S1_2                                                                                           ..................C......................................................................................................................................
riv25S1_2                                                                                           ..................C......................................................................................................................................
riv25S1_2                                                                                           ..................C......................................................................................................................................
riv25S1_2                                                                                           ..................C......................................................................................................................................
riv25S1_1                                                                                                                                                                                                                  CAACTATCACACATCAACTGCAA..............................................T..........................................................................................
riv25S1_1                                                                                                                                                                                                                                         ..............................................T..........................................................................................
riv25S1_1                                                                                                                                                                                                                                         ..............................................T..........................................................................................
riv25S1_1                                                                                                                                                                                                                                         ..............................................T..........................................................................................
riv25S1_1                                                                                                                                                                                                                                         ..............................................T..........................................................................................
riv25S1_1                                                                                                                                                                                                                                         ..............................................T..........................................................................................
riv25S1_1                                                                                                                                                                                                                                         ..............................................T..........................................................................................
riv25S1_1                                                                                                                                                                                                                                         ..............................................T..........................................................................................
riv25S1_2                                                                                                                                                                                                                                         ..............................................T..........................................................................................
riv25S1_2                                                                                                                                                                                                                                         ...........................................T..T..........................................................................................
riv25S1_2                                                                                                                                                                                                                                         ..............................................T..........................................................................................
riv25S1_2                                                                                                                                                                                                                                         ..............................................T..........................................................................................
riv25S1_2                                                                                                                                                                                                                                         ..............................................T..........................................................................................
riv25S1_2                                                                                                                                                                                                                                         ..............................................T..........................................................................................
riv25S1_2                                                                                                                                                                                                                                         ..............................................T..........................................................................................
riv25S1_2                                                                                                                                                                                                                                         ..............................................T..........................................................................................

riv25S1 consensus
                TTCTTTCATGGGGAAGCAGATTTGGGTACCACCCAAGTATTGACTCACCCATCAACAACCGCTATGTATTTCGTACATTACTGCCAGCCACCATGAATATTGCACGGTACCATAAATACTTGACCACCTGTAGTACATAAAAACCCAATCCACATCAAAACCCCCTCCCCATGCTTACAAGCAAGTACAGCAATCAACCCTCAACTATCACACATCAACTGCAACTCCAAAGCCACCCCTCACCCACTAGGATACCAACAAACCTACCCATCCTTAACAGTACATAGTACATAAAGCCATTTACCGTACATAGCACATTACAGTCAAATCCCTTCTCGTCCCCATGGATGACCCCCCTCAG
```

```
                1111111111111111111111111111111111111111111111111111111111111111111111111111111111111111111111111111111111111111111111111111111111111111111111111111111111111111111111111111111111111111111111111111111111111111111111111111111111111111111111111111111111111111111111111111111111111111111111111111111111111111111111111111111111111111111111111111111111111111111
                6666666666666666666666666666666666666666666666666666666666666666666666666666666666666666666666666666666666666666666666666666666666666666666666666666666666666666666666666666666666666666666666666666666666666666666666666666666666666666666666666666666666666666666666666666666666666666666666666666666666666666666666666666666666666666666666666666666666666666666
                0000000000000000000000000000000000000000000000000000000000000000000000000000111111111111111111111111111111111111111111111111111111111111111111111111111111111111111111111111111111112222222222222222222222222222222222222222222222222222222222222222222222222222222222222222222222222222333333333333333333333333333333333333333333333333333333333333333333333333333333333
                2222223333333333444444444455555555556666666666777777777788888888889999999999000000000011111111112222222222333333333344444444445555555555666666666677777777778888888888999999999900000000001111111111222222222233333333334444444444555555555566666666667777777777888888888899999999990000000000111111111122222222223333333333444444444455555555556666666666777777777788888
                4567890123456789012345678901234567890123456789012345678901234567890123456789012345678901234567890123456789012345678901234567890123456789012345678901234567890123456789012345678901234567890123456789012345678901234567890123456789012345678901234567890123456789012345678901234567890123456789012345678901234567890123456789012345678901234567890123456789012345678901234

CRS             TTCTTTCATGGGGAAGCAGATTTGGGTACCACCCAAGTATTGACTCACCCATCAACAACCGCTATGTATTTCGTACATTACTGCCAGCCACCATGAATATTGTACGGTACCATAAATACTTGACCACCTGTAGTACATAAAAACCCAATCCACATCAAAACCCCCTCCCCATGCTTACAAGCAAGTACAGCAATCAACCCTCAACTATCACACATCAACTGCAACTCCAAAGCCACCCCTCACCCACTAGGATACCAACAAACCTACCCACCCTTAACAGTACATAGTACATAAAGCCATTTACCGTACATAGCACATTACAGTCAAATCCCTTCTCGTCCCCATGGATGACCCCCCTCAG

riv25S2_1       .............................................T........................................................C.....ACCATAAATACTTGACCACCTGTAG
riv25S2_1       .............................................T........................................................C.....
riv25S2_1       .............................................T........................................................C.....
riv25S2_1       .............................................T........................................................C.....
riv25S2_1       .............................................T........................................................C.....
riv25S2_1       .............................................T........................................................C.....
riv25S2_1       .............................................T........................................................C.....
riv25S2_1       .............................................T........................................................C.....
riv25S2_2       .............................................T........................................................C.....
riv25S2_2       .............................................T........................................................C.....
riv25S2_2       .............................................T........................................................C.....
riv25S2_2       .............................................T........................................................C.....
riv25S2_2       .............................................T........................................................C.....
riv25S2_2       .............................................T........................................................C.....
riv25S2_2       .............................................T........................................................C.....
riv25S2_2       .............................................T........................................................C.....
riv25S2_1                                                                  CGCTATGTATTTCGTACATTACTGC..................C......................................................................................................................................CCTCACCCACTAGGATACCA
riv25S2_1                                                                                           ..................C......................................................................................................................................
riv25S2_1                                                                                           ..................C......................................................................................................................................
riv25S2_1                                                                                           ..................C......................................................................................................................................
riv25S2_1                                                                                           ..................C......................................................................................................................................
riv25S2_1                                                                                           ..................C......................................................................................................................................
riv25S2_1                                                                                           ..................C......................................................................................................................................
riv25S2_1                                                                                           ..................C......................................................................................................................................
riv25S2_2                                                                                           ..................C......................................................................................................................................
riv25S2_2                                                                                           ..................C......................................................................................................................................
riv25S2_2                                                                                           ..................C......................................................................................................................................
riv25S2_2                                                                                           ..................C......................................................................................................................................
riv25S2_2                                                                                           ..................C......................................................................................................................................
riv25S2_2                                                                                           ..................C......................................................................................................................................
riv25S2_2                                                                                           ..................C......................................................................................................................................
riv25S2_2                                                                                           ..................C......................................................................................................................................
riv25S2_1                                                                                                                                                                                                                 CAACTATCACACATCAACTGCAA.........................................................................................................................................
riv25S2_1                                                                                                                                                                                                                                        .........................................................................................................................................
riv25S2_1                                                                                                                                                                                                                                        .........................................................................................................................................
riv25S2_1                                                                                                                                                                                                                                        .........................................................................................................................................
riv25S2_1                                                                                                                                                                                                                                        .........................................................................................................................................
riv25S2_1                                                                                                                                                                                                                                        .........................................................................................................................................
riv25S2_1                                                                                                                                                                                                                                        .........................................................................................................................................
riv25S2_1                                                                                                                                                                                                                                        .........................................................................................................................................
riv25S2_2                                                                                                                                                                                                                                        .........................................................................................................................................
riv25S2_2                                                                                                                                                                                                                                        .........................................................................................................................................
riv25S2_2                                                                                                                                                                                                                                        .........................................................................................................................................
riv25S2_2                                                                                                                                                                                                                                        .........................................................................................................................................
riv25S2_2                                                                                                                                                                                                                                        .........................................................................................................................................
riv25S2_2                                                                                                                                                                                                                                        .........................................................................................................................................
riv25S2_2                                                                                                                                                                                                                                        .........................................................................................................................................
riv25S2_2                                                                                                                                                                                                                                        .........................................................................................................................................

riv25S2 consensus
                TTCTTTCATGGGGAAGCAGATTTGGGTACCACCCAAGTATTGACTTACCCATCAACAACCGCTATGTATTTCGTACATTACTGCCAGCCACCATGAATATTGCACGGTACCATAAATACTTGACCACCTGTAGTACATAAAAACCCAATCCACATCAAAACCCCCTCCCCATGCTTACAAGCAAGTACAGCAATCAACCCTCAACTATCACACATCAACTGCAACTCCAAAGCCACCCCTCACCCACTAGGATACCAACAAACCTACCCACCCTTAACAGTACATAGTACATAAAGCCATTTACCGTACATAGCACATTACAGTCAAATCCCTTCTCGTCCCCATGGATGACCCCCCTCAG
```

```
                1111111111111111111111111111111111111111111111111111111111111111111111111111111111111111111111111111111111111111111111111111111111111111111111111111111111111111111111111111111111111111111111111111111111111111111111111111111111111111111111111111111111111111111111111111111111111111111111111111111111111111111111111111111111111111111111111111111111111111111
                6666666666666666666666666666666666666666666666666666666666666666666666666666666666666666666666666666666666666666666666666666666666666666666666666666666666666666666666666666666666666666666666666666666666666666666666666666666666666666666666666666666666666666666666666666666666666666666666666666666666666666666666666666666666666666666666666666666666666666666
                0000000000000000000000000000000000000000000000000000000000000000000000000000111111111111111111111111111111111111111111111111111111111111111111111111111111111111111111111111111111112222222222222222222222222222222222222222222222222222222222222222222222222222222222222222222222222222333333333333333333333333333333333333333333333333333333333333333333333333333333333
                2222223333333333444444444455555555556666666666777777777788888888889999999999000000000011111111112222222222333333333344444444445555555555666666666677777777778888888888999999999900000000001111111111222222222233333333334444444444555555555566666666667777777777888888888899999999990000000000111111111122222222223333333333444444444455555555556666666666777777777788888
                4567890123456789012345678901234567890123456789012345678901234567890123456789012345678901234567890123456789012345678901234567890123456789012345678901234567890123456789012345678901234567890123456789012345678901234567890123456789012345678901234567890123456789012345678901234567890123456789012345678901234567890123456789012345678901234567890123456789012345678901234

CRS             TTCTTTCATGGGGAAGCAGATTTGGGTACCACCCAAGTATTGACTCACCCATCAACAACCGCTATGTATTTCGTACATTACTGCCAGCCACCATGAATATTGTACGGTACCATAAATACTTGACCACCTGTAGTACATAAAAACCCAATCCACATCAAAACCCCCTCCCCATGCTTACAAGCAAGTACAGCAATCAACCCTCAACTATCACACATCAACTGCAACTCCAAAGCCACCCCTCACCCACTAGGATACCAACAAACCTACCCACCCTTAACAGTACATAGTACATAAAGCCATTTACCGTACATAGCACATTACAGTCAAATCCCTTCTCGTCCCCATGGATGACCCCCCTCAG

riv68_1         .........................................................................................................A..ACCATAAATACTTGACCACCTGTAG
riv68_1         .........................................................................................................A..
riv68_1         .........................................................................................................A..
riv68_1         .........................................................................................................A..
riv68_1         .........................................................................................................A..
riv68_1         .........................................................................................................A..
riv68_1         .........................................................................................................A..
riv68_1         .........................................................................................................A..
riv68_2         .........................................................................................................A..
riv68_2         .........................................................................................................A..
riv68_2         .........................................................................................................A..
riv68_2         .........................................................................................................A..
riv68_2         .........................................................................................................A..
riv68_2         .........................................................................................................A..
riv68_2         .........................................................................................................A..
riv68_2         .........................................................................................................A..
riv68_1                                                                    CGCTATGTATTTCGTACATTACTGC.....................A...................................................................................................................................CCTCACCCACTAGGATACCA
riv68_1                                                                                             .....................A...................................................................................................................................
riv68_1                                                                                             .....................A...................................................................................................................................
riv68_1                                                                                             .....................A...................................................................................................................................
riv68_1                                                                                             .....................A...................................................................................................................................
riv68_1                                                                                             .....................A...................................................................................................................................
riv68_1                                                                                             .....................A..................T........................................C.......................................................................
riv68_1                                                                                             .....................A...................................................................................................................................
riv68_2                                                                                             .....................A...................................................................................................................................
riv68_2                                                                                             .....................A...................................................................................................................................
riv68_2                                                                                             .....................A...................................................................................................................................
riv68_2                                                                                             .....................A...........................................................C.......................................................................
riv68_2                                                                                             .....................A...................................................................................................................................
riv68_2                                                                                             .....................A...................................................................................................................................
riv68_2                                                                                             .....................A...................................................................................................................................
riv68_2                                                                                             .....................A...................................................................................................................................
riv68_1                                                                                                                                                                                                                   CAACTATCACACATCAACTGCAA............................................................................................................C............................
riv68_1                                                                                                                                                                                                                                          ............................................................................................................C...........................A
riv68_1                                                                                                                                                                                                                                          ............................................................................................................C............................
riv68_1                                                                                                                                                                                                                                          ............................................................................................................C............................
riv68_1                                                                                                                                                                                                                                          ............................................................................................................C............................
riv68_1                                                                                                                                                                                                                                          ............................................................................................................C............................
riv68_1                                                                                                                                                                                                                                          ............................................................................................................C............................
riv68_1                                                                                                                                                                                                                                          ............................................................................................................C............................
riv68_2                                                                                                                                                                                                                                          ............................................................................................................C............................
riv68_2                                                                                                                                                                                                                                          ............................................................................................................C............................
riv68_2                                                                                                                                                                                                                                          ............................................................................................................C............................
riv68_2                                                                                                                                                                                                                                          ............................................................................................................C............................
riv68_2                                                                                                                                                                                                                                          ............................................................................................................C............................
riv68_2                                                                                                                                                                                                                                          ............................................................................................................C............................
riv68_2                                                                                                                                                                                                                                          ............................................................................................................C............................
riv68_2                                                                                                                                                                                                                                          ............................................................................................................C............................

riv68 consensus
                TTCTTTCATGGGGAAGCAGATTTGGGTACCACCCAAGTATTGACTCACCCATCAACAACCGCTATGTATTTCGTACATTACTGCCAGCCACCATGAATATTGTACAGTACCATAAATACTTGACCACCTGTAGTACATAAAAACCCAATCCACATCAAAACCCCCTCCCCATGCTTACAAGCAAGTACAGCAATCAACCCTCAACTATCACACATCAACTGCAACTCCAAAGCCACCCCTCACCCACTAGGATACCAACAAACCTACCCACCCTTAACAGTACATAGTACATAAAGCCATTTACCGTACATAGCACATTACAGTCAAATCCCCTCTCGTCCCCATGGATGACCCCCCTCAG
```

```
                1111111111111111111111111111111111111111111111111111111111111111111111111111111111111111111111111111111111111111111111111111111111111111111111111111111111111111111111111111111111111111111111111111111111111111111111111111111111111111111111111111111111111111111111111111111111111111111111111111111111111111111111111111111111111111111111111111111111111111111
                6666666666666666666666666666666666666666666666666666666666666666666666666666666666666666666666666666666666666666666666666666666666666666666666666666666666666666666666666666666666666666666666666666666666666666666666666666666666666666666666666666666666666666666666666666666666666666666666666666666666666666666666666666666666666666666666666666666666666666666
                0000000000000000000000000000000000000000000000000000000000000000000000000000111111111111111111111111111111111111111111111111111111111111111111111111111111111111111111111111111122222222222222222222222222222222222222222222222222222222222222222222222222222222222222222222222222223333333333333333333333333333333333333333333333333333333333333333333333333333333333333
                2222223333333333444444444455555555556666666666777777777788888888889999999999000000000011111111112222222222333333333344444444445555555555666666666677777777778888888888999999999900000000001111111111222222222233333333334444444444555555555566666666667777777777888888888899999999990000000000111111111122222222223333333333444444444455555555556666666666777777777788888
                4567890123456789012345678901234567890123456789012345678901234567890123456789012345678901234567890123456789012345678901234567890123456789012345678901234567890123456789012345678901234567890123456789012345678901234567890123456789012345678901234567890123456789012345678901234567890123456789012345678901234567890123456789012345678901234567890123456789012345678901234

CRS             TTCTTTCATGGGGAAGCAGATTTGGGTACCACCCAAGTATTGACTCACCCATCAACAACCGCTATGTATTTCGTACATTACTGCCAGCCACCATGAATATTGTACGGTACCATAAATACTTGACCACCTGTAGTACATAAAAACCCAATCCACATCAAAACCCCCTCCCCATGCTTACAAGCAAGTACAGCAATCAACCCTCAACTATCACACATCAACTGCAACTCCAAAGCCACCCCTCACCCACTAGGATACCAACAAACCTACCCACCCTTAACAGTACATAGTACATAAAGCCATTTACCGTACATAGCACATTACAGTCAAATCCCTTCTCGTCCCCATGGATGACCCCCCTCAG

riv70_1         ............................................................................................................ACCATAAATACTTGACCACCTGTAG
riv70_1         ...........................................T................................................................
riv70_1         ............................................................................................................
riv70_1         ............................................................................................................
riv70_1         ............................................................................................................
riv70_1         ............................................................................................................
riv70_1         ............................................................................................................
riv70_1         ............................................................................................................
riv70_2         ............................................................................................................
riv70_2         ............................................................................................................
riv70_2         ............................................................................................................
riv70_2         ............................................................................................................
riv70_2         ............................................................................................................
riv70_2         ............................................................................................................
riv70_2         ............................................................................................................
riv70_2         ............................................................................................................
riv70_1                                                                    CGCTATGTATTTCGTACATTACTGC.........................................................................................................................................................CCTCACCCACTAGGATACCA
riv70_1                                                                                             .........................................................................................................................................................
riv70_1                                                                                             .........................................................................................................................................................
riv70_1                                                                                             .........................................................................................................................................................
riv70_1                                                                                             .........................................................................................................................................................
riv70_1                                                                                             .........................................................................................................................................................
riv70_1                                                                                             .........................................................................................................................................................
riv70_1                                                                                             .........................................................................................................................................................
riv70_2                                                                                             .........................................................................................................................................................
riv70_2                                                                                             .........................................................................................................................................................
riv70_2                                                                                             .........................................................................................................................................................
riv70_2                                                                                             .........................................................................................................................................................
riv70_2                                                                                             .........................................................................................................................................................
riv70_2                                                                                             .........................................................................................................................................................
riv70_2                                                                                             .........................................................................................................................................................
riv70_2                                                                                             .........................................................................................................................................................
riv70_1                                                                                                                                                                                                                         CAACTATCACACATCAACTGCAA.........................................................................................................................................
riv70_1                                                                                                                                                                                                                                                 .........................................................................................................................................
riv70_1                                                                                                                                                                                                                                                 .........................................................................................................................................
riv70_1                                                                                                                                                                                                                                                 .........................................................................................................................................
riv70_1                                                                                                                                                                                                                                                 .........................................................................................................................................
riv70_1                                                                                                                                                                                                                                                 .........................................................................................................................................
riv70_1                                                                                                                                                                                                                                                 .........................................................................................................................................
riv70_1                                                                                                                                                                                                                                                 .........................................................................................................................................
riv70_2                                                                                                                                                                                                                                                 .........................................................................................................................................
riv70_2                                                                                                                                                                                                                                                 .........................................................................................................................................
riv70_2                                                                                                                                                                                                                                                 .........................................................................................................................................
riv70_2                                                                                                                                                                                                                                                 .........................................................................................................................................
riv70_2                                                                                                                                                                                                                                                 .........................................................................................................................................
riv70_2                                                                                                                                                                                                                                                 .........................................................................................................................................
riv70_2                                                                                                                                                                                                                                                 .........................................................................................................................................
riv70_2                                                                                                                                                                                                                                                 .........................................................................................................................................

riv70 consensus
                TTCTTTCATGGGGAAGCAGATTTGGGTACCACCCAAGTATTGACTCACCCATCAACAACCGCTATGTATTTCGTACATTACTGCCAGCCACCATGAATATTGTACGGTACCATAAATACTTGACCACCTGTAGTACATAAAAACCCAATCCACATCAAAACCCCCTCCCCATGCTTACAAGCAAGTACAGCAATCAACCCTCAACTATCACACATCAACTGCAACTCCAAAGCCACCCCTCACCCACTAGGATACCAACAAACCTACCCACCCTTAACAGTACATAGTACATAAAGCCATTTACCGTACATAGCACATTACAGTCAAATCCCTTCTCGTCCCCATGGATGACCCCCCTCAG
```

```
                1111111111111111111111111111111111111111111111111111111111111111111111111111111111111111111111111111111111111111111111111111111111111111111111111111111111111111111111111111111111111111111111111111111111111111111111111111111111111111111111111111111111111111111111111111111111111111111111111111111111111111111111111111111111111111111111111111111111111111111
                6666666666666666666666666666666666666666666666666666666666666666666666666666666666666666666666666666666666666666666666666666666666666666666666666666666666666666666666666666666666666666666666666666666666666666666666666666666666666666666666666666666666666666666666666666666666666666666666666666666666666666666666666666666666666666666666666666666666666666666
                0000000000000000000000000000000000000000000000000000000000000000000000000000111111111111111111111111111111111111111111111111111111111111111111111111111111111111111111111111111122222222222222222222222222222222222222222222222222222222222222222222222222222222222222222222222222223333333333333333333333333333333333333333333333333333333333333333333333333333333333333
                2222223333333333444444444455555555556666666666777777777788888888889999999999000000000011111111112222222222333333333344444444445555555555666666666677777777778888888888999999999900000000001111111111222222222233333333334444444444555555555566666666667777777777888888888899999999990000000000111111111122222222223333333333444444444455555555556666666666777777777788888
                4567890123456789012345678901234567890123456789012345678901234567890123456789012345678901234567890123456789012345678901234567890123456789012345678901234567890123456789012345678901234567890123456789012345678901234567890123456789012345678901234567890123456789012345678901234567890123456789012345678901234567890123456789012345678901234567890123456789012345678901234

CRS             TTCTTTCATGGGGAAGCAGATTTGGGTACCACCCAAGTATTGACTCACCCATCAACAACCGCTATGTATTTCGTACATTACTGCCAGCCACCATGAATATTGTACGGTACCATAAATACTTGACCACCTGTAGTACATAAAAACCCAATCCACATCAAAACCCCCTCCCCATGCTTACAAGCAAGTACAGCAATCAACCCTCAACTATCACACATCAACTGCAACTCCAAAGCCACCCCTCACCCACTAGGATACCAACAAACCTACCCACCCTTAACAGTACATAGTACATAAAGCCATTTACCGTACATAGCACATTACAGTCAAATCCCTTCTCGTCCCCATGGATGACCCCCCTCAG

riv85_1         ............................................................................................................ACCATAAATACTTGACCACCTGTAG
riv85_1         ............................................................................................................
riv85_1         ............................................................................................................
riv85_1         ............................................................................................................
riv85_1         ............................................................................................................
riv85_1         ............................................................................................................
riv85_1         ............................................................................................................
riv85_1         ............................................................................................................
riv85_2         ............................................................................................................
riv85_2         ............................................................................................................
riv85_2         ............................................................................................................
riv85_2         ............................................................................................................
riv85_2         ............................................................................................................
riv85_2         ............................................................................................................
riv85_2         ............................................................................................................
riv85_2         ............................................................................................................
riv85_1                                                                    CGCTATGTATTTCGTACATTACTGC.........................................................................................................................................................CCTCACCCACTAGGATACCA
riv85_1                                                                                             .........................................................................................................................................................
riv85_1                                                                                             .........................................................................................................................................................
riv85_1                                                                                             .........................................................................................................................................................
riv85_1                                                                                             .........................................................................................................................................................
riv85_1                                                                                             .........................................................................................................................................................
riv85_1                                                                                             .........................................................................................................................................................
riv85_1                                                                                             .........................................................................................................................................................
riv85_2                                                                                             .........................................................................................................................................................
riv85_2                                                                                             .........................................................................................................................................................
riv85_2                                                                                             .........................................................................................................................................................
riv85_2                                                                                             .........................................................................................................................................................
riv85_2                                                                                             .........................................................................................................................................................
riv85_2                                                                                             .........................................................................................................................................................
riv85_2                                                                                             .........................................................................................................................................................
riv85_2                                                                                             .........................................................................................................................................................
riv85_1                                                                                                                                                                                                                         CAACTATCACACATCAACTGCAA.........................................................................................................................................
riv85_1                                                                                                                                                                                                                                                 .........................................................................................................................................
riv85_1                                                                                                                                                                                                                                                 .........................................................................................................................................
riv85_1                                                                                                                                                                                                                                                 .........................................................................................................................................
riv85_1                                                                                                                                                                                                                                                 .........................................................................................................................................
riv85_1                                                                                                                                                                                                                                                 .........................................................................................................................................
riv85_1                                                                                                                                                                                                                                                 .........................................................................................................................................
riv85_1                                                                                                                                                                                                                                                 .........................................................................................................................................
riv85_2                                                                                                                                                                                                                                                 .........................................................................................................................................
riv85_2                                                                                                                                                                                                                                                 .........................................................................................................................................
riv85_2                                                                                                                                                                                                                                                 .........................................................................................................................................
riv85_2                                                                                                                                                                                                                                                 .........................................................................................................................................
riv85_2                                                                                                                                                                                                                                                 .........................................................................................................................................
riv85_2                                                                                                                                                                                                                                                 .........................................................................................................................................
riv85_2                                                                                                                                                                                                                                                 .........................................................................................................................................
riv85_2                                                                                                                                                                                                                                                 .........................................................................................................................................

riv85 consensus
                TTCTTTCATGGGGAAGCAGATTTGGGTACCACCCAAGTATTGACTCACCCATCAACAACCGCTATGTATTTCGTACATTACTGCCAGCCACCATGAATATTGTACGGTACCATAAATACTTGACCACCTGTAGTACATAAAAACCCAATCCACATCAAAACCCCCTCCCCATGCTTACAAGCAAGTACAGCAATCAACCCTCAACTATCACACATCAACTGCAACTCCAAAGCCACCCCTCACCCACTAGGATACCAACAAACCTACCCACCCTTAACAGTACATAGTACATAAAGCCATTTACCGTACATAGCACATTACAGTCAAATCCCTTCTCGTCCCCATGGATGACCCCCCTCAG
```

```
               1111111111111111111111111111111111111111111111111111111111111111111111111111111111111111111111111111111111111111111111111111111111111111111111111111111111111111111111111111111111111111111111111111111111111111111111111111111111111111111111111111111111111111111111111111111111111111111111111111111111111111111111111111111111111111111111111111111111111111111
               6666666666666666666666666666666666666666666666666666666666666666666666666666666666666666666666666666666666666666666666666666666666666666666666666666666666666666666666666666666666666666666666666666666666666666666666666666666666666666666666666666666666666666666666666666666666666666666666666666666666666666666666666666666666666666666666666666666666666666666
               0000000000000000000000000000000000000000000000000000000000000000000000000000111111111111111111111111111111111111111111111111111111111111111111111111111111111111111111111111111122222222222222222222222222222222222222222222222222222222222222222222222222222222222222222222222222223333333333333333333333333333333333333333333333333333333333333333333333333333333333333
               2222223333333333444444444455555555556666666666777777777788888888889999999999000000000011111111112222222222333333333344444444445555555555666666666677777777778888888888999999999900000000001111111111222222222233333333334444444444555555555566666666667777777777888888888899999999990000000000111111111122222222223333333333444444444455555555556666666666777777777788888
               4567890123456789012345678901234567890123456789012345678901234567890123456789012345678901234567890123456789012345678901234567890123456789012345678901234567890123456789012345678901234567890123456789012345678901234567890123456789012345678901234567890123456789012345678901234567890123456789012345678901234567890123456789012345678901234567890123456789012345678901234

CRS            TTCTTTCATGGGGAAGCAGATTTGGGTACCACCCAAGTATTGACTCACCCATCAACAACCGCTATGTATTTCGTACATTACTGCCAGCCACCATGAATATTGTACGGTACCATAAATACTTGACCACCTGTAGTACATAAAAACCCAATCCACATCAAAACCCCCTCCCCATGCTTACAAGCAAGTACAGCAATCAACCCTCAACTATCACACATCAACTGCAACTCCAAAGCCACCCCTCACCCACTAGGATACCAACAAACCTACCCACCCTTAACAGTACATAGTACATAAAGCCATTTACCGTACATAGCACATTACAGTCAAATCCCTTCTCGTCCCCATGGATGACCCCCCTCAG

riv90_1        ...........................G.............................................................................C..ACCATAAATACTTGACCACCTGTAG
riv90_1        ...........................G.............................................................................C..
riv90_1        ...........................G.............................................................................C..
riv90_1        ...........................G.............................................................................C..
riv90_1        ...........................G.............................................................................C..
riv90_1        ...........................G.............................................................................C..
riv90_1        ...........................G.............................................................................C..
riv90_1        ...........................G.............................................................................C..
riv90_2        ...........................G.............................................................................C..
riv90_2        ...........................G.............................................................................C..
riv90_2        ...........................G.............................................................................C..
riv90_2        ...........................G.............................................................................C..
riv90_2        ...........................G.............................................................................C..
riv90_2        ...........................G.............................................................................C..
riv90_2        ...........................G.............................................................................C..
riv90_2        ...........................G.............................................................................C..
riv90_1                                                                   CGCTATGTATTTCGTACATTACTGC.....................C..............................................................................................................................T....CCTCACCCACTAGGATACCA
riv90_1                                                                                            .....................C..............................................................................................................................T....
riv90_1                                                                                            .....................C..............................................................................................................................T....
riv90_1                                                                                            .....................C..............................................................................................................................T....
riv90_1                                                                                            .....................C..............................................................................................................................T....
riv90_1                                                                                            .....................C..............................................................................................................................T....
riv90_1                                                                                            .....................C..............................................................................................................................T....
riv90_1                                                                                            .....................C..............................................................................................................................T....
riv90_2                                                                                            .....................C..............................................................................................................................T....
riv90_2                                                                                            .....................C..............................................................................................................................T....
riv90_2                                                                                            .....................C..............................................................................................................................T....
riv90_2                                                                                            .....................C..............................................................................................................................T....
riv90_2                                                                                            .....................C..............................................................................................................................T....
riv90_2                                                                                            .....................C..............................................................................................................................T....
riv90_2                                                                                            .....................C..............................................................................................................................T....
riv90_2                                                                                            .....................C..............................................................................................................................T....
riv90_1                                                                                                                                                                                                                    CAACTATCACACATCAACTGCAA........T................................................................................................................................
riv90_1                                                                                                                                                                                                                                           ........T................................................................................................................................
riv90_1                                                                                                                                                                                                                                           ........T................................................................................................................................
riv90_1                                                                                                                                                                                                                                           ........T................................................................................................................................
riv90_1                                                                                                                                                                                                                                           ........T................................................................................................................................
riv90_1                                                                                                                                                                                                                                           ........T................................................................................................................................
riv90_1                                                                                                                                                                                                                                           ........T................................................................................................................................
riv90_1                                                                                                                                                                                                                                           ........T................................................................................................................................
riv90_2                                                                                                                                                                                                                                           ........T................................................................................................................................
riv90_2                                                                                                                                                                                                                                           ........T................................................................................................................................
riv90_2                                                                                                                                                                                                                                           ........T................................................................................................................................
riv90_2                                                                                                                                                                                                                                           ........T................................................................................................................................
riv90_2                                                                                                                                                                                                                                           ........T................................................................................................................................
riv90_2                                                                                                                                                                                                                                           ........T................................................................................................................................
riv90_2                                                                                                                                                                                                                                           ........T................................................................................................................................
riv90_2                                                                                                                                                                                                                                           ........T................................................................................................................................

riv90 consensus
               TTCTTTCATGGGGAAGCAGATTTGGGTGCCACCCAAGTATTGACTCACCCATCAACAACCGCTATGTATTTCGTACATTACTGCCAGCCACCATGAATATTGTACCGTACCATAAATACTTGACCACCTGTAGTACATAAAAACCCAATCCACATCAAAACCCCCTCCCCATGCTTACAAGCAAGTACAGCAATCAACCCTCAACTATCACACATCAACTGCAACTCCAAAGTCACCCCTCACCCACTAGGATACCAACAAACCTACCCACCCTTAACAGTACATAGTACATAAAGCCATTTACCGTACATAGCACATTACAGTCAAATCCCTTCTCGTCCCCATGGATGACCCCCCTCAG


               1111111111111111111111111111111111111111111111111111111111111111111111111111111111111111111111111111111111111111111111111111111111111111111111111111111111111111111111111111111111111111111111111111111111111111111111111111111111111111111111111111111111111111111111111111111111111111111111111111111111111111111111111111111111111111111111111111111111111111111
               6666666666666666666666666666666666666666666666666666666666666666666666666666666666666666666666666666666666666666666666666666666666666666666666666666666666666666666666666666666666666666666666666666666666666666666666666666666666666666666666666666666666666666666666666666666666666666666666666666666666666666666666666666666666666666666666666666666666666666666
               0000000000000000000000000000000000000000000000000000000000000000000000000000111111111111111111111111111111111111111111111111111111111111111111111111111111111111111111111111111122222222222222222222222222222222222222222222222222222222222222222222222222222222222222222222222222223333333333333333333333333333333333333333333333333333333333333333333333333333333333333
               2222223333333333444444444455555555556666666666777777777788888888889999999999000000000011111111112222222222333333333344444444445555555555666666666677777777778888888888999999999900000000001111111111222222222233333333334444444444555555555566666666667777777777888888888899999999990000000000111111111122222222223333333333444444444455555555556666666666777777777788888
               4567890123456789012345678901234567890123456789012345678901234567890123456789012345678901234567890123456789012345678901234567890123456789012345678901234567890123456789012345678901234567890123456789012345678901234567890123456789012345678901234567890123456789012345678901234567890123456789012345678901234567890123456789012345678901234567890123456789012345678901234

CRS            TTCTTTCATGGGGAAGCAGATTTGGGTACCACCCAAGTATTGACTCACCCATCAACAACCGCTATGTATTTCGTACATTACTGCCAGCCACCATGAATATTGTACGGTACCATAAATACTTGACCACCTGTAGTACATAAAAACCCAATCCACATCAAAACCCCCTCCCCATGCTTACAAGCAAGTACAGCAATCAACCCTCAACTATCACACATCAACTGCAACTCCAAAGCCACCCCTCACCCACTAGGATACCAACAAACCTACCCACCCTTAACAGTACATAGTACATAAAGCCATTTACCGTACATAGCACATTACAGTCAAATCCCTTCTCGTCCCCATGGATGACCCCCCTCAG

riv103_1       .........................................................................................................A..ACCATAAATACTTGACCACCTGTAG
riv103_1       .........................................................................................................A..
riv103_1       .........................................................................................................A..
riv103_1       .........................................................................................................A..
riv103_1       .........................................................................................................A..
riv103_1       .........................................................................................................A..
riv103_1       .........................................................................................................A..
riv103_1       .........................................................................................................A..
riv103_2       .........................................................................................................A..
riv103_2       .........................................................................................................A..
riv103_2       .........................................................................................................A..
riv103_2       .........................................................................................................A..
riv103_2       .........................................................................................................A..
riv103_2       .........................................................................................................A..
riv103_2       .........................................................................................................A..
riv103_2       .........................................................................................................A..
riv103_1                                                                  CGCTATGTATTTCGTACATTACTGC.....................A..............................................................................................................................T....CCTCACCCACTAGGATACCA
riv103_1                                                                                           .....................A..............................................................................................................................T....
riv103_1                                                                                           .....................A..............................................................................................................................T....
riv103_1                                                                                           .....................A..............................................................................................................................T....
riv103_1                                                                                           .....................A..............................................................................................................................T....
riv103_1                                                                                           .....................A..............................................................................................................................T....
riv103_1                                                                                           .....................A..............................................................................................................................T....
riv103_1                                                                                           .....................A..............................................................................................................................T....
riv103_2                                                                                           .....................A..............................................................................................................................T....
riv103_2                                                                                           .....................A..............................................................................................................................T....
riv103_2                                                                                           .....................A..............................................................................................................................T....
riv103_2                                                                                           .....................A..............................................................................................................................T....
riv103_2                                                                                           .....................A..............................................................................................................................T....
riv103_2                                                                                           .....................A..............................................................................................................................T....
riv103_2                                                                                           .....................A..............................................................................................................................T....
riv103_2                                                                                           .....................A..............................................................................................................................T....
riv103_1                                                                                                                                                                                                                   CAACTATCACACATCAACTGCAA........T.............T.......................T..........................................................................................
riv103_1                                                                                                                                                                                                                                          ........T.............T.......................T..........................................................................................
riv103_1                                                                                                                                                                                                                                          ........T.............T.......................T..........................................................................................
riv103_1                                                                                                                                                                                                                                          ........T.............T.......................T..........................................................................................
riv103_1                                                                                                                                                                                                                                          ........T.............T.......................T..........................................................................................
riv103_1                                                                                                                                                                                                                                          ........T.............T.......................T..........................................................................................
riv103_1                                                                                                                                                                                                                                          ........T.............T.......................T..........................................................................................
riv103_1                                                                                                                                                                                                                                          ........T.............T.......................T..........................................................................................
riv103_2                                                                                                                                                                                                                                          ........T.............T.......................T..........................................................................................
riv103_2                                                                                                                                                                                                                                          ........T.............T.......................T..........................................................................................
riv103_2                                                                                                                                                                                                                                          ........T.............T.......................T..........................................................................................
riv103_2                                                                                                                                                                                                                                          ........T.............T.......................T..........................................................................................
riv103_2                                                                                                                                                                                                                                          ........T.............T.......................T..........................................................................................
riv103_2                                                                                                                                                                                                                                          ........T.............T.......................T..........................................................................................
riv103_2                                                                                                                                                                                                                                          ........T.............T.......................T..........................................................................................
riv103_2                                                                                                                                                                                                                                          ........T.............T.......................T..........................................................................................

riv103 consensus
               TTCTTTCATGGGGAAGCAGATTTGGGTACCACCCAAGTATTGACTCACCCATCAACAACCGCTATGTATTTCGTACATTACTGCCAGCCACCATGAATATTGTACAGTACCATAAATACTTGACCACCTGTAGTACATAAAAACCCAATCCACATCAAAACCCCCTCCCCATGCTTACAAGCAAGTACAGCAATCAACCCTCAACTATCACACATCAACTGCAACTCCAAAGTCACCCCTCACCCATTAGGATACCAACAAACCTACCCATCCTTAACAGTACATAGTACATAAAGCCATTTACCGTACATAGCACATTACAGTCAAATCCCTTCTCGTCCCCATGGATGACCCCCCTCAG
```

```
                  1111111111111111111111111111111111111111111111111111111111111111111111111111111111111111111111111111111111111111111111111111111111111111111111111111111111111111111111111111111111111111111111111111111111111111111111111111111111111111111111111111111111111111111111111111111111111111111111111111111111111111111111111111111111111111111111111111111111111111111111
                  6666666666666666666666666666666666666666666666666666666666666666666666666666666666666666666666666666666666666666666666666666666666666666666666666666666666666666666666666666666666666666666666666666666666666666666666666666666666666666666666666666666666666666666666666666666666666666666666666666666666666666666666666666666666666666666666666666666666666666666666
                  0000000000000000000000000000000000000000000000000000000000000000000000000000111111111111111111111111111111111111111111111111111111111111111111111111111111111111111111111111111122222222222222222222222222222222222222222222222222222222222222222222222222222222222222222222222222223333333333333333333333333333333333333333333333333333333333333333333333333333333333333
                  2222223333333333444444444455555555556666666666777777777788888888889999999999000000000011111111112222222222333333333344444444445555555555666666666677777777778888888888999999999900000000001111111111222222222233333333334444444444555555555566666666667777777777888888888899999999990000000000111111111122222222223333333333444444444455555555556666666666777777777788888
                  4567890123456789012345678901234567890123456789012345678901234567890123456789012345678901234567890123456789012345678901234567890123456789012345678901234567890123456789012345678901234567890123456789012345678901234567890123456789012345678901234567890123456789012345678901234567890123456789012345678901234567890123456789012345678901234567890123456789012345678901234

CRS               TTCTTTCATGGGGAAGCAGATTTGGGTACCACCCAAGTATTGACTCACCCATCAACAACCGCTATGTATTTCGTACATTACTGCCAGCCACCATGAATATTGTACGGTACCATAAATACTTGACCACCTGTAGTACATAAAAACCCAATCCACATCAAAACCCCCTCCCCATGCTTACAAGCAAGTACAGCAATCAACCCTCAACTATCACACATCAACTGCAACTCCAAAGCCACCCCTCACCCACTAGGATACCAACAAACCTACCCACCCTTAACAGTACATAGTACATAAAGCCATTTACCGTACATAGCACATTACAGTCAAATCCCTTCTCGTCCCCATGGATGACCCCCCTCAG

riv116_1          ............................................................................................................ACCATAAATACTTGACCACCTGTAG
riv116_1          ............................................................................................................
riv116_1          ............................................................................................................
riv116_1          ............................................................................................................
riv116_1          ............................................................................................................
riv116_1          ............................................................................................................
riv116_1          ............................................................................................................
riv116_1          ............................................................................................................
riv116_2          ............................................................................................................
riv116_2          ............................................................................................................
riv116_2          ............................................................................................................
riv116_2          ............................................................................................................
riv116_2          ............................................................................................................
riv116_2          ............................................................................................................
riv116_2          ............................................................................................................
riv116_2          ............................................................................................................
riv116_1                                                                     CGCTATGTATTTCGTACATTACTGC.........................................................................................................................................................CCTCACCCACTAGGATACCA
riv116_1                                                                                          .........................................................................................................................................................
riv116_1                                                                                          .........................................................................................................................................................
riv116_1                                                                                          .........................................................................................................................................................
riv116_1                                                                                          .........................................................................................................................................................
riv116_1                                                                                          .........................................................................................................................................................
riv116_1                                                                                          .........................................................................................................................................................
riv116_1                                                                                          .........................................................................................................................................................
riv116_2                                                                                          .........................................................................................................................................................
riv116_2                                                                                          .........................................................................................................................................................
riv116_2                                                                                          .........................................................................................................................................................
riv116_2                                                                                          .........................................................................................................................................................
riv116_2                                                                                          .........................................................................................................................................................
riv116_2                                                                                          .........................................................................................................................................................
riv116_2                                                                                          .........................................................................................................................................................
riv116_2                                                                                          .........................................................................................................................................................
riv116_1                                                                                                                                                                                                                   CAACTATCACACATCAACTGCAA.........................................................................................................................................
riv116_1                                                                                                                                                                                                                                          .........................................................................................................................................
riv116_1                                                                                                                                                                                                                                          .........................................................................................................................................
riv116_1                                                                                                                                                                                                                                          .........................................................................................................................................
riv116_1                                                                                                                                                                                                                                          .........................................................................................................................................
riv116_1                                                                                                                                                                                                                                          .........................................................................................................................................
riv116_1                                                                                                                                                                                                                                          .........................................................................................................................................
riv116_1                                                                                                                                                                                                                                          .........................................................................................................................................
riv116_2                                                                                                                                                                                                                                          .........................................................................................................................................
riv116_2                                                                                                                                                                                                                                          .........................................................................................................................................
riv116_2                                                                                                                                                                                                                                          .........................................................................................................................................
riv116_2                                                                                                                                                                                                                                          .........................................................................................................................................
riv116_2                                                                                                                                                                                                                                          .........................................................................................................................................
riv116_2                                                                                                                                                                                                                                          .........................................................................................................................................
riv116_2                                                                                                                                                                                                                                          .........................................................................................................................................
riv116_2                                                                                                                                                                                                                                          .........................................................................................................................................

riv116 consensus
                  TTCTTTCATGGGGAAGCAGATTTGGGTACCACCCAAGTATTGACTCACCCATCAACAACCGCTATGTATTTCGTACATTACTGCCAGCCACCATGAATATTGTACGGTACCATAAATACTTGACCACCTGTAGTACATAAAAACCCAATCCACATCAAAACCCCCTCCCCATGCTTACAAGCAAGTACAGCAATCAACCCTCAACTATCACACATCAACTGCAACTCCAAAGCCACCCCTCACCCACTAGGATACCAACAAACCTACCCACCCTTAACAGTACATAGTACATAAAGCCATTTACCGTACATAGCACATTACAGTCAAATCCCTTCTCGTCCCCATGGATGACCCCCCTCAG
```

```
                  1111111111111111111111111111111111111111111111111111111111111111111111111111111111111111111111111111111111111111111111111111111111111111111111111111111111111111111111111111111111111111111111111111111111111111111111111111111111111111111111111111111111111111111111111111111111111111111111111111111111111111111111111111111111111111111111111111111111111111111111
                  6666666666666666666666666666666666666666666666666666666666666666666666666666666666666666666666666666666666666666666666666666666666666666666666666666666666666666666666666666666666666666666666666666666666666666666666666666666666666666666666666666666666666666666666666666666666666666666666666666666666666666666666666666666666666666666666666666666666666666666666
                  0000000000000000000000000000000000000000000000000000000000000000000000000000111111111111111111111111111111111111111111111111111111111111111111111111111111111111111111111111111122222222222222222222222222222222222222222222222222222222222222222222222222222222222222222222222222223333333333333333333333333333333333333333333333333333333333333333333333333333333333333
                  2222223333333333444444444455555555556666666666777777777788888888889999999999000000000011111111112222222222333333333344444444445555555555666666666677777777778888888888999999999900000000001111111111222222222233333333334444444444555555555566666666667777777777888888888899999999990000000000111111111122222222223333333333444444444455555555556666666666777777777788888
                  4567890123456789012345678901234567890123456789012345678901234567890123456789012345678901234567890123456789012345678901234567890123456789012345678901234567890123456789012345678901234567890123456789012345678901234567890123456789012345678901234567890123456789012345678901234567890123456789012345678901234567890123456789012345678901234567890123456789012345678901234

CRS               TTCTTTCATGGGGAAGCAGATTTGGGTACCACCCAAGTATTGACTCACCCATCAACAACCGCTATGTATTTCGTACATTACTGCCAGCCACCATGAATATTGTACGGTACCATAAATACTTGACCACCTGTAGTACATAAAAACCCAATCCACATCAAAACCCCCTCCCCATGCTTACAAGCAAGTACAGCAATCAACCCTCAACTATCACACATCAACTGCAACTCCAAAGCCACCCCTCACCCACTAGGATACCAACAAACCTACCCACCCTTAACAGTACATAGTACATAAAGCCATTTACCGTACATAGCACATTACAGTCAAATCCCTTCTCGTCCCCATGGATGACCCCCCTCAG

mom7_1            ............................................................................................................ACCATAAATACTTGACCACCTGTAG
mom7_1            ............................................................................................................
mom7_1            ............................................................................................................
mom7_1            ............................................................................................................
mom7_1            ............................................................................................................
mom7_1            ............................................................................................................
mom7_1            ............................................................................................................
mom7_1            ............................................................................................................
mom7_2            ............................................................................................................
mom7_2            ............................................................................................................
mom7_2            ............................................................................................................
mom7_2            ............................................................................................................
mom7_2            ............................................................................................................
mom7_2            ............................................................................................................
mom7_2            ............................................................................................................
mom7_2            ............................................................................................................
mom7_1                                                                       CGCTATGTATTTCGTACATTACTGC.........................................................................................................................................................CCTCACCCACTAGGATACCA
mom7_1                                                                                            .........................................................................................................................................................
mom7_1                                                                                            .........................................................................................................................................................
mom7_1                                                                                            .........................................................................................................................................................
mom7_1                                                                                            .........................................................................................................................................................
mom7_1                                                                                            .........................................................................................................................................................
mom7_1                                                                                            .........................................................................................................................................................
mom7_1                                                                                            .........................................................................................................................................................
mom7_2                                                                                            .........................................................................................................................................................
mom7_2                                                                                            .........................................................................................................................................................
mom7_2                                                                                            .........................................................................................................................................................
mom7_2                                                                                            .........................................................................................................................................................
mom7_2                                                                                            .........................................................................................................................................................
mom7_2                                                                                            .........................................................................................................................................................
mom7_2                                                                                            .........................................................................................................................................................
mom7_2                                                                                            .........................................................................................................................................................
mom7_1                                                                                                                                                                                                                   CAACTATCACACATCAACTGCAA.........................................................................................................................................
mom7_1                                                                                                                                                                                                                                          .........................................................................................................................................
mom7_1                                                                                                                                                                                                                                          .........................................................................................................................................
mom7_1                                                                                                                                                                                                                                          .........................................................................................................................................
mom7_1                                                                                                                                                                                                                                          .........................................................................................................................................
mom7_1                                                                                                                                                                                                                                          .........................................................................................................................................
mom7_1                                                                                                                                                                                                                                          .........................................................................................................................................
mom7_1                                                                                                                                                                                                                                          .........................................................................................................................................
mom7_2                                                                                                                                                                                                                                          .........................................................................................................................................
mom7_2                                                                                                                                                                                                                                          .........................................................................................................................................
mom7_2                                                                                                                                                                                                                                          .........................................................................................................................................
mom7_2                                                                                                                                                                                                                                          .........................................................................................................................................
mom7_2                                                                                                                                                                                                                                          .........................................................................................................................................
mom7_2                                                                                                                                                                                                                                          .........................................................................................................................................
mom7_2                                                                                                                                                                                                                                          .........................................................................................................................................
mom7_2                                                                                                                                                                                                                                          .........................................................................................................................................

mom7 consensus
                  TTCTTTCATGGGGAAGCAGATTTGGGTACCACCCAAGTATTGACTCACCCATCAACAACCGCTATGTATTTCGTACATTACTGCCAGCCACCATGAATATTGTACGGTACCATAAATACTTGACCACCTGTAGTACATAAAAACCCAATCCACATCAAAACCCCCTCCCCATGCTTACAAGCAAGTACAGCAATCAACCCTCAACTATCACACATCAACTGCAACTCCAAAGCCACCCCTCACCCACTAGGATACCAACAAACCTACCCACCCTTAACAGTACATAGTACATAAAGCCATTTACCGTACATAGCACATTACAGTCAAATCCCTTCTCGTCCCCATGGATGACCCCCCTCAG
```

```
                    1111111111111111111111111111111111111111111111111111111111111111111111111111111111111111111111111111111111111111111111111111111111111111111111111111111111111111111111111111111111111111111111111111111111111111111111111111111111111111111111111111111111111111111111111111111111111111111111111111111111111111111111111111111111111111111111111111111111111111111
                    6666666666666666666666666666666666666666666666666666666666666666666666666666666666666666666666666666666666666666666666666666666666666666666666666666666666666666666666666666666666666666666666666666666666666666666666666666666666666666666666666666666666666666666666666666666666666666666666666666666666666666666666666666666666666666666666666666666666666666666
                    0000000000000000000000000000000000000000000000000000000000000000000000000000111111111111111111111111111111111111111111111111111111111111111111111111111111111111111111111111111122222222222222222222222222222222222222222222222222222222222222222222222222222222222222222222222222223333333333333333333333333333333333333333333333333333333333333333333333333333333333333
                    2222223333333333444444444455555555556666666666777777777788888888889999999999000000000011111111112222222222333333333344444444445555555555666666666677777777778888888888999999999900000000001111111111222222222233333333334444444444555555555566666666667777777777888888888899999999990000000000111111111122222222223333333333444444444455555555556666666666777777777788888
                    4567890123456789012345678901234567890123456789012345678901234567890123456789012345678901234567890123456789012345678901234567890123456789012345678901234567890123456789012345678901234567890123456789012345678901234567890123456789012345678901234567890123456789012345678901234567890123456789012345678901234567890123456789012345678901234567890123456789012345678901234

CRS                 TTCTTTCATGGGGAAGCAGATTTGGGTACCACCCAAGTATTGACTCACCCATCAACAACCGCTATGTATTTCGTACATTACTGCCAGCCACCATGAATATTGTACGGTACCATAAATACTTGACCACCTGTAGTACATAAAAACCCAATCCACATCAAAACCCCCTCCCCATGCTTACAAGCAAGTACAGCAATCAACCCTCAACTATCACACATCAACTGCAACTCCAAAGCCACCCCTCACCCACTAGGATACCAACAAACCTACCCACCCTTAACAGTACATAGTACATAAAGCCATTTACCGTACATAGCACATTACAGTCAAATCCCTTCTCGTCCCCATGGATGACCCCCCTCAG

mom9-1_1            .....................................................................C................................C.....ACCATAAATACTTGACCACCTGTAG
mom9-1_1            .....................................................................C................................C.....
mom9-1_1            .....................................................................C................................C.....
mom9-1_1            .....................................................................C................................C.....
mom9-1_1            .....................................................................C................................C.....
mom9-1_1            .....................................................................C................................C.....
mom9-1_1            .....................................................................C................................C.....
mom9-1_1            .....................................................................C................................C.....
mom9-1_2            .....................................................................C................................C.....
mom9-1_2            .....................................................................C................................C.....
mom9-1_2            .....................................................................C................................C.....
mom9-1_2            .....................................................................C................................C.....
mom9-1_2            .....................................................................C................................C.....
mom9-1_2            .....................................................................C................................C.....
mom9-1_2            .....................................................................C................................C.....
mom9-1_2            .....................................................................C................................C.....
mom9-1_1                                                                       CGCTATGTATTTCGTACATTACTGC..................C..........................A...........................................................................................................CCTCACCCACTAGGATACCA
mom9-1_1                                                                                            ..................C..........................A...........................................................................................................
mom9-1_1                                                                                            ..................C..........................A...........................................................................................................
mom9-1_1                                                                                            ..................C..........................A...........................................................................................................
mom9-1_1                                                                                            ..................C..........................A...........................................................................................................
mom9-1_1                                                                                            ..................C..........................A...........................................................................................................
mom9-1_1                                                                                            ..................C..........................A...........................................................................................................
mom9-1_1                                                                                            ..................C..........................A...........................................................................................................
mom9-1_2                                                                                            ..................C..........................A...........................................................................................................
mom9-1_2                                                                                            ..................C..........................A...........................................................................................................
mom9-1_2                                                                                            ..................C..........................A...........................................................................................................
mom9-1_2                                                                                            ..................C..........................A...........................................................................................................
mom9-1_2                                                                                            ..................C..........................A...........................................................................................................
mom9-1_2                                                                                            ..................C..........................A...........................................................................................................
mom9-1_2                                                                                            ..................C..........................A...........................................................................................................
mom9-1_2                                                                                            ..................C..........................A...........................................................................................................
mom9-1_1                                                                                                                                                                                                                     CAACTATCACACATCAACTGCAA.........................................................................................................................................
mom9-1_1                                                                                                                                                                                                                                            .........................................................................................................................................
mom9-1_1                                                                                                                                                                                                                                            .........................................................................................................................................
mom9-1_1                                                                                                                                                                                                                                            .........................................................................................................................................
mom9-1_1                                                                                                                                                                                                                                            .........................................................................................................................................
mom9-1_1                                                                                                                                                                                                                                            .........................................................................................................................................
mom9-1_1                                                                                                                                                                                                                                            .........................................................................................................................................
mom9-1_1                                                                                                                                                                                                                                            .........................................................................................................................................
mom9-1_2                                                                                                                                                                                                                                            .........................................................................................................................................
mom9-1_2                                                                                                                                                                                                                                            .........................................................................................................................................
mom9-1_2                                                                                                                                                                                                                                            .........................................................................................................................................
mom9-1_2                                                                                                                                                                                                                                            .........................................................................................................................................
mom9-1_2                                                                                                                                                                                                                                            .........................................................................................................................................
mom9-1_2                                                                                                                                                                                                                                            .........................................................................................................................................
mom9-1_2                                                                                                                                                                                                                                            .........................................................................................................................................
mom9-1_2                                                                                                                                                                                                                                            .........................................................................................................................................

mom9-1 consensus
                    TTCTTTCATGGGGAAGCAGATTTGGGTACCACCCAAGTATTGACTCACCCATCAACAACCGCTATGTATCTCGTACATTACTGCCAGCCACCATGAATATTGCACGGTACCATAAATACTTGACCACCTATAGTACATAAAAACCCAATCCACATCAAAACCCCCTCCCCATGCTTACAAGCAAGTACAGCAATCAACCCTCAACTATCACACATCAACTGCAACTCCAAAGCCACCCCTCACCCACTAGGATACCAACAAACCTACCCACCCTTAACAGTACATAGTACATAAAGCCATTTACCGTACATAGCACATTACAGTCAAATCCCTTCTCGTCCCCATGGATGACCCCCCTCAG
```

```
                    1111111111111111111111111111111111111111111111111111111111111111111111111111111111111111111111111111111111111111111111111111111111111111111111111111111111111111111111111111111111111111111111111111111111111111111111111111111111111111111111111111111111111111111111111111111111111111111111111111111111111111111111111111111111111111111111111111111111111111111
                    6666666666666666666666666666666666666666666666666666666666666666666666666666666666666666666666666666666666666666666666666666666666666666666666666666666666666666666666666666666666666666666666666666666666666666666666666666666666666666666666666666666666666666666666666666666666666666666666666666666666666666666666666666666666666666666666666666666666666666666
                    0000000000000000000000000000000000000000000000000000000000000000000000000000111111111111111111111111111111111111111111111111111111111111111111111111111111111111111111111111111122222222222222222222222222222222222222222222222222222222222222222222222222222222222222222222222222223333333333333333333333333333333333333333333333333333333333333333333333333333333333333
                    2222223333333333444444444455555555556666666666777777777788888888889999999999000000000011111111112222222222333333333344444444445555555555666666666677777777778888888888999999999900000000001111111111222222222233333333334444444444555555555566666666667777777777888888888899999999990000000000111111111122222222223333333333444444444455555555556666666666777777777788888
                    4567890123456789012345678901234567890123456789012345678901234567890123456789012345678901234567890123456789012345678901234567890123456789012345678901234567890123456789012345678901234567890123456789012345678901234567890123456789012345678901234567890123456789012345678901234567890123456789012345678901234567890123456789012345678901234567890123456789012345678901234

CRS                 TTCTTTCATGGGGAAGCAGATTTGGGTACCACCCAAGTATTGACTCACCCATCAACAACCGCTATGTATTTCGTACATTACTGCCAGCCACCATGAATATTGTACGGTACCATAAATACTTGACCACCTGTAGTACATAAAAACCCAATCCACATCAAAACCCCCTCCCCATGCTTACAAGCAAGTACAGCAATCAACCCTCAACTATCACACATCAACTGCAACTCCAAAGCCACCCCTCACCCACTAGGATACCAACAAACCTACCCACCCTTAACAGTACATAGTACATAAAGCCATTTACCGTACATAGCACATTACAGTCAAATCCCTTCTCGTCCCCATGGATGACCCCCCTCAG

mom16_1             ............................................................................................................ACCATAAATACTTGACCACCTGTAG
mom16_1             ............................................................................................................
mom16_1             ............................................................................................................
mom16_1             ............................................................................................................
mom16_1             ............................................................................................................
mom16_1             ............................................................................................................
mom16_1             ............................................................................................................
mom16_1             ............................................................................................................
mom16_2             ............................................................................................................
mom16_2             ............................................................................................................
mom16_2             ............................................................................................................
mom16_2             ............................................................................................................
mom16_2             ............................................................................................................
mom16_2             ............................................................................................................
mom16_2             ............................................................................................................
mom16_2             ............................................................................................................
mom16_1                                                                        CGCTATGTATTTCGTACATTACTGC.........................................................................................................................................................CCTCACCCACTAGGATACCA
mom16_1                                                                                             .........................................................................................................................................................
mom16_1                                                                                             .........................................................................................................................................................
mom16_1                                                                                             .........................................................................................................................................................
mom16_1                                                                                             .........................................................................................................................................................
mom16_1                                                                                             .........................................................................................................................................................
mom16_1                                                                                             .........................................................................................................................................................
mom16_1                                                                                             .........................................................................................................................................................
mom16_2                                                                                             .........................................................................................................................................................
mom16_2                                                                                             .........................................................................................................................................................
mom16_2                                                                                             .........................................................................................................................................................
mom16_2                                                                                             .........................................................................................................................................................
mom16_2                                                                                             .........................................................................................................................................................
mom16_2                                                                                             .........................................................................................................................................................
mom16_2                                                                                             .........................................................................................................................................................
mom16_2                                                                                             .........................................................................................................................................................
mom16_1                                                                                                                                                                                                                     CAACTATCACACATCAACTGCAA.............................................G.................C.........................................................................
mom16_1                                                                                                                                                                                                                                            .............................................G.................C.........................................................................
mom16_1                                                                                                                                                                                                                                            .............................................G.................C.........................................................................
mom16_1                                                                                                                                                                                                                                            .............................................G.................C.........................................................................
mom16_1                                                                                                                                                                                                                                            .............................................G.................C.........................................................................
mom16_1                                                                                                                                                                                                                                            .............................................G.................C.........................................................................
mom16_1                                                                                                                                                                                                                                            .............................................G.................C.........................................................................
mom16_1                                                                                                                                                                                                                                            .............................................G.................C.........................................................................
mom16_2                                                                                                                                                                                                                                            .............................................G.................C.........................................................................
mom16_2                                                                                                                                                                                                                                            .............................................G.................C.........................................................................
mom16_2                                                                                                                                                                                                                                            .............................................G.................C.........................................................................
mom16_2                                                                                                                                                                                                                                            .............................................G.................C.........................................................................
mom16_2                                                                                                                                                                                                                                            .............................................G.................C.........................................................................
mom16_2                                                                                                                                                                                                                                            .............................................G.................C.........................................................................
mom16_2                                                                                                                                                                                                                                            .............................................G.................C.........................................................................
mom16_2                                                                                                                                                                                                                                            .............................................G.................C.........................................................................

mom16 consensus
                    TTCTTTCATGGGGAAGCAGATTTGGGTACCACCCAAGTATTGACTCACCCATCAACAACCGCTATGTATTTCGTACATTACTGCCAGCCACCATGAATATTGTACGGTACCATAAATACTTGACCACCTGTAGTACATAAAAACCCAATCCACATCAAAACCCCCTCCCCATGCTTACAAGCAAGTACAGCAATCAACCCTCAACTATCACACATCAACTGCAACTCCAAAGCCACCCCTCACCCACTAGGATACCAACAAACCTACCCGCCCTTAACAGTACATAGCACATAAAGCCATTTACCGTACATAGCACATTACAGTCAAATCCCTTCTCGTCCCCATGGATGACCCCCCTCAG
```

```
              1111111111111111111111111111111111111111111111111111111111111111111111111111111111111111111111111111111111111111111111111111111111111111111111111111111111111111111111111111111111111111111111111111111111111111111111111111111111111111111111111111111111111111111111111111111111111111111111111111111111111111111111111111111111111111111111111111111111111111111111111
              6666666666666666666666666666666666666666666666666666666666666666666666666666666666666666666666666666666666666666666666666666666666666666666666666666666666666666666666666666666666666666666666666666666666666666666666666666666666666666666666666666666666666666666666666666666666666666666666666666666666666666666666666666666666666666666666666666666666666666666666666
              0000000000000000000000000000000000000000000000000000000000000000000000000000111111111111111111111111111111111111111111111111111111111111111111111111111111111111111111111111111122222222222222222222222222222222222222222222222222222222222222222222222222222222222222222222222222223333333333333333333333333333333333333333333333333333333333333333333333333333333333333
              2222223333333333444444444455555555556666666666777777777788888888889999999999000000000011111111112222222222333333333344444444445555555555666666666677777777778888888888999999999900000000001111111111222222222233333333334444444444555555555566666666667777777777888888888899999999990000000000111111111122222222223333333333444444444455555555556666666666777777777788888
              4567890123456789012345678901234567890123456789012345678901234567890123456789012345678901234567890123456789012345678901234567890123456789012345678901234567890123456789012345678901234567890123456789012345678901234567890123456789012345678901234567890123456789012345678901234567890123456789012345678901234567890123456789012345678901234567890123456789012345678901234

CRS           TTCTTTCATGGGGAAGCAGATTTGGGTACCACCCAAGTATTGACTCACCCATCAACAACCGCTATGTATTTCGTACATTACTGCCAGCCACCATGAATATTGTACGGTACCATAAATACTTGACCACCTGTAGTACATAAAAACCCAATCCACATCAAAACCCCCTCCCCATGCTTACAAGCAAGTACAGCAATCAACCCTCAACTATCACACATCAACTGCAACTCCAAAGCCACCCCTCACCCACTAGGATACCAACAAACCTACCCACCCTTAACAGTACATAGTACATAAAGCCATTTACCGTACATAGCACATTACAGTCAAATCCCTTCTCGTCCCCATGGATGACCCCCCTCAG

mom20_1       .............................................T........................................................C.... ACCATAAATACTTGACCACCTGTAG
mom20_1       .............................................T........................................................C....
mom20_1       .............................................T........................................................C....
mom20_1       .............................................T........................................................C....
mom20_1       .............................................T........................................................C....
mom20_1       .............................................T........................................................C....
mom20_1       .............................................T........................................................C....
mom20_1       .............................................T........................................................C....
mom20_2       .............................................T........................................................C....
mom20_2       .............................................T........................................................C....
mom20_2       .............................................T........................................................C....
mom20_2       .............................................T........................................................C....
mom20_2       .............................................T........................................................C....
mom20_2       .............................................T........................................................C....
mom20_2       .............................................T........................................................C....
mom20_2       .............................................T........................................................C....
mom20_1                                                                  CGCTATGTATTTCGTACATTACTGC..................C........................................................................................................C.............................CCTCACCCACTAGGATACCA
mom20_1                                                                                          ..................C........................................................................................................C.............................
mom20_1                                                                                          ..................C........................................................................................................C.............................
mom20_1                                                                                          ..................C........................................................................................................C.............................
mom20_1                                                                                          ..................C........................................................................................................C.............................
mom20_1                                                                                          ..................C........................................................................................................C.............................
mom20_1                                                                                          ..................C........................................................................................................C.............................
mom20_1                                                                                          ..................C........................................................................................................C.............................
mom20_2                                                                                          ..................C........................................................................................................C.............................
mom20_2                                                                                          ..................C........................................................................................................C.............................
mom20_2                                                                                          ..................C........................................................................................................C.............................
mom20_2                                                                                          ..................C........................................................................................................C.............................
mom20_2                                                                                          ..................C........................................................................................................C.............................
mom20_2                                                                                          ..................C........................................................................................................C.............................
mom20_2                                                                                          ..................C........................................................................................................C.............................
mom20_2                                                                                          ..................C........................................................................................................C.............................
mom20_1                                                                                                                                                                                                                       CAACTATCACACATCAACTGCAA...............................................................C.........................................................................
mom20_1                                                                                                                                                                                                                                              ...............................................................C.........................................................................
mom20_1                                                                                                                                                                                                                                              ...............................................................C.........................................................................
mom20_1                                                                                                                                                                                                                                              ...............................................................C.........................................................................
mom20_1                                                                                                                                                                                                                                              ...............................................................C.........................................................................
mom20_1                                                                                                                                                                                                                                              ...............................................................C.........................................................................
mom20_1                                                                                                                                                                                                                                              ...............................................................C.........................................................................
mom20_1                                                                                                                                                                                                                                              ...............................................................C.........................................................................
mom20_2                                                                                                                                                                                                                                              ...............................................................C.........................................................................
mom20_2                                                                                                                                                                                                                                              ...............................................................C.........................................................................
mom20_2                                                                                                                                                                                                                                              ...............................................................C.........................................................................
mom20_2                                                                                                                                                                                                                                              ...............................................................C.........................................................................
mom20_2                                                                                                                                                                                                                                              ...............................................................C.........................................................................
mom20_2                                                                                                                                                                                                                                              ...............................................................C.........................................................................
mom20_2                                                                                                                                                                                                                                              ...............................................................C.........................................................................
mom20_2                                                                                                                                                                                                                                              ...............................................................C.........................................................................
mom20_3                                          ............T........................................................C...........................................................................................
mom20_3                                          ............................G..........A.............................................................................................................G...........
mom20_3                                          .....................................................................................................................................................G...........
mom20_3                                          ............T........................................................C...........................................................................................
mom20_3                                          ............T.........................T........A.....................C........................TC.................................................................

mom20 consensus
              TTCTTTCATGGGGAAGCAGATTTGGGTACCACCCAAGTATTGACTTACCCATCAACAACCGCTATGTATTTCGTACATTACTGCCAGCCACCATGAATATTGCACGGTACCATAAATACTTGACCACCTGTAGTACATAAAAACCCAATCCACATCAAAACCCCCTCCCCATGCTTACAAGCAAGTACAGCAATCAACCCTCAACTACCACACATCAACTGCAACTCCAAAGCCACCCCTCACCCACTAGGATACCAACAAACCTACCCACCCTTAACAGTACATAGCACATAAAGCCATTTACCGTACATAGCACATTACAGTCAAATCCCTTCTCGTCCCCATGGATGACCCCCCTCAG


              1111111111111111111111111111111111111111111111111111111111111111111111111111111111111111111111111111111111111111111111111111111111111111111111111111111111111111111111111111111111111111111111111111111111111111111111111111111111111111111111111111111111111111111111111111111111111111111111111111111111111111111111111111111111111111111111111111111111111111111111111
              6666666666666666666666666666666666666666666666666666666666666666666666666666666666666666666666666666666666666666666666666666666666666666666666666666666666666666666666666666666666666666666666666666666666666666666666666666666666666666666666666666666666666666666666666666666666666666666666666666666666666666666666666666666666666666666666666666666666666666666666666
              0000000000000000000000000000000000000000000000000000000000000000000000000000111111111111111111111111111111111111111111111111111111111111111111111111111111111111111111111111111122222222222222222222222222222222222222222222222222222222222222222222222222222222222222222222222222223333333333333333333333333333333333333333333333333333333333333333333333333333333333333
              2222223333333333444444444455555555556666666666777777777788888888889999999999000000000011111111112222222222333333333344444444445555555555666666666677777777778888888888999999999900000000001111111111222222222233333333334444444444555555555566666666667777777777888888888899999999990000000000111111111122222222223333333333444444444455555555556666666666777777777788888
              4567890123456789012345678901234567890123456789012345678901234567890123456789012345678901234567890123456789012345678901234567890123456789012345678901234567890123456789012345678901234567890123456789012345678901234567890123456789012345678901234567890123456789012345678901234567890123456789012345678901234567890123456789012345678901234567890123456789012345678901234

CRS           TTCTTTCATGGGGAAGCAGATTTGGGTACCACCCAAGTATTGACTCACCCATCAACAACCGCTATGTATTTCGTACATTACTGCCAGCCACCATGAATATTGTACGGTACCATAAATACTTGACCACCTGTAGTACATAAAAACCCAATCCACATCAAAACCCCCTCCCCATGCTTACAAGCAAGTACAGCAATCAACCCTCAACTATCACACATCAACTGCAACTCCAAAGCCACCCCTCACCCACTAGGATACCAACAAACCTACCCACCCTTAACAGTACATAGTACATAAAGCCATTTACCGTACATAGCACATTACAGTCAAATCCCTTCTCGTCCCCATGGATGACCCCCCTCAG

mom21-1_1     .............................................T........................................................C.... ACCATAAATACTTGACCACCTGTAG
mom21-1_1     .............................................T........................................................C....
mom21-1_1     .............................................T........................................................C....
mom21-1_1     .............................................T........................................................C....
mom21-1_1     .............................................T........................................................C....
mom21-1_1     .............................................T........................................................C....
mom21-1_1     .............................................T........................................................C....
mom21-1_1     .............................................T........................................................C....
mom21-1_2     .............................................T........................................................C....
mom21-1_2     .............................................T........................................................C....
mom21-1_2     .............................................T........................................................C....
mom21-1_2     .............................................T........................................................C....
mom21-1_2     .............................................T........................................................C....
mom21-1_2     .............................................T........................................................C....
mom21-1_2     .............................................T........................................................C....
mom21-1_2     .............................................T........................................................C....
mom21-1_1                                                                CGCTATGTATTTCGTACATTACTGC..................C......................................................................................................................................CCTCACCCACTAGGATACCA
mom21-1_1                                                                                        ..................C......................................................................................................................................
mom21-1_1                                                                                        ..................C......................................................................................................................................
mom21-1_1                                                                                        ..................C......................................................................................................................................
mom21-1_1                                                                                        ..................C......................................................................................................................................
mom21-1_1                                                                                        ..................C......................................................................................................................................
mom21-1_1                                                                                        ..................C......................................................................................................................................
mom21-1_1                                                                                        ..................C......................................................................................................................................
mom21-1_2                                                                                        ..................C......................................................................................................................................
mom21-1_2                                                                                        ..................C......................................................................................................................................
mom21-1_2                                                                                        ..................C......................................................................................................................................
mom21-1_2                                                                                        ..................C......................................................................................................................................
mom21-1_2                                                                                        ..................C......................................................................................................................................
mom21-1_2                                                                                        ..................C......................................................................................................................................
mom21-1_2                                                                                        ..................C......................................................................................................................................
mom21-1_2                                                                                        ..................C......................................................................................................................................
mom21-1_1                                                                                                                                                                                                                      CAACTATCACACATCAACTGCAA.........................................................................................................................................
mom21-1_1                                                                                                                                                                                                                                             .........................................................................................................................................
mom21-1_1                                                                                                                                                                                                                                             .........................................................................................................................................
mom21-1_1                                                                                                                                                                                                                                             .........................................................................................................................................
mom21-1_1                                                                                                                                                                                                                                             .........................................................................................................................................
mom21-1_1                                                                                                                                                                                                                                             .........................................................................................................................................
mom21-1_1                                                                                                                                                                                                                                             .........................................................................................................................................
mom21-1_1                                                                                                                                                                                                                                             .........................................................................................................................................
mom21-1_2                                                                                                                                                                                                                                             .........................................................................................................................................
mom21-1_2                                                                                                                                                                                                                                             .........................................................................................................................................
mom21-1_2                                                                                                                                                                                                                                             .........................................................................................................................................
mom21-1_2                                                                                                                                                                                                                                             .........................................................................................................................................
mom21-1_2                                                                                                                                                                                                                                             .........................................................................................................................................
mom21-1_2                                                                                                                                                                                                                                             .........................................................................................................................................
mom21-1_2                                                                                                                                                                                                                                             .........................................................................................................................................
mom21-1_2                                                                                                                                                                                                                                             .........................................................................................................................................

mom21-1 consensus
              TTCTTTCATGGGGAAGCAGATTTGGGTACCACCCAAGTATTGACTTACCCATCAACAACCGCTATGTATTTCGTACATTACTGCCAGCCACCATGAATATTGCACGGTACCATAAATACTTGACCACCTGTAGTACATAAAAACCCAATCCACATCAAAACCCCCTCCCCATGCTTACAAGCAAGTACAGCAATCAACCCTCAACTATCACACATCAACTGCAACTCCAAAGCCACCCCTCACCCACTAGGATACCAACAAACCTACCCACCCTTAACAGTACATAGTACATAAAGCCATTTACCGTACATAGCACATTACAGTCAAATCCCTTCTCGTCCCCATGGATGACCCCCCTCAG
```

```
                1111111111111111111111111111111111111111111111111111111111111111111111111111111111111111111111111111111111111111111111111111111111111111111111111111111111111111111111111111111111111111111111111111111111111111111111111111111111111111111111111111111111111111111111111111111111111111111111111111111111111111111111111111111111111111111111111111111111111111111111
                6666666666666666666666666666666666666666666666666666666666666666666666666666666666666666666666666666666666666666666666666666666666666666666666666666666666666666666666666666666666666666666666666666666666666666666666666666666666666666666666666666666666666666666666666666666666666666666666666666666666666666666666666666666666666666666666666666666666666666666666
                0000000000000000000000000000000000000000000000000000000000000000000000000000111111111111111111111111111111111111111111111111111111111111111111111111111111111111111111111111111122222222222222222222222222222222222222222222222222222222222222222222222222222222222222222222222222223333333333333333333333333333333333333333333333333333333333333333333333333333333333333
                2222223333333333444444444455555555556666666666777777777788888888889999999999000000000011111111112222222222333333333344444444445555555555666666666677777777778888888888999999999900000000001111111111222222222233333333334444444444555555555566666666667777777777888888888899999999990000000000111111111122222222223333333333444444444455555555556666666666777777777788888
                4567890123456789012345678901234567890123456789012345678901234567890123456789012345678901234567890123456789012345678901234567890123456789012345678901234567890123456789012345678901234567890123456789012345678901234567890123456789012345678901234567890123456789012345678901234567890123456789012345678901234567890123456789012345678901234567890123456789012345678901234

CRS             TTCTTTCATGGGGAAGCAGATTTGGGTACCACCCAAGTATTGACTCACCCATCAACAACCGCTATGTATTTCGTACATTACTGCCAGCCACCATGAATATTGTACGGTACCATAAATACTTGACCACCTGTAGTACATAAAAACCCAATCCACATCAAAACCCCCTCCCCATGCTTACAAGCAAGTACAGCAATCAACCCTCAACTATCACACATCAACTGCAACTCCAAAGCCACCCCTCACCCACTAGGATACCAACAAACCTACCCACCCTTAACAGTACATAGTACATAAAGCCATTTACCGTACATAGCACATTACAGTCAAATCCCTTCTCGTCCCCATGGATGACCCCCCTCAG

lonI4_1         ............................................................................................................ACCATAAATACTTGACCACCTGTAG
lonI4_1         ............................................................................................................
lonI4_1         ............................................................................................................
lonI4_1         ............................................................................................................
lonI4_1         ............................................................................................................
lonI4_1         ............................................................................................................
lonI4_1         ............................................................................................................
lonI4_1         ............................................................................................................
lonI4_2         ............................................................................................................
lonI4_2         ............................................................................................................
lonI4_2         ............................................................................................................
lonI4_2         ............................................................................................................
lonI4_2         ............................................................................................................
lonI4_2         ............................................................................................................
lonI4_2         ............................................................................................................
lonI4_2         ............................................................................................................
lonI4_1                                                                    CGCTATGTATTTCGTACATTACTGC.....................................A.............................................................................T.....................................CCTCACCCACTAGGATACCA
lonI4_1                                                                                             .....................................A.............................................................................T.....................................
lonI4_1                                                                                             .....................................A.............................................................................T.....................................
lonI4_1                                                                                             .....................................A.............................................................................T.....................................
lonI4_1                                                                                             .....................................A.............................................................................T.....................................
lonI4_1                                                                                             .....................................A.............................................................................T.....................................
lonI4_1                                                                                             .....................................A.............................................................................T.....................................
lonI4_1                                                                                             .....................................A.............................................................................T.....................................
lonI4_2                                                                                             .....................................A.............................................................................T.....................................
lonI4_2                                                                                             .....................................A.............................................................................T.....................................
lonI4_2                                                                                             .....................................A.............................................................................T.....................................
lonI4_2                                                                                             .....................................A.............................................................................T.....................................
lonI4_2                                                                                             .....................................A.............................................................................T.....................................
lonI4_2                                                                                             .....................................A.............................................................................T.....................................
lonI4_2                                                                                             .....................................A.............................................................................T.....................................
lonI4_2                                                                                             .....................................A.............................................................................T.....................................
lonI4_1                                                                                                                                                                                                                         CAACTATCACACATCAACTGCAA.............................................................................C....................................C......................
lonI4_1                                                                                                                                                                                                                                                .............................................................................C....................................C......................
lonI4_1                                                                                                                                                                                                                                                .............................................................................C....................................C......................
lonI4_1                                                                                                                                                                                                                                                .............................................................................C....................................C......................
lonI4_1                                                                                                                                                                                                                                                .............................................................................C....................................C......................
lonI4_1                                                                                                                                                                                                                                                .............................................................................C....................................C......................
lonI4_1                                                                                                                                                                                                                                                .............................................................................C....................................C......................
lonI4_1                                                                                                                                                                                                                                                .............................................................................C....................................C......................
lonI4_2                                                                                                                                                                                                                                                .............................................................................C....................................C......................
lonI4_2                                                                                                                                                                                                                                                .............................................................................C....................................C......................
lonI4_2                                                                                                                                                                                                                                                .............................................................................C....................................C......................
lonI4_2                                                                                                                                                                                                                                                .............................................................................C....................................C......................
lonI4_2                                                                                                                                                                                                                                                .............................................................................C....................................C......................
lonI4_2                                                                                                                                                                                                                                                .............................................................................T....................................C......................
lonI4_2                                                                                                                                                                                                                                                .............................................................................C....................................C......................
lonI4_2                                                                                                                                                                                                                                                .............................................................................C....................................C......................

lonI4 consensus
                TTCTTTCATGGGGAAGCAGATTTGGGTACCACCCAAGTATTGACTCACCCATCAACAACCGCTATGTATTTCGTACATTACTGCCAGCCACCATGAATATTGTACGGTACCATAAATACTTAACCACCTGTAGTACATAAAAACCCAATCCACATCAAAACCCCCTCCCCATGCTTACAAGCAAGTACAGCAATCAACCTTCAACTATCACACATCAACTGCAACTCCAAAGCCACCCCTCACCCACTAGGATACCAACAAACCTACCCACCCTTAACAGTACATAGTACATAAAGCCATTCACCGTACATAGCACATTACAGTCAAATCCCTTCTCGCCCCCATGGATGACCCCCCTCAG
```

```
                1111111111111111111111111111111111111111111111111111111111111111111111111111111111111111111111111111111111111111111111111111111111111111111111111111111111111111111111111111111111111111111111111111111111111111111111111111111111111111111111111111111111111111111111111111111111111111111111111111111111111111111111111111111111111111111111111111111111111111111111
                6666666666666666666666666666666666666666666666666666666666666666666666666666666666666666666666666666666666666666666666666666666666666666666666666666666666666666666666666666666666666666666666666666666666666666666666666666666666666666666666666666666666666666666666666666666666666666666666666666666666666666666666666666666666666666666666666666666666666666666666
                0000000000000000000000000000000000000000000000000000000000000000000000000000111111111111111111111111111111111111111111111111111111111111111111111111111111111111111111111111111122222222222222222222222222222222222222222222222222222222222222222222222222222222222222222222222222223333333333333333333333333333333333333333333333333333333333333333333333333333333333333
                2222223333333333444444444455555555556666666666777777777788888888889999999999000000000011111111112222222222333333333344444444445555555555666666666677777777778888888888999999999900000000001111111111222222222233333333334444444444555555555566666666667777777777888888888899999999990000000000111111111122222222223333333333444444444455555555556666666666777777777788888
                4567890123456789012345678901234567890123456789012345678901234567890123456789012345678901234567890123456789012345678901234567890123456789012345678901234567890123456789012345678901234567890123456789012345678901234567890123456789012345678901234567890123456789012345678901234567890123456789012345678901234567890123456789012345678901234567890123456789012345678901234

CRS             TTCTTTCATGGGGAAGCAGATTTGGGTACCACCCAAGTATTGACTCACCCATCAACAACCGCTATGTATTTCGTACATTACTGCCAGCCACCATGAATATTGTACGGTACCATAAATACTTGACCACCTGTAGTACATAAAAACCCAATCCACATCAAAACCCCCTCCCCATGCTTACAAGCAAGTACAGCAATCAACCCTCAACTATCACACATCAACTGCAACTCCAAAGCCACCCCTCACCCACTAGGATACCAACAAACCTACCCACCCTTAACAGTACATAGTACATAAAGCCATTTACCGTACATAGCACATTACAGTCAAATCCCTTCTCGTCCCCATGGATGACCCCCCTCAG

lonI41_1        ............................................................................................................ACCATAAATACTTGACCACCTGTAG
lonI41_1        ............................................................................................................
lonI41_1        ............................................................................................................
lonI41_1        ............................................................................................................
lonI41_1        ............................................................................................................
lonI41_1        ............................................................................................................
lonI41_1        ............................................................................................................
lonI41_1        ............................................................................................................
lonI41_2        ............................................................................................................
lonI41_2        ............................................................................................................
lonI41_2        ............................................................................................................
lonI41_2        ............................................................................................................
lonI41_2        ............................................................................................................
lonI41_2        ............................................................................................................
lonI41_2        ............................................................................................................
lonI41_2        ............................................................................................................
lonI41_1                                                                   CGCTATGTATTTCGTACATTACTGC...............................................................................................................................G.........................CCTCACCCACTAGGATACCA
lonI41_1                                                                                            ...............................................................................................................................G.........................
lonI41_1                                                                                            ...............................................................................................................................G.........................
lonI41_1                                                                                            ...............................................................................................................................G.........................
lonI41_1                                                                                            ...............................................................................................................................G.........................
lonI41_1                                                                                            ...............................................................................................................................G.........................
lonI41_1                                                                                            ...............................................................................................................................G.........................
lonI41_1                                                                                            ...............................................................................................................................G.........................
lonI41_2                                                                                            ...............................................................................................................................G.........................
lonI41_2                                                                                            ...............................................................................................................................G.........................
lonI41_2                                                                                            ...............................................................................................................................G.........................
lonI41_2                                                                                            ...............................................................................................................................G.........................
lonI41_2                                                                                            ...............................................................................................................................G.........................
lonI41_2                                                                                            ...............................................................................................................................G.........................
lonI41_2                                                                                            ...............................................................................................................................G.........................
lonI41_2                                                                                            ...............................................................................................................................G.........................
lonI41_1                                                                                                                                                                                                                        CAACTATCACACATCAACTGCAA.........................................................................................................................................
lonI41_1                                                                                                                                                                                                                                               .........................................................................................................................................
lonI41_1                                                                                                                                                                                                                                               .........................................................................................................................................
lonI41_1                                                                                                                                                                                                                                               .........................................................................................................................................
lonI41_1                                                                                                                                                                                                                                               .........................................................................................................................................
lonI41_1                                                                                                                                                                                                                                               .........................................................................................................................................
lonI41_1                                                                                                                                                                                                                                               .........................................................................................................................................
lonI41_1                                                                                                                                                                                                                                               .........................................................................................................................................
lonI41_2                                                                                                                                                                                                                                               .........................................................................................................................................
lonI41_2                                                                                                                                                                                                                                               .........................................................................................................................................
lonI41_2                                                                                                                                                                                                                                               .........................................................................................................................................
lonI41_2                                                                                                                                                                                                                                               .........................................................................................................................................
lonI41_2                                                                                                                                                                                                                                               .........................................................................................................................................
lonI41_2                                                                                                                                                                                                                                               .........................................................................................................................................
lonI41_2                                                                                                                                                                                                                                               .........................................................................................................................................
lonI41_2                                                                                                                                                                                                                                               .........................................................................................................................................

lonI41 consensus
                TTCTTTCATGGGGAAGCAGATTTGGGTACCACCCAAGTATTGACTCACCCATCAACAACCGCTATGTATTTCGTACATTACTGCCAGCCACCATGAATATTGTACGGTACCATAAATACTTGACCACCTGTAGTACATAAAAACCCAATCCACATCAAAACCCCCTCCCCATGCTTACAAGCAAGTACAGCAATCAACCCTCAACTATCACGCATCAACTGCAACTCCAAAGCCACCCCTCACCCACTAGGATACCAACAAACCTACCCACCCTTAACAGTACATAGTACATAAAGCCATTTACCGTACATAGCACATTACAGTCAAATCCCTTCTCGTCCCCATGGATGACCCCCCTCAG
```

```
                 1111111111111111111111111111111111111111111111111111111111111111111111111111111111111111111111111111111111111111111111111111111111111111111111111111111111111111111111111111111111111111111111111111111111111111111111111111111111111111111111111111111111111111111111111111111111111111111111111111111111111111111111111111111111111111111111111111111111111111111
                 6666666666666666666666666666666666666666666666666666666666666666666666666666666666666666666666666666666666666666666666666666666666666666666666666666666666666666666666666666666666666666666666666666666666666666666666666666666666666666666666666666666666666666666666666666666666666666666666666666666666666666666666666666666666666666666666666666666666666666666
                 0000000000000000000000000000000000000000000000000000000000000000000000000000111111111111111111111111111111111111111111111111111111111111111111111111111111111111111111111111111122222222222222222222222222222222222222222222222222222222222222222222222222222222222222222222222222223333333333333333333333333333333333333333333333333333333333333333333333333333333333333
                 2222223333333333444444444455555555556666666666777777777788888888889999999999000000000011111111112222222222333333333344444444445555555555666666666677777777778888888888999999999900000000001111111111222222222233333333334444444444555555555566666666667777777777888888888899999999990000000000111111111122222222223333333333444444444455555555556666666666777777777788888
                 4567890123456789012345678901234567890123456789012345678901234567890123456789012345678901234567890123456789012345678901234567890123456789012345678901234567890123456789012345678901234567890123456789012345678901234567890123456789012345678901234567890123456789012345678901234567890123456789012345678901234567890123456789012345678901234567890123456789012345678901234

CRS              TTCTTTCATGGGGAAGCAGATTTGGGTACCACCCAAGTATTGACTCACCCATCAACAACCGCTATGTATTTCGTACATTACTGCCAGCCACCATGAATATTGTACGGTACCATAAATACTTGACCACCTGTAGTACATAAAAACCCAATCCACATCAAAACCCCCTCCCCATGCTTACAAGCAAGTACAGCAATCAACCCTCAACTATCACACATCAACTGCAACTCCAAAGCCACCCCTCACCCACTAGGATACCAACAAACCTACCCACCCTTAACAGTACATAGTACATAAAGCCATTTACCGTACATAGCACATTACAGTCAAATCCCTTCTCGTCCCCATGGATGACCCCCCTCAG

lonI48_1         ............................................................................................................ACCATAAATACTTGACCACCTGTAG
lonI48_1         ............................................................................................................
lonI48_1         ............................................................................................................
lonI48_1         ............................................................................................................
lonI48_1         ............................................................................................................
lonI48_1         ............................................................................................................
lonI48_1         ............................................................................................................
lonI48_1         ............................................................................................................
lonI48_2         ............................................................................................................
lonI48_2         ............................................................................................................
lonI48_2         ............................................................................................................
lonI48_2         ............................................................................................................
lonI48_2         ............................................................................................................
lonI48_2         ............................................................................................................
lonI48_2         ............................................................................................................
lonI48_2         ............................................................................................................
lonI48_1                                                                    CGCTATGTATTTCGTACATTACTGC.........................................................................................................................................................CCTCACCCACTAGGATACCA
lonI48_1                                                                                             .........................................................................................................................................................
lonI48_1                                                                                             .........................................................................................................................................................
lonI48_1                                                                                             .........................................................................................................................................................
lonI48_1                                                                                             .........................................................................................................................................................
lonI48_1                                                                                             .........................................................................................................................................................
lonI48_1                                                                                             .........................................................................................................................................................
lonI48_1                                                                                             .........................................................................................................................................................
lonI48_2                                                                                             .........................................................................................................................................................
lonI48_2                                                                                             .........................................................................................................................................................
lonI48_2                                                                                             .........................................................................................................................................................
lonI48_2                                                                                             .........................................................................................................................................................
lonI48_2                                                                                             .........................................................................................................................................................
lonI48_2                                                                                             .........................................................................................................................................................
lonI48_2                                                                                             .........................................................................................................................................................
lonI48_2                                                                                             .........................................................................................................................................................
lonI48_1                                                                                                                                                                                                                  CAACTATCACACATCAACTGCAA.............................................G.................C.........................................................................
lonI48_1                                                                                                                                                                                                                                         .............................................G.................C.........................................................................
lonI48_1                                                                                                                                                                                                                                         .............................................G.................C.........................................................................
lonI48_1                                                                                                                                                                                                                                         .............................................G.................C.........................................................................
lonI48_1                                                                                                                                                                                                                                         .............................................G.................C.........................................................................
lonI48_1                                                                                                                                                                                                                                         .............................................G.................C.........................................................................
lonI48_1                                                                                                                                                                                                                                         .............................................G.................C.........................................................................
lonI48_1                                                                                                                                                                                                                                         .............................................G.................C.........................................................................
lonI48_2                                                                                                                                                                                                                                         .............................................G.................C.........................................................................
lonI48_2                                                                                                                                                                                                                                         .............................................G.................C.........................................................................
lonI48_2                                                                                                                                                                                                                                         .............................................G.................C.........................................................................
lonI48_2                                                                                                                                                                                                                                         .............................................G.................C.........................................................................
lonI48_2                                                                                                                                                                                                                                         .............................................G.................C.........................................................................
lonI48_2                                                                                                                                                                                                                                         .............................................G.................C.........................................................................
lonI48_2                                                                                                                                                                                                                                         .............................................G.................C.........................................................................
lonI48_2                                                                                                                                                                                                                                         .............................................G.................C.........................................................................

lonI48 consensus
                 TTCTTTCATGGGGAAGCAGATTTGGGTACCACCCAAGTATTGACTCACCCATCAACAACCGCTATGTATTTCGTACATTACTGCCAGCCACCATGAATATTGTACGGTACCATAAATACTTGACCACCTGTAGTACATAAAAACCCAATCCACATCAAAACCCCCTCCCCATGCTTACAAGCAAGTACAGCAATCAACCCTCAACTATCACACATCAACTGCAACTCCAAAGCCACCCCTCACCCACTAGGATACCAACAAACCTACCCGCCCTTAACAGTACATAGCACATAAAGCCATTTACCGTACATAGCACATTACAGTCAAATCCCTTCTCGTCCCCATGGATGACCCCCCTCAG


                 1111111111111111111111111111111111111111111111111111111111111111111111111111111111111111111111111111111111111111111111111111111111111111111111111111111111111111111111111111111111111111111111111111111111111111111111111111111111111111111111111111111111111111111111111111111111111111111111111111111111111111111111111111111111111111111111111111111111111111111
                 6666666666666666666666666666666666666666666666666666666666666666666666666666666666666666666666666666666666666666666666666666666666666666666666666666666666666666666666666666666666666666666666666666666666666666666666666666666666666666666666666666666666666666666666666666666666666666666666666666666666666666666666666666666666666666666666666666666666666666666
                 0000000000000000000000000000000000000000000000000000000000000000000000000000111111111111111111111111111111111111111111111111111111111111111111111111111111111111111111111111111122222222222222222222222222222222222222222222222222222222222222222222222222222222222222222222222222223333333333333333333333333333333333333333333333333333333333333333333333333333333333333
                 2222223333333333444444444455555555556666666666777777777788888888889999999999000000000011111111112222222222333333333344444444445555555555666666666677777777778888888888999999999900000000001111111111222222222233333333334444444444555555555566666666667777777777888888888899999999990000000000111111111122222222223333333333444444444455555555556666666666777777777788888
                 4567890123456789012345678901234567890123456789012345678901234567890123456789012345678901234567890123456789012345678901234567890123456789012345678901234567890123456789012345678901234567890123456789012345678901234567890123456789012345678901234567890123456789012345678901234567890123456789012345678901234567890123456789012345678901234567890123456789012345678901234

CRS              TTCTTTCATGGGGAAGCAGATTTGGGTACCACCCAAGTATTGACTCACCCATCAACAACCGCTATGTATTTCGTACATTACTGCCAGCCACCATGAATATTGTACGGTACCATAAATACTTGACCACCTGTAGTACATAAAAACCCAATCCACATCAAAACCCCCTCCCCATGCTTACAAGCAAGTACAGCAATCAACCCTCAACTATCACACATCAACTGCAACTCCAAAGCCACCCCTCACCCACTAGGATACCAACAAACCTACCCACCCTTAACAGTACATAGTACATAAAGCCATTTACCGTACATAGCACATTACAGTCAAATCCCTTCTCGTCCCCATGGATGACCCCCCTCAG

lonI63_1         ......................................................................................................C.....ACCATAAATACTTGACCACCTGTAG
lonI63_1         ......................................................................................................C.....
lonI63_1         ......................................................................................................C.....
lonI63_1         ......................................................................................................C.....
lonI63_1         ......................................................................................................C.....
lonI63_1         ......................................................................................................C.....
lonI63_1         ......................................................................................................C.....
lonI63_1         ......................................................................................................C.....
lonI63_2         ......................................................................................................C.....
lonI63_2         ......................................................................................................C.....
lonI63_2         ......................................................................................................C.....
lonI63_2         ......................................................................................................C.....
lonI63_2         ......................................................................................................C.....
lonI63_2         ......................................................................................................C.....
lonI63_2         ......................................................................................................C.....
lonI63_2         ......................................................................................................C.....
lonI63_1                                                                    CGCTATGTATTTCGTACATTACTGC..................C..........................A.............................C.....C.......................................................................CCTCACCCACTAGGATACCA
lonI63_1                                                                                             ..................C..........................A.............................C.....C.......................................................................
lonI63_1                                                                                             ..................C..........................A.............................C.....C.......................................................................
lonI63_1                                                                                             ..................C..........................A.............................C.....C.......................................................................
lonI63_1                                                                                             ..................C..........................A.............................C.....C.......................................................................
lonI63_1                                                                                             ..................C..........................A.............................C.....C.......................................................................
lonI63_1                                                                                             ..................C..........................A.............................C.....C.......................................................................
lonI63_1                                                                                             ..................C..........................A.............................C.....C.......................................................................
lonI63_2                                                                                             ..................C..........................A.............................C.....C.......................................................................
lonI63_2                                                                                             ..................C..........................A.............................C.....C.......................................................................
lonI63_2                                                                                             ..................C..........................A.............................C.....C.......................................................................
lonI63_2                                                                                             ..................C..........................A.............................C.....C.......................................................................
lonI63_2                                                                                             ..................C..........................A.............................C.....C.......................................................................
lonI63_2                                                                                             ..................C..........................A.............................C.....C.......................................................................
lonI63_2                                                                                             ..................C..........................A.............................C.....C.......................................................................
lonI63_2                                                                                             ..................C..........................A.............................C.....C.......................................................................
lonI63_1                                                                                                                                                                                                                  CAACTATCACACATCAACTGCAA..............................................T..........................................................................................
lonI63_1                                                                                                                                                                                                                                         ..............................................T..........................................................................................
lonI63_1                                                                                                                                                                                                                                         ..............................................T..........................................................................................
lonI63_1                                                                                                                                                                                                                                         ..............................................T..........................................................................................
lonI63_1                                                                                                                                                                                                                                         ..............................................T..........................................................................................
lonI63_1                                                                                                                                                                                                                                         ..............................................T..........................................................................................
lonI63_1                                                                                                                                                                                                                                         ..............................................T..........................................................................................
lonI63_1                                                                                                                                                                                                                                         ..............................................T..........................................................................................
lonI63_2                                                                                                                                                                                                                                         ..............................................T..........................................................................................
lonI63_2                                                                                                                                                                                                                                         ..............................................T..........................................................................................
lonI63_2                                                                                                                                                                                                                                         ..............................................T..........................................................................................
lonI63_2                                                                                                                                                                                                                                         ..............................................T..........................................................................................
lonI63_2                                                                                                                                                                                                                                         ..............................................T..........................................................................................
lonI63_2                                                                                                                                                                                                                                         ..............................................T..........................................................................................
lonI63_2                                                                                                                                                                                                                                         ..............................................T..........................................................................................
lonI63_2                                                                                                                                                                                                                                         ..............................................T..........................................................................................

lonI63 consensus
                 TTCTTTCATGGGGAAGCAGATTTGGGTACCACCCAAGTATTGACTCACCCATCAACAACCGCTATGTATTTCGTACATTACTGCCAGCCACCATGAATATTGCACGGTACCATAAATACTTGACCACCTATAGTACATAAAAACCCAATCCACATCAAACCCCCCCCCCATGCTTACAAGCAAGTACAGCAATCAACCCTCAACTATCACACATCAACTGCAACTCCAAAGCCACCCCTCACCCACTAGGATACCAACAAACCTACCCATCCTTAACAGTACATAGTACATAAAGCCATTTACCGTACATAGCACATTACAGTCAAATCCCTTCTCGTCCCCATGGATGACCCCCCTCAG
```

```
             1111111111111111111111111111111111111111111111111111111111111111111111111111111111111111111111111111111111111111111111111111111111111111111111111111111111111111111111111111111111111111111111111111111111111111111111111111111111111111111111111111111111111111111111111111111111111111111111111111111111111111111111111111111111111111111111111111111111111111111111
             6666666666666666666666666666666666666666666666666666666666666666666666666666666666666666666666666666666666666666666666666666666666666666666666666666666666666666666666666666666666666666666666666666666666666666666666666666666666666666666666666666666666666666666666666666666666666666666666666666666666666666666666666666666666666666666666666666666666666666666666
             0000000000000000000000000000000000000000000000000000000000000000000000000000111111111111111111111111111111111111111111111111111111111111111111111111111111111111111111111111111122222222222222222222222222222222222222222222222222222222222222222222222222222222222222222222222222223333333333333333333333333333333333333333333333333333333333333333333333333333333333333
             2222223333333333444444444455555555556666666666777777777788888888889999999999000000000011111111112222222222333333333344444444445555555555666666666677777777778888888888999999999900000000001111111111222222222233333333334444444444555555555566666666667777777777888888888899999999990000000000111111111122222222223333333333444444444455555555556666666666777777777788888
             4567890123456789012345678901234567890123456789012345678901234567890123456789012345678901234567890123456789012345678901234567890123456789012345678901234567890123456789012345678901234567890123456789012345678901234567890123456789012345678901234567890123456789012345678901234567890123456789012345678901234567890123456789012345678901234567890123456789012345678901234

CRS          TTCTTTCATGGGGAAGCAGATTTGGGTACCACCCAAGTATTGACTCACCCATCAACAACCGCTATGTATTTCGTACATTACTGCCAGCCACCATGAATATTGTACGGTACCATAAATACTTGACCACCTGTAGTACATAAAAACCCAATCCACATCAAAACCCCCTCCCCATGCTTACAAGCAAGTACAGCAATCAACCCTCAACTATCACACATCAACTGCAACTCCAAAGCCACCCCTCACCCACTAGGATACCAACAAACCTACCCACCCTTAACAGTACATAGTACATAAAGCCATTTACCGTACATAGCACATTACAGTCAAATCCCTTCTCGTCCCCATGGATGACCCCCCTCAG

lonII54_1    .............................................T........................................................C.....ACCATAAATACTTGACCACCTGTAG
lonII54_1    .............................................T........................................................C.....
lonII54_1    .............................................T........................................................C.....
lonII54_1    .............................................T........................................................C.....
lonII54_1    .............................................T........................................................C.....
lonII54_1    .............................................T........................................................C.....
lonII54_1    .............................................T........................................................C.....
lonII54_1    .............................................T........................................................C.....
lonII54_2    .............................................T........................................................C.....
lonII54_2    .............................................T........................................................C.....
lonII54_2    .............................................T........................................................C.....
lonII54_2    .............................................T........................................................C.....
lonII54_2    .............................................T........................................................C.....
lonII54_2    .............................................T........................................................C.....
lonII54_2    .............................................T........................................................C.....
lonII54_2    .............................................T........................................................C.....
lonII54_1                                                               CGCTATGTATTTCGTACATTACTGC..................C..........................A...........................................................................................................CCTCACCCACTAGGATACCA
lonII54_1                                                                                        ..................C..........................A...........................................................................................................
lonII54_1                                                                                        ..................C..........................A...........................................................................................................
lonII54_1                                                                                        ..................C..........................A...........................................................................................................
lonII54_1                                                                                        ..................C..........................A...........................................................................................................
lonII54_1                                                                                        ..................C..........................A...........................................................................................................
lonII54_1                                                                                        ..................C..........................A...........................................................................................................
lonII54_1                                                                                        ..................C..........................A...........................................................................................................
lonII54_2                                                                                        ..................C..........................A...........................................................................................................
lonII54_2                                                                                        ..................T..........................A...........................................................................................................
lonII54_2                                                                                        ..................C..........................A...........................................................................................................
lonII54_2                                                                                        ..................C..........................A...........................................................................................................
lonII54_2                                                                                        ..................C..........................A...........................................................................................................
lonII54_2                                                                                        ..................C..........................A...........................................................................................................
lonII54_2                                                                                        ..................C..........................A...........................................................................................................
lonII54_2                                                                                        ..................C..........................A...........................................................................................................
lonII54_1                                                                                                                                                                                                                  CAACTATCACACATCAACTGCAA.........................................................................................................................................
lonII54_1                                                                                                                                                                                                                                         .........................................................................................................................................
lonII54_1                                                                                                                                                                                                                                         .........................................................................................................................................
lonII54_1                                                                                                                                                                                                                                         .........................................................................................................................................
lonII54_1                                                                                                                                                                                                                                         .........................................................................................................................................
lonII54_1                                                                                                                                                                                                                                         .........................................................................................................................................
lonII54_1                                                                                                                                                                                                                                         .........................................................................................................................................
lonII54_1                                                                                                                                                                                                                                         .........................................................................................................................................
lonII54_2                                                                                                                                                                                                                                         .........................................................................................................................................
lonII54_2                                                                                                                                                                                                                                         .........................................................................................................................................
lonII54_2                                                                                                                                                                                                                                         .........................................................................................................................................
lonII54_2                                                                                                                                                                                                                                         .........................................................................................................................................
lonII54_2                                                                                                                                                                                                                                         .........................................................................................................................................
lonII54_2                                                                                                                                                                                                                                         .........................................................................................................................................
lonII54_2                                                                                                                                                                                                                                         .........................................................................................................................................
lonII54_2                                                                                                                                                                                                                                         .........................................................................................................................................

lonII54 consensus
             TTCTTTCATGGGGAAGCAGATTTGGGTACCACCCAAGTATTGACTTACCCATCAACAACCGCTATGTATTTCGTACATTACTGCCAGCCACCATGAATATTGCACGGTACCATAAATACTTGACCACCTATAGTACATAAAAACCCAATCCACATCAAAACCCCCTCCCCATGCTTACAAGCAAGTACAGCAATCAACCCTCAACTATCACACATCAACTGCAACTCCAAAGCCACCCCTCACCCACTAGGATACCAACAAACCTACCCACCCTTAACAGTACATAGTACATAAAGCCATTTACCGTACATAGCACATTACAGTCAAATCCCTTCTCGTCCCCATGGATGACCCCCCTCAG


             1111111111111111111111111111111111111111111111111111111111111111111111111111111111111111111111111111111111111111111111111111111111111111111111111111111111111111111111111111111111111111111111111111111111111111111111111111111111111111111111111111111111111111111111111111111111111111111111111111111111111111111111111111111111111111111111111111111111111111111111
             6666666666666666666666666666666666666666666666666666666666666666666666666666666666666666666666666666666666666666666666666666666666666666666666666666666666666666666666666666666666666666666666666666666666666666666666666666666666666666666666666666666666666666666666666666666666666666666666666666666666666666666666666666666666666666666666666666666666666666666666
             0000000000000000000000000000000000000000000000000000000000000000000000000000111111111111111111111111111111111111111111111111111111111111111111111111111111111111111111111111111122222222222222222222222222222222222222222222222222222222222222222222222222222222222222222222222222223333333333333333333333333333333333333333333333333333333333333333333333333333333333333
             2222223333333333444444444455555555556666666666777777777788888888889999999999000000000011111111112222222222333333333344444444445555555555666666666677777777778888888888999999999900000000001111111111222222222233333333334444444444555555555566666666667777777777888888888899999999990000000000111111111122222222223333333333444444444455555555556666666666777777777788888
             4567890123456789012345678901234567890123456789012345678901234567890123456789012345678901234567890123456789012345678901234567890123456789012345678901234567890123456789012345678901234567890123456789012345678901234567890123456789012345678901234567890123456789012345678901234567890123456789012345678901234567890123456789012345678901234567890123456789012345678901234

CRS          TTCTTTCATGGGGAAGCAGATTTGGGTACCACCCAAGTATTGACTCACCCATCAACAACCGCTATGTATTTCGTACATTACTGCCAGCCACCATGAATATTGTACGGTACCATAAATACTTGACCACCTGTAGTACATAAAAACCCAATCCACATCAAAACCCCCTCCCCATGCTTACAAGCAAGTACAGCAATCAACCCTCAACTATCACACATCAACTGCAACTCCAAAGCCACCCCTCACCCACTAGGATACCAACAAACCTACCCACCCTTAACAGTACATAGTACATAAAGCCATTTACCGTACATAGCACATTACAGTCAAATCCCTTCTCGTCCCCATGGATGACCCCCCTCAG

lonIII2_1    ............................................................................................................ACCATAAATACTTGACCACCTGTAG
lonIII2_1    ............................................................................................................
lonIII2_1    ............................................................................................................
lonIII2_1    ............................................................................................................
lonIII2_1    ............................................................................................................
lonIII2_1    ............................................................................................................
lonIII2_1    ............................................................................................................
lonIII2_1    ............................................................................................................
lonIII2_2    ............................................................................................................
lonIII2_2    ............................................................................................................
lonIII2_2    ............................................................................................................
lonIII2_2    ............................................................................................................
lonIII2_2    ............................................................................................................
lonIII2_2    ............................................................................................................
lonIII2_2    ............................................................................................................
lonIII2_2    ............................................................................................................
lonIII2_1                                                               CGCTATGTATTTCGTACATTACTGC.........................................................................................................................................................CCTCACCCACTAGGATACCA
lonIII2_1                                                                                        .........................................................................................................................................................
lonIII2_1                                                                                        .........................................................................................................................................................
lonIII2_1                                                                                        .........................................................................................................................................................
lonIII2_1                                                                                        .........................................................................................................................................................
lonIII2_1                                                                                        .........................................................................................................................................................
lonIII2_1                                                                                        .........................................................................................................................................................
lonIII2_1                                                                                        .........................................................................................................................................................
lonIII2_2                                                                                        .........................................................................................................................................................
lonIII2_2                                                                                        .........................................................................................................................................................
lonIII2_2                                                                                        .........................................................................................................................................................
lonIII2_2                                                                                        .........................................................................................................................................................
lonIII2_2                                                                                        .........................................................................................................................................................
lonIII2_2                                                                                        .........................................................................................................................................................
lonIII2_2                                                                                        .........................................................................................................................................................
lonIII2_2                                                                                        .........................................................................................................................................................
lonIII2_1                                                                                                                                                                                                                  CAACTATCACACATCAACTGCAA...............................................................C.........................................................................
lonIII2_1                                                                                                                                                                                                                                         ...............................................................C.........................................................................
lonIII2_1                                                                                                                                                                                                                                         ...............................................................C.........................................................................
lonIII2_1                                                                                                                                                                                                                                         ...............................................................C.........................................................................
lonIII2_1                                                                                                                                                                                                                                         ...............................................................C.........................................................................
lonIII2_1                                                                                                                                                                                                                                         ...............................................................C.........................................................................
lonIII2_1                                                                                                                                                                                                                                         ...............................................................C.........................................................................
lonIII2_1                                                                                                                                                                                                                                         ...............................................................C.........................................................................
lonIII2_2                                                                                                                                                                                                                                         ...............................................................C.........................................................................
lonIII2_2                                                                                                                                                                                                                                         ...............................................................C.........................................................................
lonIII2_2                                                                                                                                                                                                                                         ...............................................................C.........................................................................
lonIII2_2                                                                                                                                                                                                                                         ...............................................................C.........................................................................
lonIII2_2                                                                                                                                                                                                                                         ...............................................................C.........................................................................
lonIII2_2                                                                                                                                                                                                                                         ...............................................................C.........................................................................
lonIII2_2                                                                                                                                                                                                                                         ...............................................................C.........................................................................
lonIII2_2                                                                                                                                                                                                                                         ...............................................................C.........................................................................

lonIII2 consensus
             TTCTTTCATGGGGAAGCAGATTTGGGTACCACCCAAGTATTGACTCACCCATCAACAACCGCTATGTATTTCGTACATTACTGCCAGCCACCATGAATATTGTACGGTACCATAAATACTTGACCACCTGTAGTACATAAAAACCCAATCCACATCAAAACCCCCTCCCCATGCTTACAAGCAAGTACAGCAATCAACCCTCAACTATCACACATCAACTGCAACTCCAAAGCCACCCCTCACCCACTAGGATACCAACAAACCTACCCACCCTTAACAGTACATAGCACATAAAGCCATTTACCGTACATAGCACATTACAGTCAAATCCCTTCTCGTCCCCATGGATGACCCCCCTCAG
```

```
              1111111111111111111111111111111111111111111111111111111111111111111111111111111111111111111111111111111111111111111111111111111111111111111111111111111111111111111111111111111111111111111111111111111111111111111111111111111111111111111111111111111111111111111111111111111111111111111111111111111111111111111111111111111111111111111111111111111111111111111111
              6666666666666666666666666666666666666666666666666666666666666666666666666666666666666666666666666666666666666666666666666666666666666666666666666666666666666666666666666666666666666666666666666666666666666666666666666666666666666666666666666666666666666666666666666666666666666666666666666666666666666666666666666666666666666666666666666666666666666666666666
              0000000000000000000000000000000000000000000000000000000000000000000000000000111111111111111111111111111111111111111111111111111111111111111111111111111111111111111111111111111122222222222222222222222222222222222222222222222222222222222222222222222222222222222222222222222222223333333333333333333333333333333333333333333333333333333333333333333333333333333333333
              2222223333333333444444444455555555556666666666777777777788888888889999999999000000000011111111112222222222333333333344444444445555555555666666666677777777778888888888999999999900000000001111111111222222222233333333334444444444555555555566666666667777777777888888888899999999990000000000111111111122222222223333333333444444444455555555556666666666777777777788888
              4567890123456789012345678901234567890123456789012345678901234567890123456789012345678901234567890123456789012345678901234567890123456789012345678901234567890123456789012345678901234567890123456789012345678901234567890123456789012345678901234567890123456789012345678901234567890123456789012345678901234567890123456789012345678901234567890123456789012345678901234

CRS           TTCTTTCATGGGGAAGCAGATTTGGGTACCACCCAAGTATTGACTCACCCATCAACAACCGCTATGTATTTCGTACATTACTGCCAGCCACCATGAATATTGTACGGTACCATAAATACTTGACCACCTGTAGTACATAAAAACCCAATCCACATCAAAACCCCCTCCCCATGCTTACAAGCAAGTACAGCAATCAACCCTCAACTATCACACATCAACTGCAACTCCAAAGCCACCCCTCACCCACTAGGATACCAACAAACCTACCCACCCTTAACAGTACATAGTACATAAAGCCATTTACCGTACATAGCACATTACAGTCAAATCCCTTCTCGTCCCCATGGATGACCCCCCTCAG

lonIII20_1    ............................................................................................................ACCATAAATACTTGACCACCTGTAG
lonIII20_1    ............................................................................................................
lonIII20_1    ............................................................................................................
lonIII20_1    ............................................................................................................
lonIII20_1    ............................................................................................................
lonIII20_1    ............................................................................................................
lonIII20_1    ............................................................................................................
lonIII20_1    ............................................................................................................
lonIII20_2    ............................................................................................................
lonIII20_2    ............................................................................................................
lonIII20_2    ............................................................................................................
lonIII20_2    ............................................................................................................
lonIII20_2    ............................................................................................................
lonIII20_2    ............................................................................................................
lonIII20_2    ............................................................................................................
lonIII20_2    ............................................................................................................
lonIII20_1                                                               CGCTATGTATTTCGTACATTACTGC.........................................................................................................................................................CCTCACCCACTAGGATACCA
lonIII20_1                                                                                        .........................................................................................................................................................
lonIII20_1                                                                                        .........................................................................................................................................................
lonIII20_1                                                                                        .........................................................................................................................................................
lonIII20_1                                                                                        .........................................................................................................................................................
lonIII20_1                                                                                        .........................................................................................................................................................
lonIII20_1                                                                                        .........................................................................................................................................................
lonIII20_1                                                                                        .........................................................................................................................................................
lonIII20_2                                                                                        .........................................................................................................................................................
lonIII20_2                                                                                        .........................................................................................................................................................
lonIII20_2                                                                                        .........................................................................................................................................................
lonIII20_2                                                                                        .........................................................................................................................................................
lonIII20_2                                                                                        .........................................................................................................................................................
lonIII20_2                                                                                        .........................................................................................................................................................
lonIII20_2                                                                                        .........................................................................................................................................................
lonIII20_2                                                                                        .........................................................................................................................................................
lonIII20_1                                                                                                                                                                                                                   CAACTATCACACATCAACTGCAA.........................................................................................................................................
lonIII20_1                                                                                                                                                                                                                                          .........................................................................................................................................
lonIII20_1                                                                                                                                                                                                                                          .........................................................................................................................................
lonIII20_1                                                                                                                                                                                                                                          .........................................................................................................................................
lonIII20_1                                                                                                                                                                                                                                          .........................................................................................................................................
lonIII20_1                                                                                                                                                                                                                                          .........................................................................................................................................
lonIII20_1                                                                                                                                                                                                                                          .........................................................................................................................................
lonIII20_1                                                                                                                                                                                                                                          .........................................................................................................................................
lonIII20_2                                                                                                                                                                                                                                          .........................................................................................................................................
lonIII20_2                                                                                                                                                                                                                                          .........................................................................................................................................
lonIII20_2                                                                                                                                                                                                                                          .........................................................................................................................................
lonIII20_2                                                                                                                                                                                                                                          .........................................................................................................................................
lonIII20_2                                                                                                                                                                                                                                          .........................................................................................................................................
lonIII20_2                                                                                                                                                                                                                                          .........................................................................................................................................
lonIII20_2                                                                                                                                                                                                                                          .........................................................................................................................................
lonIII20_2                                                                                                                                                                                                                                          .........................................................................................................................................

lonIII20 consensus
              TTCTTTCATGGGGAAGCAGATTTGGGTACCACCCAAGTATTGACTCACCCATCAACAACCGCTATGTATTTCGTACATTACTGCCAGCCACCATGAATATTGTACGGTACCATAAATACTTGACCACCTGTAGTACATAAAAACCCAATCCACATCAAAACCCCCTCCCCATGCTTACAAGCAAGTACAGCAATCAACCCTCAACTATCACACATCAACTGCAACTCCAAAGCCACCCCTCACCCACTAGGATACCAACAAACCTACCCACCCTTAACAGTACATAGTACATAAAGCCATTTACCGTACATAGCACATTACAGTCAAATCCCTTCTCGTCCCCATGGATGACCCCCCTCAG
```

```
              1111111111111111111111111111111111111111111111111111111111111111111111111111111111111111111111111111111111111111111111111111111111111111111111111111111111111111111111111111111111111111111111111111111111111111111111111111111111111111111111111111111111111111111111111111111111111111111111111111111111111111111111111111111111111111111111111111111111111111111111
              6666666666666666666666666666666666666666666666666666666666666666666666666666666666666666666666666666666666666666666666666666666666666666666666666666666666666666666666666666666666666666666666666666666666666666666666666666666666666666666666666666666666666666666666666666666666666666666666666666666666666666666666666666666666666666666666666666666666666666666666
              0000000000000000000000000000000000000000000000000000000000000000000000000000111111111111111111111111111111111111111111111111111111111111111111111111111111111111111111111111111122222222222222222222222222222222222222222222222222222222222222222222222222222222222222222222222222223333333333333333333333333333333333333333333333333333333333333333333333333333333333333
              2222223333333333444444444455555555556666666666777777777788888888889999999999000000000011111111112222222222333333333344444444445555555555666666666677777777778888888888999999999900000000001111111111222222222233333333334444444444555555555566666666667777777777888888888899999999990000000000111111111122222222223333333333444444444455555555556666666666777777777788888
              4567890123456789012345678901234567890123456789012345678901234567890123456789012345678901234567890123456789012345678901234567890123456789012345678901234567890123456789012345678901234567890123456789012345678901234567890123456789012345678901234567890123456789012345678901234567890123456789012345678901234567890123456789012345678901234567890123456789012345678901234

CRS           TTCTTTCATGGGGAAGCAGATTTGGGTACCACCCAAGTATTGACTCACCCATCAACAACCGCTATGTATTTCGTACATTACTGCCAGCCACCATGAATATTGTACGGTACCATAAATACTTGACCACCTGTAGTACATAAAAACCCAATCCACATCAAAACCCCCTCCCCATGCTTACAAGCAAGTACAGCAATCAACCCTCAACTATCACACATCAACTGCAACTCCAAAGCCACCCCTCACCCACTAGGATACCAACAAACCTACCCACCCTTAACAGTACATAGTACATAAAGCCATTTACCGTACATAGCACATTACAGTCAAATCCCTTCTCGTCCCCATGGATGACCCCCCTCAG

lonIII34_1    ............................................................................................................ACCATAAATACTTGACCACCTGTAG
lonIII34_1    ............................................................................................................
lonIII34_1    ............................................................................................................
lonIII34_1    ............................................................................................................
lonIII34_1    ............................................................................................................
lonIII34_1    ............................................................................................................
lonIII34_1    ............................................................................................................
lonIII34_1    ............................................................................................................
lonIII34_2    ............................................................................................................
lonIII34_2    ............................................................................................................
lonIII34_2    ............................................................................................................
lonIII34_2    ............................................................................................................
lonIII34_2    ............................................................................................................
lonIII34_2    ............................................................................................................
lonIII34_2    ............................................................................................................
lonIII34_2    ............................................................................................................
lonIII34_1                                                               CGCTATGTATTTCGTACATTACTGC....................................................................................................................................................T....CCTCACCCACTAGGATACCA
lonIII34_1                                                                                        ....................................................................................................................................................T....
lonIII34_1                                                                                        ....................................................................................................................................................T....
lonIII34_1                                                                                        ....................................................................................................................................................T....
lonIII34_1                                                                                        ....................................................................................................................................................T....
lonIII34_1                                                                                        ....................................................................................................................................................T....
lonIII34_1                                                                                        ....................................................................................................................................................T....
lonIII34_1                                                                                        ....................................................................................................................................................T....
lonIII34_2                                                                                        ....................................................................................................................................................T....
lonIII34_2                                                                                        ....................................................................................................................................................T....
lonIII34_2                                                                                        ....................................................................................................................................................T....
lonIII34_2                                                                                        ....................................................................................................................................................T....
lonIII34_2                                                                                        ....................................................................................................................................................T....
lonIII34_2                                                                                        ....................................................................................................................................................T....
lonIII34_2                                                                                        ....................................................................................................................................................T....
lonIII34_2                                                                                        ....................................................................................................................................................T....
lonIII34_1                                                                                                                                                                                                                   CAACTATCACACATCAACTGCAA........T.............T.......................T..........................................................................................
lonIII34_1                                                                                                                                                                                                                                          ........T.............T.......................T..........................................................................................
lonIII34_1                                                                                                                                                                                                                                          ........T.............T.......................T..........................................................................................
lonIII34_1                                                                                                                                                                                                                                          ........T.............T.......................T..........................................................................................
lonIII34_1                                                                                                                                                                                                                                          ........T.............T.......................T..........................................................................................
lonIII34_1                                                                                                                                                                                                                                          ........T.............T.......................T..........................................................................................
lonIII34_1                                                                                                                                                                                                                                          ........T.............T.......................T..........................................................................................
lonIII34_1                                                                                                                                                                                                                                          ........T.............T.......................T..........................................................................................
lonIII34_2                                                                                                                                                                                                                                          ........T.............T.......................T..........................................................................................
lonIII34_2                                                                                                                                                                                                                                          ........T.............T.......................T..........................................................................................
lonIII34_2                                                                                                                                                                                                                                          ........T.............T.......................T..........................................................................................
lonIII34_2                                                                                                                                                                                                                                          ........T.............T.......................T..........................................................................................
lonIII34_2                                                                                                                                                                                                                                          ........T.............T.......................T..........................................................................................
lonIII34_2                                                                                                                                                                                                                                          ........T.............T.......................T..........................................................................................
lonIII34_2                                                                                                                                                                                                                                          ........T.............T.......................T..........................................................................................
lonIII34_2                                                                                                                                                                                                                                          ........T.............T.......................T..........................................................................................
lonIII34_3                                                                                                                                                                          ................................................................................................................................................................................................
lonIII34_3                                                                                                                                                                          ......................................................................T.............T.......................T...................................................................................
lonIII34_3                                                                                                                                                                          ......................................................................T.............T.......................T...................................................................................
lonIII34_3                                                                                                                                                                          ....................................................................................T.......................T...................................................................................
lonIII34_3                                                                                                                                                                          ...........T..............................T...........................T.............T.......................T.........C.........................................................................
lonIII34_3                                                                                                                                                                          ......................................................................T..TT...............-............T...............C...G.........A...................T.............T........................
lonIII34_3                                                                                                                                                                          ...........T..............................T.............T...................T............................T...........................A..........................................................
lonIII34_3                                                                                                                                                                          ..........................................T.........................................T.......................T...............................................................T...................
lonIII34_3                                                                                                                                                                          ............................................................................................................................................................................T...................
lonIII34_3                                                                                                                                                                          ...........................................................................................................................G....................................................................

lonIII34 consensus
              TTCTTTCATGGGGAAGCAGATTTGGGTACCACCCAAGTATTGACTCACCCATCAACAACCGCTATGTATTTCGTACATTACTGCCAGCCACCATGAATATTGTACGGTACCATAAATACTTGACCACCTGTAGTACATAAAAACCCAATCCACATCAAAACCCCCTCCCCATGCTTACAAGCAAGTACAGCAATCAACCCTCAACTATCACACATCAACTGCAACTCCAAAGTCACCCCTCACCCATTAGGATACCAACAAACCTACCCATCCTTAACAGTACATAGTACATAAAGCCATTTACCGTACATAGCACATTACAGTCAAATCCCTTCTCGTCCCCATGGATGACCCCCCTCAG
```

```
              1111111111111111111111111111111111111111111111111111111111111111111111111111111111111111111111111111111111111111111111111111111111111111111111111111111111111111111111111111111111111111111111111111111111111111111111111111111111111111111111111111111111111111111111111111111111111111111111111111111111111111111111111111111111111111111111111111111111111111111
              6666666666666666666666666666666666666666666666666666666666666666666666666666666666666666666666666666666666666666666666666666666666666666666666666666666666666666666666666666666666666666666666666666666666666666666666666666666666666666666666666666666666666666666666666666666666666666666666666666666666666666666666666666666666666666666666666666666666666666666
              0000000000000000000000000000000000000000000000000000000000000000000000000000111111111111111111111111111111111111111111111111111111111111111111111111111111111111111111111111111122222222222222222222222222222222222222222222222222222222222222222222222222222222222222222222222222223333333333333333333333333333333333333333333333333333333333333333333333333333333333333
              2222223333333333444444444455555555556666666666777777777788888888889999999999000000000011111111112222222222333333333344444444445555555555666666666677777777778888888888999999999900000000001111111111222222222233333333334444444444555555555566666666667777777777888888888899999999990000000000111111111122222222223333333333444444444455555555556666666666777777777788888
              4567890123456789012345678901234567890123456789012345678901234567890123456789012345678901234567890123456789012345678901234567890123456789012345678901234567890123456789012345678901234567890123456789012345678901234567890123456789012345678901234567890123456789012345678901234567890123456789012345678901234567890123456789012345678901234567890123456789012345678901234

CRS           TTCTTTCATGGGGAAGCAGATTTGGGTACCACCCAAGTATTGACTCACCCATCAACAACCGCTATGTATTTCGTACATTACTGCCAGCCACCATGAATATTGTACGGTACCATAAATACTTGACCACCTGTAGTACATAAAAACCCAATCCACATCAAAACCCCCTCCCCATGCTTACAAGCAAGTACAGCAATCAACCCTCAACTATCACACATCAACTGCAACTCCAAAGCCACCCCTCACCCACTAGGATACCAACAAACCTACCCACCCTTAACAGTACATAGTACATAAAGCCATTTACCGTACATAGCACATTACAGTCAAATCCCTTCTCGTCCCCATGGATGACCCCCCTCAG

per4_1        ............................................................................................................ACCATAAATACTTGACCACCTGTAG
per4_1        ............................................................................................................
per4_1        ............................................................................................................
per4_1        ............................................................................................................
per4_1        ............................................................................................................
per4_1        ............................................................................................................
per4_1        ............................................................................................................
per4_1        ............................................................................................................
per4_2        ............................................................................................................
per4_2        ............................................................................................................
per4_2        ............................................................................................................
per4_2        ............................................................................................................
per4_2        ............................................................................................................
per4_2        ............................................................................................................
per4_2        ............................................................................................................
per4_2        ............................................................................................................
per4_1                                                                   CGCTATGTATTTCGTACATTACTGC.........................................................................................................................................................CCTCACCCACTAGGATACCA
per4_1                                                                                            .........................................................................................................................................................
per4_1                                                                                            .........................................................................................................................................................
per4_1                                                                                            .........................................................................................................................................................
per4_1                                                                                            .........................................................................................................................................................
per4_1                                                                                            .........................................................................................................................................................
per4_1                                                                                            .........................................................................................................................................................
per4_1                                                                                            .........................................................................................................................................................
per4_2                                                                                            .........................................................................................................................................................
per4_2                                                                                            .........................................................................................................................................................
per4_2                                                                                            .........................................................................................................................................................
per4_2                                                                                            .........................................................................................................................................................
per4_2                                                                                            .........................................................................................................................................................
per4_2                                                                                            .........................................................................................................................................................
per4_2                                                                                            .........................................................................................................................................................
per4_2                                                                                            .........................................................................................................................................................
per4_1                                                                                                                                                                                                                 CAACTATCACACATCAACTGCAA..................................................................................................................C......................
per4_1                                                                                                                                                                                                                                        ..................................................................................................................C......................
per4_1                                                                                                                                                                                                                                        ..................................................................................................................C......................
per4_1                                                                                                                                                                                                                                        ..................................................................................................................C......................
per4_1                                                                                                                                                                                                                                        ..................................................................................................................C......................
per4_1                                                                                                                                                                                                                                        ..................................................................................................................C......................
per4_1                                                                                                                                                                                                                                        ..................................................................................................................C......................
per4_1                                                                                                                                                                                                                                        ..................................................................................................................C......................
per4_2                                                                                                                                                                                                                                        ..................................................................................................................C......................
per4_2                                                                                                                                                                                                                                        ..................................................................................................................C......................
per4_2                                                                                                                                                                                                                                        ..................................................................................................................C......................
per4_2                                                                                                                                                                                                                                        ..................................................................................................................C......................
per4_2                                                                                                                                                                                                                                        ..................................................................................................................C......................
per4_2                                                                                                                                                                                                                                        ..................................................................................................................C......................
per4_2                                                                                                                                                                                                                                        ..................................................................................................................C......................
per4_2                                                                                                                                                                                                                                        ..................................................................................................................C......................

per4 consensus
              TTCTTTCATGGGGAAGCAGATTTGGGTACCACCCAAGTATTGACTCACCCATCAACAACCGCTATGTATTTCGTACATTACTGCCAGCCACCATGAATATTGTACGGTACCATAAATACTTGACCACCTGTAGTACATAAAAACCCAATCCACATCAAAACCCCCTCCCCATGCTTACAAGCAAGTACAGCAATCAACCCTCAACTATCACACATCAACTGCAACTCCAAAGCCACCCCTCACCCACTAGGATACCAACAAACCTACCCACCCTTAACAGTACATAGTACATAAAGCCATTTACCGTACATAGCACATTACAGTCAAATCCCTTCTCGCCCCCATGGATGACCCCCCTCAG


              1111111111111111111111111111111111111111111111111111111111111111111111111111111111111111111111111111111111111111111111111111111111111111111111111111111111111111111111111111111111111111111111111111111111111111111111111111111111111111111111111111111111111111111111111111111111111111111111111111111111111111111111111111111111111111111111111111111111111111111
              6666666666666666666666666666666666666666666666666666666666666666666666666666666666666666666666666666666666666666666666666666666666666666666666666666666666666666666666666666666666666666666666666666666666666666666666666666666666666666666666666666666666666666666666666666666666666666666666666666666666666666666666666666666666666666666666666666666666666666666
              0000000000000000000000000000000000000000000000000000000000000000000000000000111111111111111111111111111111111111111111111111111111111111111111111111111111111111111111111111111122222222222222222222222222222222222222222222222222222222222222222222222222222222222222222222222222223333333333333333333333333333333333333333333333333333333333333333333333333333333333333
              2222223333333333444444444455555555556666666666777777777788888888889999999999000000000011111111112222222222333333333344444444445555555555666666666677777777778888888888999999999900000000001111111111222222222233333333334444444444555555555566666666667777777777888888888899999999990000000000111111111122222222223333333333444444444455555555556666666666777777777788888
              4567890123456789012345678901234567890123456789012345678901234567890123456789012345678901234567890123456789012345678901234567890123456789012345678901234567890123456789012345678901234567890123456789012345678901234567890123456789012345678901234567890123456789012345678901234567890123456789012345678901234567890123456789012345678901234567890123456789012345678901234

CRS           TTCTTTCATGGGGAAGCAGATTTGGGTACCACCCAAGTATTGACTCACCCATCAACAACCGCTATGTATTTCGTACATTACTGCCAGCCACCATGAATATTGTACGGTACCATAAATACTTGACCACCTGTAGTACATAAAAACCCAATCCACATCAAAACCCCCTCCCCATGCTTACAAGCAAGTACAGCAATCAACCCTCAACTATCACACATCAACTGCAACTCCAAAGCCACCCCTCACCCACTAGGATACCAACAAACCTACCCACCCTTAACAGTACATAGTACATAAAGCCATTTACCGTACATAGCACATTACAGTCAAATCCCTTCTCGTCCCCATGGATGACCCCCCTCAG

per3F_1       ..........................................................................................A.................ACCATAAATACTTGACCACCTGTAG
per3F_1       ..........................................................................................A.................
per3F_1       ..........................................................................................A.................
per3F_1       ..........................................................................................A.................
per3F_1       ..........................................................................................A.................
per3F_1       ..........................................................................................A.................
per3F_1       ..........................................................................................A.................
per3F_1       ..........................................................................................A.................
per3F_2       ..........................................................................................A.................
per3F_2       ..........................................................................................A.................
per3F_2       ..........................................................................................A.................
per3F_2       ..........................................................................................A.................
per3F_2       ..........................................................................................A.................
per3F_2       ..........................................................................................A.................
per3F_2       ..........................................................................................A.................
per3F_2       ..........................................................................................A.................
per3F_1                                                                  CGCTATGTATTTCGTACATTACTGC......A.............................................................................T...............................................................T....CCTCACCCACTAGGATACCA
per3F_1                                                                                           ......A.............................................................................T...............................................................T....
per3F_1                                                                                           ......A.............................................................................T...............................................................T....
per3F_1                                                                                           ......A.............................................................................T...............................................................T....
per3F_1                                                                                           ......A.............................................................................T...............................................................T....
per3F_1                                                                                           ......A.............................................................................T...............................................................T....
per3F_1                                                                                           ......A.............................................................................T...............................................................T....
per3F_1                                                                                           ......A.............................................................................T...............................................................T....
per3F_2                                                                                           ......A.............................................................................T...............................................................T....
per3F_2                                                                                           ......A.............................................................................T...............................................................T....
per3F_2                                                                                           ......A.............................................................................T...............................................................T....
per3F_2                                                                                           ......A.............................................................................T...............................................................T....
per3F_2                                                                                           ......A.............................................................................T...............................................................T....
per3F_2                                                                                           ......A.............................................................................T...............................................................T....
per3F_2                                                                                           ......A.............................................................................T...............................................................T....
per3F_2                                                                                           ......A.............................................................................T...............................................................T....
per3F_1                                                                                                                                                                                                                CAACTATCACACATCAACTGCAA........T.............T.......................T..........................................................................................
per3F_1                                                                                                                                                                                                                                       ........T.............T.......................T..........................................................................................
per3F_1                                                                                                                                                                                                                                       ........T.............T.......................T..........................................................................................
per3F_1                                                                                                                                                                                                                                       ........T.............T.......................T..........................................................................................
per3F_1                                                                                                                                                                                                                                       ........T.............T.......................T..........................................................................................
per3F_1                                                                                                                                                                                                                                       ........T.............T.......................T..........................................................................................
per3F_1                                                                                                                                                                                                                                       ........T.............T.......................T..........................................................................................
per3F_1                                                                                                                                                                                                                                       ........T.............T.......................T..........................................................................................
per3F_2                                                                                                                                                                                                                                       ........T.............T.......................T..........................................................................................
per3F_2                                                                                                                                                                                                                                       ........T.............T.......................T..........................................................................................
per3F_2                                                                                                                                                                                                                                       ........T.............T.......................T..........................................................................................
per3F_2                                                                                                                                                                                                                                       ........T.............T.......................T..........................................................................................
per3F_2                                                                                                                                                                                                                                       ........T.............T.......................T..........................................................................................
per3F_2                                                                                                                                                                                                                                       ........T.............T.......................T..........................................................................................
per3F_2                                                                                                                                                                                                                                       ........T.............T.......................T..........................................................................................
per3F_2                                                                                                                                                                                                                                       ........T.............T.......................T..........................................................................................

per3F consensus
              TTCTTTCATGGGGAAGCAGATTTGGGTACCACCCAAGTATTGACTCACCCATCAACAACCGCTATGTATTTCGTACATTACTGCCAGCCAACATGAATATTGTACGGTACCATAAATACTTGACCACCTGTAGTACATAAAAACCCAATCCACATCAAAACCCCCTCCTCATGCTTACAAGCAAGTACAGCAATCAACCCTCAACTATCACACATCAACTGCAACTCCAAAGTCACCCCTCACCCATTAGGATACCAACAAACCTACCCATCCTTAACAGTACATAGTACATAAAGCCATTTACCGTACATAGCACATTACAGTCAAATCCCTTCTCGTCCCCATGGATGACCCCCCTCAG
```

```
                 1111111111111111111111111111111111111111111111111111111111111111111111111111111111111111111111111111111111111111111111111111111111111111111111111111111111111111111111111111111111111111111111111111111111111111111111111111111111111111111111111111111111111111111111111111111111111111111111111111111111111111111111111111111111111111111111111111111111111111111111111
                 6666666666666666666666666666666666666666666666666666666666666666666666666666666666666666666666666666666666666666666666666666666666666666666666666666666666666666666666666666666666666666666666666666666666666666666666666666666666666666666666666666666666666666666666666666666666666666666666666666666666666666666666666666666666666666666666666666666666666666666666666
                 0000000000000000000000000000000000000000000000000000000000000000000000000000111111111111111111111111111111111111111111111111111111111111111111111111111111111111111111111111111111111111222222222222222222222222222222222222222222222222222222222222222222222222222222222222222222222222222222223333333333333333333333333333333333333333333333333333333333333333333333333333333333333
                 2222223333333333444444444455555555556666666666777777777788888888889999999999000000000011111111112222222222333333333344444444445555555555666666666677777777778888888888999999999900000000001111111111222222222233333333334444444444555555555566666666667777777777888888888899999999990000000000111111111122222222223333333333444444444455555555556666666666777777777788888
                 4567890123456789012345678901234567890123456789012345678901234567890123456789012345678901234567890123456789012345678901234567890123456789012345678901234567890123456789012345678901234567890123456789012345678901234567890123456789012345678901234567890123456789012345678901234567890123456789012345678901234567890123456789012345678901234567890123456789012345678901234

CRS              TTCTTTCATGGGGAAGCAGATTTGGGTACCACCCAAGTATTGACTCACCCATCAACAACCGCTATGTATTTCGTACATTACTGCCAGCCACCATGAATATTGTACGGTACCATAAATACTTGACCACCTGTAGTACATAAAAACCCAATCCACATCAAAACCCCCTCCCCATGCTTACAAGCAAGTACAGCAATCAACCCTCAACTATCACACATCAACTGCAACTCCAAAGCCACCCCTCACCCACTAGGATACCAACAAACCTACCCACCCTTAACAGTACATAGTACATAAAGCCATTTACCGTACATAGCACATTACAGTCAAATCCCTTCTCGTCCCCATGGATGACCCCCCTCAG

per9F_1          ............................................................................................................ACCATAAATACTTGACCACCTGTAG
per9F_1          ............................................................................................................
per9F_1          ............................................................................................................
per9F_1          ............................................................................................................
per9F_1          ............................................................................................................
per9F_1          ............................................................................................................
per9F_1          ............................................................................................................
per9F_1          ............................................................................................................
per9F_2          ............................................................................................................
per9F_2          ............................................................................................................
per9F_2          ............................................................................................................
per9F_2          ............................................................................................................
per9F_2          ............................................................................................................
per9F_2          ............................................................................................................
per9F_2          ............................................................................................................
per9F_2          ............................................................................................................
per9F_1                                                                     CGCTATGTATTTCGTACATTACTGC.........................................................................................................................................................CCTCACCCACTAGGATACCA
per9F_1                                                                                                 .........................................................................................................................................................
per9F_1                                                                                                 .........................................................................................................................................................
per9F_1                                                                                                 .........................................................................................................................................................
per9F_1                                                                                                 .........................................................................................................................................................
per9F_1                                                                                                 .........................................................................................................................................................
per9F_1                                                                                                 .........................................................................................................................................................
per9F_1                                                                                                 .........................................................................................................................................................
per9F_2                                                                                                 .........................................................................................................................................................
per9F_2                                                                                                 .........................................................................................................................................................
per9F_2                                                                                                 .........................................................................................................................................................
per9F_2                                                                                                 .........................................................................................................................................................
per9F_2                                                                                                 .........................................................................................................................................................
per9F_2                                                                                                 .........................................................................................................................................................
per9F_2                                                                                                 .........................................................................................................................................................
per9F_2                                                                                                 .........................................................................................................................................................
per9F_1                                                                                                                                                                                                                           CAACTATCACACATCAACTGCAA...............................................................C.........................................................................
per9F_1                                                                                                                                                                                                                                                  ...............................................................C.........................................................................
per9F_1                                                                                                                                                                                                                                                  ...............................................................C.........................................................................
per9F_1                                                                                                                                                                                                                                                  ...............................................................C.........................................................................
per9F_1                                                                                                                                                                                                                                                  ...............................................................C.........................................................................
per9F_1                                                                                                                                                                                                                                                  ...............................................................C.........................................................................
per9F_1                                                                                                                                                                                                                                                  ...............................................................C.........................................................................
per9F_1                                                                                                                                                                                                                                                  ...............................................................C.........................................................................
per9F_2                                                                                                                                                                                                                                                  ...............................................................C.........................................................................
per9F_2                                                                                                                                                                                                                                                  ...............................................................C.........................................................................
per9F_2                                                                                                                                                                                                                                                  ...............................................................C.........................................................................
per9F_2                                                                                                                                                                                                                                                  ...............................................................C.........................................................................
per9F_2                                                                                                                                                                                                                                                  ...............................................................C.........................................................................
per9F_2                                                                                                                                                                                                                                                  ...............................................................C.........................................................................
per9F_2                                                                                                                                                                                                                                                  ...............................................................C.........................................................................
per9F_2                                                                                                                                                                                                                                                  ...............................................................C.........................................................................

per9F consensus
                 TTCTTTCATGGGGAAGCAGATTTGGGTACCACCCAAGTATTGACTCACCCATCAACAACCGCTATGTATTTCGTACATTACTGCCAGCCACCATGAATATTGTACGGTACCATAAATACTTGACCACCTGTAGTACATAAAAACCCAATCCACATCAAAACCCCCTCCCCATGCTTACAAGCAAGTACAGCAATCAACCCTCAACTATCACACATCAACTGCAACTCCAAAGCCACCCCTCACCCACTAGGATACCAACAAACCTACCCACCCTTAACAGTACATAGCACATAAAGCCATTTACCGTACATAGCACATTACAGTCAAATCCCTTCTCGTCCCCATGGATGACCCCCCTCAG
```

```
                 1111111111111111111111111111111111111111111111111111111111111111111111111111111111111111111111111111111111111111111111111111111111111111111111111111111111111111111111111111111111111111111111111111111111111111111111111111111111111111111111111111111111111111111111111111111111111111111111111111111111111111111111111111111111111111111111111111111111111111111111111
                 6666666666666666666666666666666666666666666666666666666666666666666666666666666666666666666666666666666666666666666666666666666666666666666666666666666666666666666666666666666666666666666666666666666666666666666666666666666666666666666666666666666666666666666666666666666666666666666666666666666666666666666666666666666666666666666666666666666666666666666666666
                 0000000000000000000000000000000000000000000000000000000000000000000000000000111111111111111111111111111111111111111111111111111111111111111111111111111111111111111111111111111111111111222222222222222222222222222222222222222222222222222222222222222222222222222222222222222222222222222222223333333333333333333333333333333333333333333333333333333333333333333333333333333333333
                 2222223333333333444444444455555555556666666666777777777788888888889999999999000000000011111111112222222222333333333344444444445555555555666666666677777777778888888888999999999900000000001111111111222222222233333333334444444444555555555566666666667777777777888888888899999999990000000000111111111122222222223333333333444444444455555555556666666666777777777788888
                 4567890123456789012345678901234567890123456789012345678901234567890123456789012345678901234567890123456789012345678901234567890123456789012345678901234567890123456789012345678901234567890123456789012345678901234567890123456789012345678901234567890123456789012345678901234567890123456789012345678901234567890123456789012345678901234567890123456789012345678901234

CRS              TTCTTTCATGGGGAAGCAGATTTGGGTACCACCCAAGTATTGACTCACCCATCAACAACCGCTATGTATTTCGTACATTACTGCCAGCCACCATGAATATTGTACGGTACCATAAATACTTGACCACCTGTAGTACATAAAAACCCAATCCACATCAAAACCCCCTCCCCATGCTTACAAGCAAGTACAGCAATCAACCCTCAACTATCACACATCAACTGCAACTCCAAAGCCACCCCTCACCCACTAGGATACCAACAAACCTACCCACCCTTAACAGTACATAGTACATAAAGCCATTTACCGTACATAGCACATTACAGTCAAATCCCTTCTCGTCCCCATGGATGACCCCCCTCAG

per36_1          ............................................................................................................ACCATAAATACTTGACCACCTGTAG
per36_1          ............................................................................................................
per36_1          ............................................................................................................
per36_1          ............................................................................................................
per36_1          ............................................................................................................
per36_1          ............................................................................................................
per36_1          ............................................................................................................
per36_1          ............................................................................................................
per36_2          ............................................................................................................
per36_2          ............................................................................................................
per36_2          ............................................................................................................
per36_2          ............................................................................................................
per36_2          ............................................................................................................
per36_2          ............................................................................................................
per36_2          ............................................................................................................
per36_2          ............................................................................................................
per36_1                                                                     CGCTATGTATTTCGTACATTACTGC.........................................................................................................................................................CCTCACCCACTAGGATACCA
per36_1                                                                                                 .........................................................................................................................................................
per36_1                                                                                                 .........................................................................................................................................................
per36_1                                                                                                 .........................................................................................................................................................
per36_1                                                                                                 .........................................................................................................................................................
per36_1                                                                                                 .........................................................................................................................................................
per36_1                                                                                                 .........................................................................................................................................................
per36_1                                                                                                 .........................................................................................................................................................
per36_2                                                                                                 .........................................................................................................................................................
per36_2                                                                                                 .........................................................................................................................................................
per36_2                                                                                                 .........................................................................................................................................................
per36_2                                                                                                 .........................................................................................................................................................
per36_2                                                                                                 .........................................................................................................................................................
per36_2                                                                                                 .........................................................................................................................................................
per36_2                                                                                                 .........................................................................................................................................................
per36_2                                                                                                 .........................................................................................................................................................
per36_1                                                                                                                                                                                                                           CAACTATCACACATCAACTGCAA.........................................................................................................................................
per36_1                                                                                                                                                                                                                                                  .........................................................................................................................................
per36_1                                                                                                                                                                                                                                                  .........................................................................................................................................
per36_1                                                                                                                                                                                                                                                  .........................................................................................................................................
per36_1                                                                                                                                                                                                                                                  .........................................................................................................................................
per36_1                                                                                                                                                                                                                                                  .........................................................................................................................................
per36_1                                                                                                                                                                                                                                                  .........................................................................................................................................
per36_1                                                                                                                                                                                                                                                  .........................................................................................................................................
per36_2                                                                                                                                                                                                                                                  .........................................................................................................................................
per36_2                                                                                                                                                                                                                                                  .........................................................................................................................................
per36_2                                                                                                                                                                                                                                                  .........................................................................................................................................
per36_2                                                                                                                                                                                                                                                  .........................................................................................................................................
per36_2                                                                                                                                                                                                                                                  .........................................................................................................................................
per36_2                                                                                                                                                                                                                                                  .........................................................................................................................................
per36_2                                                                                                                                                                                                                                                  .........................................................................................................................................
per36_2                                                                                                                                                                                                                                                  .........................................................................................................................................

per36 consensus
                 TTCTTTCATGGGGAAGCAGATTTGGGTACCACCCAAGTATTGACTCACCCATCAACAACCGCTATGTATTTCGTACATTACTGCCAGCCACCATGAATATTGTACGGTACCATAAATACTTGACCACCTGTAGTACATAAAAACCCAATCCACATCAAAACCCCCTCCCCATGCTTACAAGCAAGTACAGCAATCAACCCTCAACTATCACACATCAACTGCAACTCCAAAGCCACCCCTCACCCACTAGGATACCAACAAACCTACCCACCCTTAACAGTACATAGTACATAAAGCCATTTACCGTACATAGCACATTACAGTCAAATCCCTTCTCGTCCCCATGGATGACCCCCCTCAG
```

```
                1111111111111111111111111111111111111111111111111111111111111111111111111111111111111111111111111111111111111111111111111111111111111111111111111111111111111111111111111111111111111111111111111111111111111111111111111111111111111111111111111111111111111111111111111111111111111111111111111111111111111111111111111111111111111111111111111111111111111111111111111
                6666666666666666666666666666666666666666666666666666666666666666666666666666666666666666666666666666666666666666666666666666666666666666666666666666666666666666666666666666666666666666666666666666666666666666666666666666666666666666666666666666666666666666666666666666666666666666666666666666666666666666666666666666666666666666666666666666666666666666666666666
                0000000000000000000000000000000000000000000000000000000000000000000000000000111111111111111111111111111111111111111111111111111111111111111111111111111111111111111111111111111111112222222222222222222222222222222222222222222222222222222222222222222222222222222222222222222222222222222233333333333333333333333333333333333333333333333333333333333333333333333333333333333333
                2222223333333333444444444455555555556666666666777777777788888888889999999999000000000011111111112222222222333333333344444444445555555555666666666677777777778888888888999999999900000000001111111111222222222233333333334444444444555555555566666666667777777777888888888899999999990000000000111111111122222222223333333333444444444455555555556666666666777777777788888
                4567890123456789012345678901234567890123456789012345678901234567890123456789012345678901234567890123456789012345678901234567890123456789012345678901234567890123456789012345678901234567890123456789012345678901234567890123456789012345678901234567890123456789012345678901234567890123456789012345678901234567890123456789012345678901234567890123456789012345678901234

CRS             TTCTTTCATGGGGAAGCAGATTTGGGTACCACCCAAGTATTGACTCACCCATCAACAACCGCTATGTATTTCGTACATTACTGCCAGCCACCATGAATATTGTACGGTACCATAAATACTTGACCACCTGTAGTACATAAAAACCCAATCCACATCAAAACCCCCTCCCCATGCTTACAAGCAAGTACAGCAATCAACCCTCAACTATCACACATCAACTGCAACTCCAAAGCCACCCCTCACCCACTAGGATACCAACAAACCTACCCACCCTTAACAGTACATAGTACATAAAGCCATTTACCGTACATAGCACATTACAGTCAAATCCCTTCTCGTCCCCATGGATGACCCCCCTCAG

cenII1_1        ............................................................................................................ACCATAAATACTTGACCACCTGTAG
cenII1_1        ............................................................................................................
cenII1_1        ............................................................................................................
cenII1_1        ............................................................................................................
cenII1_1        ............................................................................................................
cenII1_1        ............................................................................................................
cenII1_1        ............................................................................................................
cenII1_1        ............................................................................................................
cenII1_2        ............................................................................................................
cenII1_2        ............................................................................................................
cenII1_2        ............................................................................................................
cenII1_2        ............................................................................................................
cenII1_2        ............................................................................................................
cenII1_2        ............................................................................................................
cenII1_2        ............................................................................................................
cenII1_2        ............................................................................................................
cenII1_1                                                                   CGCTATGTATTTCGTACATTACTGC.........................................................................................................................................................CCTCACCCACTAGGATACCA
cenII1_1                                                                                            .........................................................................................................................................................
cenII1_1                                                                                            .........................................................................................................................................................
cenII1_1                                                                                            .........................................................................................................................................................
cenII1_1                                                                                            .........................................................................................................................................................
cenII1_1                                                                                            .........................................................................................................................................................
cenII1_1                                                                                            .........................................................................................................................................................
cenII1_1                                                                                            .........................................................................................................................................................
cenII1_2                                                                                            .........................................................................................................................................................
cenII1_2                                                                                            .........................................................................................................................................................
cenII1_2                                                                                            .........................................................................................................................................................
cenII1_2                                                                                            .........................................................................................................................................................
cenII1_2                                                                                            .........................................................................................................................................................
cenII1_2                                                                                            .........................................................................................................................................................
cenII1_2                                                                                            .........................................................................................................................................................
cenII1_2                                                                                            .........................................................................................................................................................
cenII1_1                                                                                                                                                                                                                         CAACTATCACACATCAACTGCAA...............................................................C.........................................................................
cenII1_1                                                                                                                                                                                                                                                ...............................................................C.........................................................................
cenII1_1                                                                                                                                                                                                                                                ...............................................................C.........................................................................
cenII1_1                                                                                                                                                                                                                                                ...............................................................C.........................................................................
cenII1_1                                                                                                                                                                                                                                                ...............................................................C.........................................................................
cenII1_1                                                                                                                                                                                                                                                ...............................................................C.........................................................................
cenII1_1                                                                                                                                                                                                                                                ...............................................................C.........................................................................
cenII1_1                                                                                                                                                                                                                                                ...............................................................C.........................................................................
cenII1_2                                                                                                                                                                                                                                                ...............................................................C.........................................................................
cenII1_2                                                                                                                                                                                                                                                ...............................................................C.........................................................................
cenII1_2                                                                                                                                                                                                                                                ...............................................................C.........................................................................
cenII1_2                                                                                                                                                                                                                                                ...............................................................C.........................................................................
cenII1_2                                                                                                                                                                                                                                                ...............................................................C.........................................................................
cenII1_2                                                                                                                                                                                                                                                ...............................................................C.........................................................................
cenII1_2                                                                                                                                                                                                                                                ...............................................................C.........................................................................
cenII1_2                                                                                                                                                                                                                                                ...............................................................C.........................................................................

cenII1 consensus
                TTCTTTCATGGGGAAGCAGATTTGGGTACCACCCAAGTATTGACTCACCCATCAACAACCGCTATGTATTTCGTACATTACTGCCAGCCACCATGAATATTGTACGGTACCATAAATACTTGACCACCTGTAGTACATAAAAACCCAATCCACATCAAAACCCCCTCCCCATGCTTACAAGCAAGTACAGCAATCAACCCTCAACTATCACACATCAACTGCAACTCCAAAGCCACCCCTCACCCACTAGGATACCAACAAACCTACCCACCCTTAACAGTACATAGCACATAAAGCCATTTACCGTACATAGCACATTACAGTCAAATCCCTTCTCGTCCCCATGGATGACCCCCCTCAG
```

```
                1111111111111111111111111111111111111111111111111111111111111111111111111111111111111111111111111111111111111111111111111111111111111111111111111111111111111111111111111111111111111111111111111111111111111111111111111111111111111111111111111111111111111111111111111111111111111111111111111111111111111111111111111111111111111111111111111111111111111111111111111
                6666666666666666666666666666666666666666666666666666666666666666666666666666666666666666666666666666666666666666666666666666666666666666666666666666666666666666666666666666666666666666666666666666666666666666666666666666666666666666666666666666666666666666666666666666666666666666666666666666666666666666666666666666666666666666666666666666666666666666666666666
                0000000000000000000000000000000000000000000000000000000000000000000000000000111111111111111111111111111111111111111111111111111111111111111111111111111111111111111111111111111111112222222222222222222222222222222222222222222222222222222222222222222222222222222222222222222222222222222233333333333333333333333333333333333333333333333333333333333333333333333333333333333333
                2222223333333333444444444455555555556666666666777777777788888888889999999999000000000011111111112222222222333333333344444444445555555555666666666677777777778888888888999999999900000000001111111111222222222233333333334444444444555555555566666666667777777777888888888899999999990000000000111111111122222222223333333333444444444455555555556666666666777777777788888
                4567890123456789012345678901234567890123456789012345678901234567890123456789012345678901234567890123456789012345678901234567890123456789012345678901234567890123456789012345678901234567890123456789012345678901234567890123456789012345678901234567890123456789012345678901234567890123456789012345678901234567890123456789012345678901234567890123456789012345678901234

CRS             TTCTTTCATGGGGAAGCAGATTTGGGTACCACCCAAGTATTGACTCACCCATCAACAACCGCTATGTATTTCGTACATTACTGCCAGCCACCATGAATATTGTACGGTACCATAAATACTTGACCACCTGTAGTACATAAAAACCCAATCCACATCAAAACCCCCTCCCCATGCTTACAAGCAAGTACAGCAATCAACCCTCAACTATCACACATCAACTGCAACTCCAAAGCCACCCCTCACCCACTAGGATACCAACAAACCTACCCACCCTTAACAGTACATAGTACATAAAGCCATTTACCGTACATAGCACATTACAGTCAAATCCCTTCTCGTCCCCATGGATGACCCCCCTCAG

cenII39_1       .............................................T........................................................C.....ACCATAAATACTTGACCACCTGTAG
cenII39_1       .............................................T........................................................C.....
cenII39_1       .............................................T........................................................C.....
cenII39_1       .............................................T........................................................C.....
cenII39_1       .............................................T........................................................C.....
cenII39_1       .............................................T........................................................C.....
cenII39_1       .............................................T........................................................C.....
cenII39_1       .............................................T........................................................C.....
cenII39_2       .............................................T........................................................C.....
cenII39_2       .............................................T........................................................C.....
cenII39_2       .............................................T........................................................C.....
cenII39_2       .............................................T........................................................C.....
cenII39_2       .............................................T........................................................C.....
cenII39_2       .............................................T........................................................C.....
cenII39_2       .............................................T........................................................C.....
cenII39_2       .............................................T........................................................C.....
cenII39_1                                                                  CGCTATGTATTTCGTACATTACTGC..................C......................................................................................................................................CCTCACCCACTAGGATACCA
cenII39_1                                                                                           ..................C......................................................................................................................................
cenII39_1                                                                                           ..................C......................................................................................................................................
cenII39_1                                                                                           ..................C......................................................................................................................................
cenII39_1                                                                                           ..................C......................................................................................................................................
cenII39_1                                                                                           ..................C......................................................................................................................................
cenII39_1                                                                                           ..................C......................................................................................................................................
cenII39_1                                                                                           ..................C......................................................................................................................................
cenII39_2                                                                                           ..................C......................................................................................................................................
cenII39_2                                                                                           ..................C......................................................................................................................................
cenII39_2                                                                                           ..................C......................................................................................................................................
cenII39_2                                                                                           ..................C......................................................................................................................................
cenII39_2                                                                                           ..................C......................................................................................................................................
cenII39_2                                                                                           ..................C......................................................................................................................................
cenII39_2                                                                                           ..................C......................................................................................................................................
cenII39_2                                                                                           ..................C......................................................................................................................................
cenII39_1                                                                                                                                                                                                                         CAACTATCACACATCAACTGCAA.........................................................................................................................................
cenII39_1                                                                                                                                                                                                                                                .........................................................................................................................................
cenII39_1                                                                                                                                                                                                                                                .........................................................................................................................................
cenII39_1                                                                                                                                                                                                                                                .........................................................................................................................................
cenII39_1                                                                                                                                                                                                                                                ..............................T..........................................................................................................
cenII39_1                                                                                                                                                                                                                                                .........................................................................................................................................
cenII39_1                                                                                                                                                                                                                                                .........................................................................................................................................
cenII39_1                                                                                                                                                                                                                                                .........................................................................................................................................
cenII39_2                                                                                                                                                                                                                                                .........................................................................................................................................
cenII39_2                                                                                                                                                                                                                                                .........................................................................................................................................
cenII39_2                                                                                                                                                                                                                                                .........................................................................................................................................
cenII39_2                                                                                                                                                                                                                                                .........................................................................................................................................
cenII39_2                                                                                                                                                                                                                                                .........................................................................................................................................
cenII39_2                                                                                                                                                                                                                                                .........................................................................................................................................
cenII39_2                                                                                                                                                                                                                                                .........................................................................................................................................
cenII39_2                                                                                                                                                                                                                                                .........................................................................................................................................
cenII39_3                                       .............T........................................................C...........................................................................................
cenII39_3                                       ......................................................................C..........................................................................T................
cenII39_3                                       .............T........................................................C...........................................................................................
cenII39_3                                       .............T........................................................C...........................................................................................
cenII39_3                                       .............T........................................................C...........................................................................................
cenII39_3                                       ..................C....................T.............C...C..C.................................................................C.....C.............................
cenII39_3                                                                                                                                                                                         .....................................T..............................................................................................G......C.T..................................................
cenII39_3                                                                                                                                                                                         ......T.........................................................................................................................................................................................
cenII39_3                                                                                                                                                                                         .................................G..........................................................T...................................................................................................
cenII39_3                                                                                                                                                                                         ........................................................A............A..............A.......T....................C..G.......................G...................................................
cenII39_3                                                                                                                                                                                         .....................................T......................................................T...................................................................................................
cenII39_3                                                                                                                                                                                         ................................................................................................................................................................................................

cenII39 consensus
                TTCTTTCATGGGGAAGCAGATTTGGGTACCACCCAAGTATTGACTTACCCATCAACAACCGCTATGTATTTCGTACATTACTGCCAGCCACCATGAATATTGCACGGTACCATAAATACTTGACCACCTGTAGTACATAAAAACCCAATCCACATCAAAACCCCCTCCCCATGCTTACAAGCAAGTACAGCAATCAACCCTCAACTATCACACATCAACTGCAACTCCAAAGCCACCCCTCACCCACTAGGATACCAACAAACCTACCCACCCTTAACAGTACATAGTACATAAAGCCATTTACCGTACATAGCACATTACAGTCAAATCCCTTCTCGTCCCCATGGATGACCCCCCTCAG
```
