# Supplementary material for: Genealogical Relationships between Early Medieval and Modern Inhabitants of Piedmont
Source: PLoS One. 2015 Jan 30;10(1):e0116801. doi: 10.1371/journal.pone.0116801 (PMC4312042; doi:10.1371/journal.pone.0116801)
Supplement: S2 Fig — (PDF) [file pone.0116801.s002.pdf]

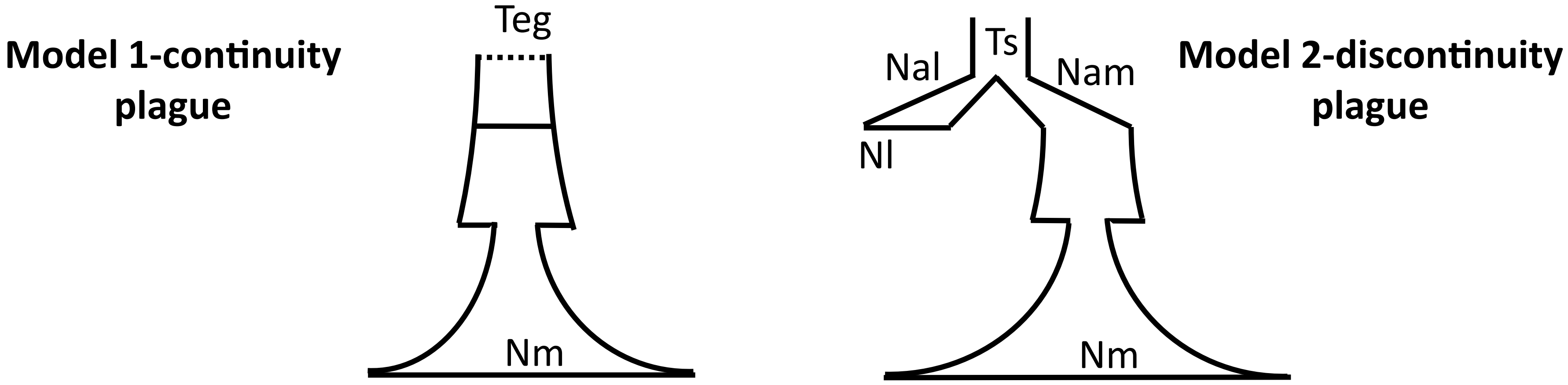

Bottleneck size: {U:100,10000}
Bottleneck Intensity: {U:1.1,10}
Bottleneck Time: {U:1,400}
