# Supplementary figures and images for: Genealogical Relationships between Early Medieval and Modern Inhabitants of Piedmont
Source: PLoS One. 2015 Jan 30;10(1):e0116801. doi: 10.1371/journal.pone.0116801 (PMC4312042; doi:10.1371/journal.pone.0116801)

MDS plot

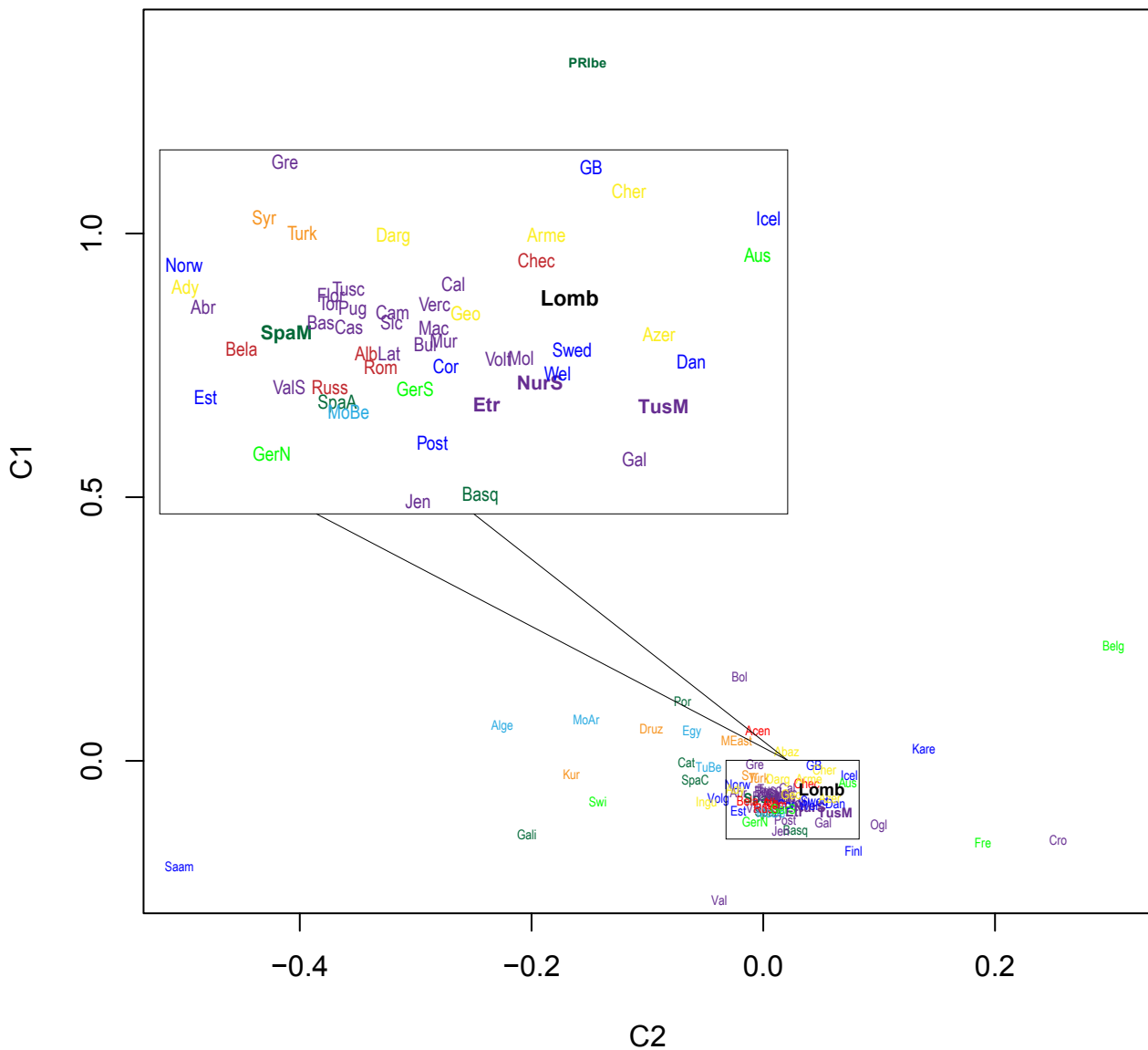

Supplement: S3 Fig — Populations are labelled as reported in S2 Table. (PDF) [file pone.0116801.s003.pdf]

A

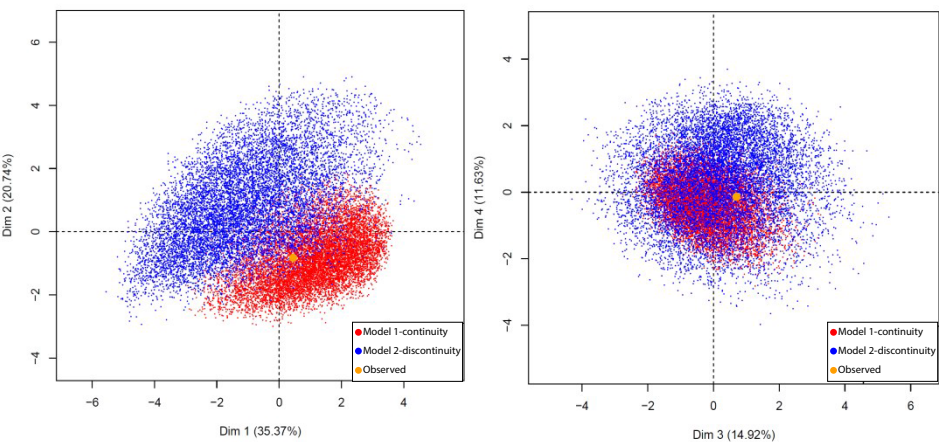

B

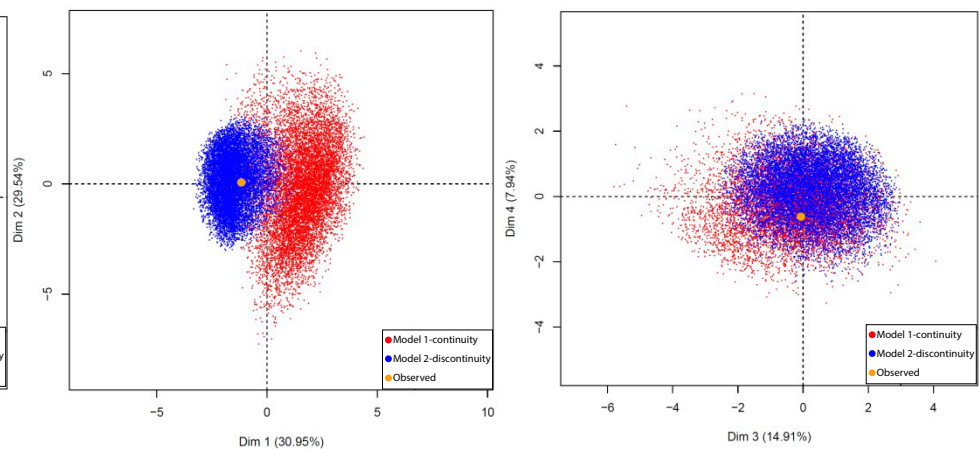

C

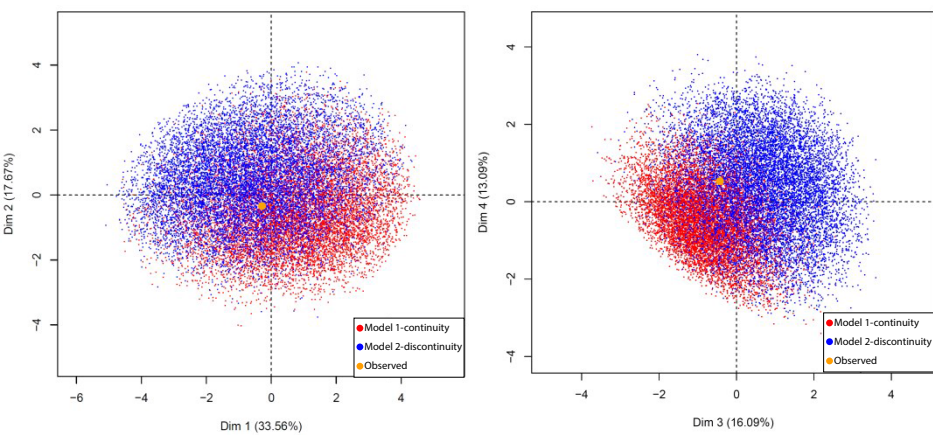

D

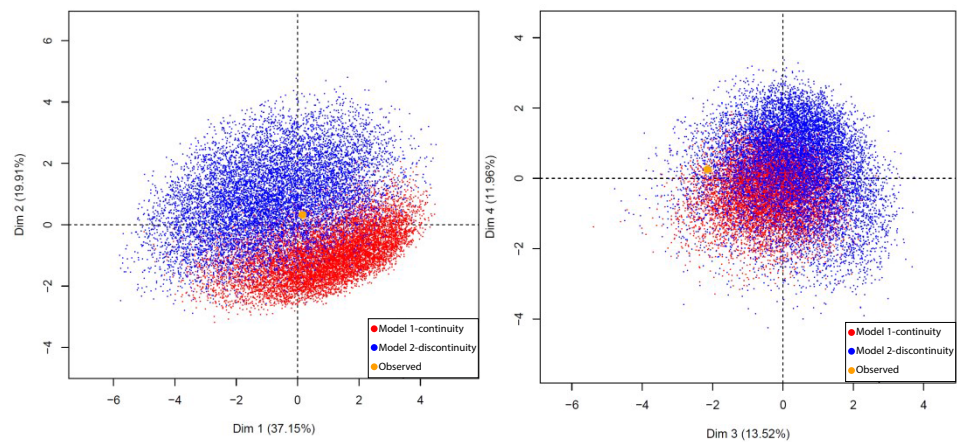

Supplement: S4 Fig — A: Trino Vercellese; B: Postua; C: Val di Susa; D: Turin. (PDF) [file pone.0116801.s004.pdf]
