# Supplementary material for: Genealogical Relationships between Early Medieval and Modern Inhabitants of Piedmont
Source: PLoS One. 2015 Jan 30;10(1):e0116801. doi: 10.1371/journal.pone.0116801 (PMC4312042; doi:10.1371/journal.pone.0116801)
Supplement: S1 Table — (DOC) [file pone.0116801.s006.doc]

**Table S1. Control-region haplotypes and haplogroup/sub-haplogroup classification of the 222 modern Piedmontese mtDNAs from Trino Vercellese, Postua and Val di Susa.**

| **Sample ID** | **GenBank Accession Number** | **Origin of Sample** | **Start of Sequence Reading (np)** | **End of Sequence Reading (np)** | **Haplotype** | | **Haplogroup** |
| --- | --- | --- | --- | --- | --- | --- | --- |
|  |  |  |  |  | **From Start of Reading till np 16569**  **(-16000)^a^** | **From np 1 to the End of Sequence Reading^a^** |  |
| TV 1 | KP151653 | Trino Vercellese | 16024 | 200 | 126 294 296 519 | 73 | **T2** |
| TV 3 | KP151654 | Trino Vercellese | 16024 | 210 | 126 140 189 294 296 311 519 | 73 | **T2** |
| TV 4 | KP151655 | Trino Vercellese | 16024 | 200 | CRS | CRS | **H** |
| TV 5 | KP151656 | Trino Vercellese | 16024 | 200 | 129 134 356 519 | 73 152 195 | **U4** |
| TV 7 | KP151657 | Trino Vercellese | 16024 | 210 | 051 519 | 151 152 | **H** |
| TV 9 | KP151658 | Trino Vercellese | 16024 | 200 | 093 293 311 | 195 | **H1** |
| TV 10 | KP151659 | Trino Vercellese | 16024 | 210 | 104 343 519 | 73 146 150 | **U3** |
| TV 11 | KP151660 | Trino Vercellese | 16024 | 210 | 519 | CRS | **H1** |
| TV 13 | KP151661 | Trino Vercellese | 16024 | 200 | 069 126 193 278 297 360 | 73 150 152 189 | **J2** |
| TV 14 | KP151662 | Trino Vercellese | 16024 | 210 | 291 519 | CRS | **H1** |
| TV 17 | KP151663 | Trino Vercellese | 16024 | 120 | 362 519 | CRS | **H** |
| TV 19 | KP151664 | Trino Vercellese | 16024 | 210 | 294 304 | CRS | **H5** |
| TV 20 | KP151665 | Trino Vercellese | 16024 | 200 | 362 519 | CRS | **H** |
| TV 23 | KP151666 | Trino Vercellese | 16024 | 200 | 174 519 | CRS | **H1** |
| TV 24 | KP151667 | Trino Vercellese | 16024 | 200 | 293 311 519 | 195 | **H** |
| TV 25 | KP151668 | Trino Vercellese | 16024 | 250 | 224 311 519 | 73 | **K1** |
| TV 26 | KP151669 | Trino Vercellese | 16024 | 200 | 519 | 195 | **H** |
| TV 27 | KP151670 | Trino Vercellese | 16024 | 200 | 304 | 152 | **H5** |
| TV 30 | KP151671 | Trino Vercellese | 16024 | 200 | 519 | 194 | **H3** |
| TV 31 | KP151672 | Trino Vercellese | 16024 | 200 | 069 126 145 172 222 224 261 | 73 91d | **J1** |
| TV 32 | KP151673 | Trino Vercellese | 16024 | 120 | 291 519 | 73 | **H1** |
| TV 33 | KP151674 | Trino Vercellese | 16024 | 250 | 069 126 | 73 228 | **J1** |
| TV 35 | KP151675 | Trino Vercellese | 16024 | 210 | 126 294 304 519 | 73 | **T2** |
| TV 36 | KP151676 | Trino Vercellese | 16024 | 250 | 519 | 195 | **H** |
| TV 37 | KP151677 | Trino Vercellese | 16024 | 200 | 037 224 234 311 519 | 73 146 150 | **K2** |
| TV 38 | KP151678 | Trino Vercellese | 16024 | 250 | 069 126 | 73 174A | **J1** |
| TV 39 | KP151679 | Trino Vercellese | 16024 | 200 | 069 126 145 172 222 261 | 73 | **J1** |
| TV 40 | KP151680 | Trino Vercellese | 16024 | 200 | 519 | 73 | **H** |
| TV 41 | KP151681 | Trino Vercellese | 16024 | 240 | 145 362 482 | 239 | **H6** |
| TV 46 | KP151682 | Trino Vercellese | 16024 | 210 | 126 135+G 294 296 304 519 | 73 91d | **T2** |
| TV 47 | KP151683 | Trino Vercellese | 16024 | 250 | 129 223 391 519 | 73 152 199 204 250 | **I** |
| TV 48 | KP151684 | Trino Vercellese | 16024 | 250 | 311 362 | CRS | **HV** |
| TV 50 | KP151685 | Trino Vercellese | 16024 | 250 | 192 223 286 292 325 519 | 73 150A 189 195 204 | **W** |
| TV 51 | KP151686 | Trino Vercellese | 16024 | 250 | 126 | 195 | **H** |
| TV 55 | KP151687 | Trino Vercellese | 16024 | 210 | 104 343 519 | 73 146 150 | **U3** |
| TV 57 | KP151688 | Trino Vercellese | 16024 | 250 | 126 298 346C | 72 200 207 | **HV0a** |
| TV 58 | KP151689 | Trino Vercellese | 16024 | 210 | 104 343 519 | 73 146 150 | **U3** |
| TV 60 | KP151690 | Trino Vercellese | 16024 | 210 | 261 519 | CRS | **H** |
| TV 61 | KP151691 | Trino Vercellese | 16024 | 200 | 092 183C 189 278 519 | 73 153 | **X** |
| TV 63 | KP151692 | Trino Vercellese | 16024 | 200 | 214 224 297A 311 354 497 519 | 73 152 | **K1** |
| TV 65 | KP151693 | Trino Vercellese | 16024 | 250 | 145 362 482 | 239 | **H6** |
| TV 70 | KP151694 | Trino Vercellese | 16024 | 210 | 182C 183C 189 223 278 519 | 73 152 195 | **X2** |
| TV 71 | KP151695 | Trino Vercellese | 16024 | 250 | 104 343 519 | 73 146 150 | **U3** |
| TV 72 | KP151696 | Trino Vercellese | 16024 | 250 | 126 298 346C | 72 200 207 | **HV0a** |
| TV 75 | KP151697 | Trino Vercellese | 16024 | 250 | 069 362 | 73 228 | **J1** |
| TV 76 | KP151698 | Trino Vercellese | 16024 | 200 | 126 294 304 519 | 73 | **T2** |
| TV 78 | KP151699 | Trino Vercellese | 16024 | 200 | 362 519 | CRS | **H3** |
| TV 79 | KP151700 | Trino Vercellese | 16024 | 250 | 093 221 266 519 | CRS | **H** |
| TV 81 | KP151701 | Trino Vercellese | 16024 | 200 | 093 293 311 | 195 | **H** |
| TV 83 | KP151702 | Trino Vercellese | 16024 | 250 | 093 519 | CRS | **H1** |
| TV 84 | KP151703 | Trino Vercellese | 16024 | 250 | 069 126 319 | 73 | **J1** |
| TV 85 | KP151704 | Trino Vercellese | 16024 | 250 | 129 148 223 301 391 519 | 73 199 204 250 | **I** |
| TV 88 | KP151705 | Trino Vercellese | 16024 | 210 | CRS | 152 | **H** |
| TV 89 | KP151706 | Trino Vercellese | 16024 | 250 | 218 298 519 | 72 194 | **V** |
| TV 92 | KP151707 | Trino Vercellese | 16024 | 200 | 293 311 519 | 195 | **H** |
| TV 93 | KP151708 | Trino Vercellese | 16024 | 200 | 519 | CRS | **HV** |
| TV 94 | KP151709 | Trino Vercellese | 16024 | 120 | 519 | CRS | **H1** |
| TV 95 | KP151710 | Trino Vercellese | 16024 | 120 | 311 519 | CRS | **H** |
| TV 96 | KP151711 | Trino Vercellese | 16024 | 120 | 093 129 223 391 519 | 73 | **I** |
| TV 97 | KP151712 | Trino Vercellese | 16024 | 120 | 126 265 294 296 304 519 | 73 | **T2** |
| TV 99 | KP151713 | Trino Vercellese | 16024 | 200 | 126 140 189 294 296 311 519 | 73 | **T2** |
| TV 100 | KP151714 | Trino Vercellese | 16024 | 120 | 519 | CRS | **H3** |
| TV 104 | KP151715 | Trino Vercellese | 16024 | 200 | 304 390 519 | CRS | **H5** |
| TV 106 | KP151716 | Trino Vercellese | 16024 | 120 | 126 265 294 296 304 519 | 73 | **T2** |
| TV 108 | KP151717 | Trino Vercellese | 16024 | 120 | 284 355 519 | CRS | **H1** |
| TV 110 | KP151718 | Trino Vercellese | 16024 | 200 | 362 519 | CRS | **H3** |
| TV 113 | KP151719 | Trino Vercellese | 16024 | 240 | 280 362 482 | 239 | **H6** |
| TV 114 | KP151720 | Trino Vercellese | 16024 | 120 | 069 126 145 172 222 261 | 73 | **J1** |
| TV 116 | KP151721 | Trino Vercellese | 16024 | 200 | 519 | CRS | **H** |
| TV 120 | KP151722 | Trino Vercellese | 16024 | 120 | 146 342 | 73 | **U8** |
| TV 121 | KP151723 | Trino Vercellese | 16024 | 200 | 278 293 311 | 195 | **H** |
| TV 128 | KP151724 | Trino Vercellese | 16024 | 210 | 126 182C 183C 189 294 296 298 519 | 73 195 | **T2** |
| TV 129 | KP151725 | Trino Vercellese | 16024 | 210 | 192 222 256 270 291 399 526 | 73 | **U5a** |
| TV 131 | KP151726 | Trino Vercellese | 16024 | 250 | 224 304 311 519 | 73 150 231d | **K1** |
| TV 132 | KP151727 | Trino Vercellese | 16024 | 210 | 093 189 270 | 73 150 | **U5b** |
| PT 1 | KP151728 | Postua | 16024 | 250 | CRS | CRS | **H** |
| PT 2 | KP151729 | Postua | 16024 | 263 | 220 519 | 195 263 | **H** |
| PT 3 | KP151730 | Postua | 16024 | 263 | 220 519 | 195 263 | **H** |
| PT 4 | KP151731 | Postua | 16024 | 263 | 519 | 73 263 | **H** |
| PT 5 | KP151732 | Postua | 16024 | 263 | 519 | 73 263 | **H** |
| PT 7 | KP151733 | Postua | 16024 | 263 | 224 311 519 | 73 146 152 263 | **K2** |
| PT 8 | KP151734 | Postua | 16024 | 263 | 298 | 72 263 | **V** |
| PT 9 | KP151735 | Postua | 16024 | 250 | 172 519 | 44+C 207 | **H** |
| PT 10 | KP151736 | Postua | 16024 | 263 | 298 | 72 263 | **V** |
| PT 11 | KP151737 | Postua | 16024 | 263 | 069 126 145 172 222 261 | 73 146 242 263 | **J1** |
| PT 12 | KP151738 | Postua | 16024 | 250 | 304 519 | CRS | **H5** |
| PT 13 | KP151739 | Postua | 16024 | 250 | 304 | 152 | **H5** |
| PT 14 | KP151740 | Postua | 16024 | 263 | 519 | 146 152 263 | **H3** |
| PT 15 | KP151741 | Postua | 16024 | 263 | 519 | 146 263 | **H3** |
| PT 16 | KP151742 | Postua | 16024 | 263 | 304 | 152 263 | **H5** |
| PT 17 | KP151743 | Postua | 16024 | 250 | 051 129C 183C 189 311 362 519 | 73 152 217 | **U2** |
| PT 18 | KP151744 | Postua | 16024 | 263 | 519 | 263 | **H1** |
| PT 19 | KP151745 | Postua | 16024 | 263 | 249 519 | 152 263 | **H3** |
| PT 20 | KP151746 | Postua | 16024 | 263 | 304 519 | 152 263 | **H5** |
| PT 21 | KP151747 | Postua | 16024 | 250 | 224 311 519 | 73 146 152 | **K2** |
| PT 22 | KP151748 | Postua | 16024 | 263 | 162 172 209 519 | 73 263 | **H1** |
| PT 23 | KP151749 | Postua | 16024 | 263 | 069 126 220T 278 | 73 185h 263 | **J1** |
| PT 24 | KP151750 | Postua | 16024 | 263 | CRS | 262 263 | **H** |
| PT 25 | KP151751 | Postua | 16024 | 263 | 270 519 | 152 263 | **H1** |
| PT 26 | KP151752 | Postua | 16024 | 263 | 224 311 519 | 73 146 152 263 | **K2** |
| PT 27 | KP151753 | Postua | 16024 | 200 | 224 311 519 | 73 146 152 | **K2** |
| PT 28 | KP151754 | Postua | 16024 | 263 | 298 | 72 263 | **V** |
| PT 29 | KP151755 | Postua | 16024 | 263 | 519 | 73 263 | **H** |
| PT 30 | KP151756 | Postua | 16024 | 263 | 519 | 263 | **H1** |
| PT 31 | KP151757 | Postua | 16024 | 263 | 172 519 | 44+C 207 263 | **H** |
| PT 33 | KP151758 | Postua | 16024 | 263 | 519 | 143 263 | **H** |
| PT 34 | KP151759 | Postua | 16024 | 250 | 069 126 145 261 519 | 73 | **J1** |
| PT 35 | KP151760 | Postua | 16024 | 263 | 126 163 186 189 294 519 | 9 73 152 195 263 | **T1** |
| PT 36 | KP151761 | Postua | 16024 | 210 | 519 | 143 152 | **H** |
| PT 37 | KP151762 | Postua | 16024 | 200 | 224 311 519 | 73 146 | **K1** |
| PT 38 | KP151763 | Postua | 16024 | 250 | 189 519 | CRS | **H** |
| PT 39 | KP151764 | Postua | 16024 | 263 | 519 | 146 152 263 | **H** |
| PT 40 | KP151765 | Postua | 16024 | 263 | 298 | 263 | **V** |
| PT 41 | KP151766 | Postua | 16024 | 250 | 051 129C 182C 183C 189 256 362 | 73 152 217 | **U2** |
| PT 42 | KP151767 | Postua | 16024 | 250 | 126 224 311 519 | 73 146 195 | **K1** |
| PT 43 | KP151768 | Postua | 16024 | 250 | 069 126 145 261 519 | 73 | **J1** |
| PT 44 | KP151769 | Postua | 16024 | 210 | 519 | CRS | **H1** |
| PT 45 | KP151770 | Postua | 16024 | 210 | 189 298 | 72 | **V** |
| PT 46 | KP151771 | Postua | 16024 | 250 | 220 519 | 195 | **H** |
| PT 47 | KP151772 | Postua | 16024 | 250 | 189 215 218+T 519 | CRS | **H** |
| PT 48 | KP151773 | Postua | 16024 | 250 | 183C 189 519 | 153 204 207 | **H** |
| PT 49 | KP151774 | Postua | 16024 | 250 | 183C 189 519 | 153 204 207 | **H** |
| PT 50 | KP151775 | Postua | 16024 | 263 | 519 | 73 263 | **H** |
| PT 51 | KP151776 | Postua | 16024 | 263 | 519 | 73 263 | **H** |
| PT 52 | KP151777 | Postua | 16024 | 120 | 192 354 | CRS | **H** |
| PT 53 | KP151778 | Postua | 16024 | 250 | 092 189 298 | 72 152 195 | **HV0*** |
| PT 54 | KP151779 | Postua | 16024 | 263 | 304 519 | 263 | **H5** |
| PT 55 | KP151780 | Postua | 16024 | 263 | 304 519 | 263 | **H5** |
| PT 56 | KP151781 | Postua | 16024 | 263 | 304 519 | 263 | **H5** |
| PT 57 | KP151782 | Postua | 16024 | 263 | 192 354 | 263 | **H** |
| PT 58 | KP151783 | Postua | 16024 | 263 | 192 354 | 263 | **H** |
| PT 59 | KP151784 | Postua | 16024 | 263 | 223 292 519 | 73 189 194 195 207 263 | **W** |
| PT 60 | KP151785 | Postua | 16024 | 263 | 224 311 519 | 73 146 152 263 | **K2** |
| PT 61 | KP151786 | Postua | 16024 | 250 | 129 189 223 278 519 | 73 153 195 225 226 | **X2** |
| PT 62 | KP151787 | Postua | 16024 | 250 | 183C 189 223 278 519 | 73 153 195 225 226 | **X2** |
| PT 63 | KP151788 | Postua | 16024 | 250 | 183C 189 223 278 519 | 73 153 195 225 226 | **X2** |
| PT 65 | KP151789 | Postua | 16024 | 263 | 126 147 266 274 294 296 297 304 354 519 | 73 263 | **T2** |
| PT 66 | KP151790 | Postua | 16024 | 263 | 304 519 | 263 | **H5** |
| PT 68 | KP151791 | Postua | 16024 | 263 | 304 519 | 263 | **H5** |
| PT 69 | KP151792 | Postua | 16024 | 263 | 179 356 519 | 73 150 195 263 | **U4** |
| PT 70 | KP151793 | Postua | 16024 | 200 | 179 356 519 | 73 150 195 | **U4** |
| PT 71 | KP151794 | Postua | 16024 | 263 | CRS | 263 | **H** |
| PT 72 | KP151795 | Postua | 16024 | 250 | 172 519 | 44+C 207 | **H** |
| PT 76 | KP151796 | Postua | 16024 | 250 | 051 129C 182C 183C 189 256 362 | 73 152 217 | **U2** |
| PT 77 | KP151797 | Postua | 16024 | 200 | 223 292 519 | 73 189 194 195 | **W** |
| PT 79 | KP151798 | Postua | 16024 | 263 | 270 519 | 152 263 | **H1** |
| PT 80 | KP151799 | Postua | 16024 | 250 | 172 519 | 44+C 207 | **H** |
| PT 81 | KP151800 | Postua | 16024 | 250 | 519 | CRS | **H** |
| PT 83 | KP151801 | Postua | 16024 | 210 | 172 519 | 44+C 207 | **H** |
| PT 86 | KP151802 | Postua | 16024 | 210 | 519 | CRS | **H1** |
| PT 89 | KP151803 | Postua | 16024 | 120 | 309 519 | CRS | **H1** |
| PT 90 | KP151804 | Postua | 16024 | 120 | 519 | CRS | **H** |
| PT 91 | KP151805 | Postua | 16024 | 210 | 304 519 | CRS | **H5** |
| PT 92 | KP151806 | Postua | 16024 | 210 | 304 519 | CRS | **H5** |
| PT 93 | KP151807 | Postua | 16024 | 250 | 224 311 519 | 73 195 | **K1** |
| PT 94 | KP151808 | Postua | 16024 | 263 | 304 519 | 263 | **H5** |
| PT 95 | KP151809 | Postua | 16024 | 263 | 519 | 93 263 | **H** |
| PT 96 | KP151810 | Postua | 16024 | 210 | 298 | 72 | **V** |
| PT 97 | KP151811 | Postua | 16024 | 200 | 304 519 | CRS | **H5** |
| PT 98 | KP151812 | Postua | 16024 | 263 | 304 519 | 263 | **H5** |
| PT 99 | KP151813 | Postua | 16024 | 263 | 304 519 | 263 | **H5** |
| PT 100 | KP151814 | Postua | 16024 | 120 | 519 | CRS | **H3** |
| PT 101 | KP151815 | Postua | 16024 | 263 | 270 519 | 152 263 | **H1** |
| PT 102 | KP151816 | Postua | 16024 | 200 | 298 | 72 | **V** |
| VDS 1 | KP151817 | Val di Susa | 16024 | 300 | 038 129 223 391 519 | 73 152 199 204 207 250 263 | **I** |
| VDS 2 | KP151818 | Val di Susa | 16024 | 210 | 172 189 519 | 207 | **H** |
| VDS 3 | KP151819 | Val di Susa | 16024 | 210 | 189 519 | 207 | **H** |
| VDS 4 | KP151820 | Val di Susa | 16024 | 300 | 051 162 519 | 73 263 | **H1** |
| VDS 5 | KP151821 | Val di Susa | 16024 | 300 | 224 311 519 | 73 263 | **K1** |
| VDS 6 | KP151822 | Val di Susa | 16024 | 300 | CRS | 73 111 263 | **H** |
| VDS 7 | KP151823 | Val di Susa | 16024 | 300 | 311 519 | 263 | **H** |
| VDS 8 | KP151824 | Val di Susa | 16024 | 300 | CRS | 73 111 263 | **H** |
| VDS 9 | KP151825 | Val di Susa | 16024 | 210 | 189 519 | 207 | **H** |
| VDS 10 | KP151826 | Val di Susa | 16024 | 210 | 189 224 270 362 519 | 73 150 | **U5b** |
| VDS 11 | KP151827 | Val di Susa | 16024 | 300 | 063 069 126 | 73 189 228 234 263 295 | **J1** |
| VDS 12 | KP151828 | Val di Susa | 16024 | 300 | 283 519 | 263 | **H** |
| VDS 13 | KP151829 | Val di Susa | 16024 | 300 | 111A 222 519 | 263 | **H** |
| VDS 14 | KP151830 | Val di Susa | 16024 | 300 | 114A 192 256 270 294 304 526 | 73 263 | **U5a** |
| VDS 16 | KP151831 | Val di Susa | 16014 | 210 | 093 519 | CRS | **H** |
| VDS 17 | KP151832 | Val di Susa | 16024 | 250 | 129 519 | CRS | **H1** |
| VDS 18 | KP151833 | Val di Susa | 16024 | 300 | 362 482 | 239 263 | **H6** |
| VDS 19 | KP151834 | Val di Susa | 16024 | 210 | 145 172 189 243 256 270 399 | 73 195 | **U5a** |
| VDS 20 | KP151835 | Val di Susa | 16024 | 210 | 189 270 | 73 150 | **U5b** |
| VDS 21 | KP151836 | Val di Susa | 16024 | 300 | 051 519 | 152 263 | **H1** |
| VDS 22 | KP151837 | Val di Susa | 16024 | 300 | 129 519 | 263 | **H1** |
| VDS 23 | KP151838 | Val di Susa | 16024 | 300 | 129 519 | 263 | **H1** |
| VDS 24 | KP151839 | Val di Susa | 16024 | 210 | 183C 189 519 | 207 | **H** |
| VDS 25 | KP151840 | Val di Susa | 16024 | 300 | 192 256 270 399 | 73 263 | **U5a** |
| VDS 26 | KP151841 | Val di Susa | 16024 | 300 | 519 | 263 | **H1** |
| VDS 27 | KP151842 | Val di Susa | 16024 | 300 | 093 519 | 263 | **H** |
| VDS 28 | KP151843 | Val di Susa | 16024 | 300 | 126 145 187 294 296 304 519 | 73 151 195 263 | **T2** |
| VDS 29 | KP151844 | Val di Susa | 16024 | 300 | 187A 294 | 146 263 | **HV** |
| VDS 30 | KP151845 | Val di Susa | 16024 | 300 | CRS | 73 111 263 | **H** |
| VDS 31 | KP151846 | Val di Susa | 16024 | 300 | 311 519 | 263 | **H1** |
| VDS 32 | KP151847 | Val di Susa | 16024 | 300 | 126 145 187 294 296 304 519 | 73 151 195 263 | **T2** |
| VDS 33 | KP151848 | Val di Susa | 16024 | 300 | 224 311 519 | 73 263 | **K1** |
| VDS 34 | KP151849 | Val di Susa | 16024 | 300 | 162 519 | 73 263 | **H1** |
| VDS 35 | KP151850 | Val di Susa | 16024 | 300 | 519 | 263 | **H1** |
| VDS 36 | KP151851 | Val di Susa | 16024 | 263 | 126 163 186 189 294 519 | 73 152 195 263 | **T1** |
| VDS 37 | KP151852 | Val di Susa | 16024 | 300 | 126 163 186 189 294 519 | 73 152 195 263 | **T1** |
| VDS 38 | KP151853 | Val di Susa | 16024 | 300 | 311 519 | 263 | **H3** |
| VDS 39 | KP151854 | Val di Susa | 16024 | 263 | CRS | 73 111 263 | **H** |
| VDS 40 | KP151855 | Val di Susa | 16024 | 263 | 069 093 126 187 189 192 | 73 185 235 263 | **J1** |
| VDS 41 | KP151856 | Val di Susa | 16024 | 300 | 519 | 263 | **H1** |
| VDS 42 | KP151857 | Val di Susa | 16024 | 300 | 051 162 519 | 73 263 | **H1** |
| VDS 43 | KP151858 | Val di Susa | 16024 | 300 | 192 270 | 73 150 152 263 | **U5b** |
| VDS 44 | KP151859 | Val di Susa | 16024 | 300 | 239 298 | 72 263 | **V** |
| VDS 45 | KP151860 | Val di Susa | 16024 | 300 | 069 126 | 73 185 228 263 295 | **J1** |
| VDS 46 | KP151861 | Val di Susa | 16024 | 300 | 224 270 362 519 | 73 150 263 | **U5b** |
| VDS 47 | KP151862 | Val di Susa | 16024 | 300 | 111 357 519 | 263 | **H1** |
| VDS 49 | KP151863 | Val di Susa | 16024 | 300 | 304 362 | 263 | **H5** |
| VDS 50 | KP151864 | Val di Susa | 16024 | 210 | 189 519 | 207 | **H** |
| VDS 51 | KP151865 | Val di Susa | 16024 | 300 | CRS | 73 111 263 | **H** |
| VDS 52 | KP151866 | Val di Susa | 16024 | 300 | 235 311 | 263 | **H** |
| VDS 53 | KP151867 | Val di Susa | 16024 | 300 | 129 519 | 263 | **H1** |
| VDS 54 | KP151868 | Val di Susa | 16024 | 210 | 189 519 | 207 | **H** |
| VDS 55 | KP151869 | Val di Susa | 16024 | 300 | 239 298 | 72 263 | **V** |
| VDS 56 | KP151870 | Val di Susa | 16024 | 300 | 298 | 72 263 | **V** |
| VDS 57 | KP151871 | Val di Susa | 16024 | 210 | 189 519 | 207 | **H** |
| VDS 58 | KP151872 | Val di Susa | 16024 | 300 | 224 311 519 | 73 263 | **K1** |
| VDS 59 | KP151873 | Val di Susa | 16024 | 210 | 189 519 | CRS | **H** |
| VDS 60 | KP151874 | Val di Susa | 16024 | 210 | 239 298 | 72 | **V** |

^a^ Mutations are transitions relative to the CRS unless a base is explicitly indicated, while suffixes indicate: transversions (to A, G, C, or T), indels (+, d) and heteroplasmy (h).
