# Supplementary material for: Genealogical Relationships between Early Medieval and Modern Inhabitants of Piedmont
Source: PLoS One. 2015 Jan 30;10(1):e0116801. doi: 10.1371/journal.pone.0116801 (PMC4312042; doi:10.1371/journal.pone.0116801)
Supplement: S2 Table — Sample size (n), gene diversity and Mean Pairwise Sequence Difference (MPWD) are given. (DOC) [file pone.0116801.s007.doc]

**Table S2. Additional contemporary samples considered in the analyses. Sample size (n).** gene diversity and Mean Pairwise Sequence Difference (MPWD) are given.

| **Population** | **ID** | **Region** | **n** | **Reference** | **Gene Diversity** | **MPWD** |
| --- | --- | --- | --- | --- | --- | --- |
| Abazinian | Abaz | Caucasus | 23 | [1] | 0.980 | 4.696 |
| Adygei | Ady | Caucasus | 50 | [2] | 0.953 | 4.485 |
| Albanians | Alb | East Europe | 84 | [3,4] | 0.966 | 3.775 |
| Algerians | Alge | North Africa | 85 | [5] | 0.943 | 4.594 |
| Arabs. Maroc | MoAr | North Africa | 32 | [6] | 0.988 | 5.726 |
| Armenians | Arme | Caucasus | 42 | [7] | 0.973 | 4.645 |
| Austrians | Aus | Central Europe | 117 | [7.8] | 0.958 | 4.300 |
| Azerbaijani | Azer | Caucasus | 41 | [1] | 0.995 | 4.651 |
| Basques | Basq | West Europe | 106 | [5,9] | 0.932 | 2.894 |
| Belarus | Bela | East Europe | 55 | [10] | 0.997 | 4.564 |
| Belgians | Belg | Central Europe | 33 | [11] | 0.987 | 3.055 |
| Berbers. Maroc | MoBe | North Africa | 60 | [6,12] | 0.963 | 4.345 |
| Berbers. Tunisia | TuBe | North Africa | 155 | [13] | 0.972 | 6.067 |
| British | GB | North Europe | 100 | [14] | 0.976 | 4.279 |
| Bulgarians | Bul | South Europe | 882 | [15,16] | 0.981 | 4.325 |
| Catalans | Cat | West Europe | 15 | [5] | 0.952 | 3.352 |
| Chechenians | Chec | East Europe | 23 | [1] | 0.961 | 4.229 |
| Cherkessians | Cher | Caucasus | 44 | [1] | 0.984 | 4.830 |
| Cornish | Cor | North Europe | 69 | [17] | 0.963 | 3.614 |
| Croatians | Cro | South Europe | 96 | [18] | 0.973 | 3.526 |
| Danes | Dan | North Europe | 32 | [17] | 0.934 | 3.234 |
| Darginians | Darg | Caucasus | 37 | [1] | 0.973 | 4.477 |
| Druze | Druz | Near East | 45 | [2] | 0.957 | 4.648 |
| Egyptians | Egy | North Africa | 124 | [19,20] | 0.995 | 7.004 |
| Estonians | Est | North Europe | 28 | [21] | 0.976 | 4.032 |
| Finland | Finl | North Europe | 79 | [17,21] | 0.970 | 3.520 |
| French | Fre | Central Europe | 161 | [22,23] | 0.967 | 3.703 |
| Galicians | Gali | West Europe | 92 | [24] | 0.923 | 2.985 |
| Georgians | Geo | Caucasus | 102 | [1,25] | 0.965 | 4.473 |
| Germans. North | GerN | Central Europe | 108 | [17] | 0.970 | 3.702 |
| Germans. South | GerS | Central Europe | 249 | [17,26] | 0.975 | 3.865 |
| **Population** | **ID** | **Region** | **n** | **Reference** | **Gene Diversity** | **MPWD** |
| Greeks | Gre | South Europe | 125 | [4,27,28] | 0.978 | 4.387 |
| Icelander | Icel | North Europe | 445 | [17,21,29] | 0.973 | 4.282 |
| Ingush | Ingu | Caucasus | 35 | [1] | 0.961 | 4.161 |
| Italians. Abruzzo | Abr | South Europe | 11 | [18] | 1000 | 4.509 |
| Italians. Basilicata | Bas | South Europe | 92 | [30] | 0.977 | 4.274 |
| Bologna | Bol | South Europe | 100 | [31] | 0.965 | 4.121 |
| Italians. Calabria | Cal | South Europe | 95 | [30] | 0.964 | 4.376 |
| Italians. Campania | Cam | South Europe | 48 | [18] | 0.980 | 4.191 |
| Italians. Casentino | Cas | South Europe | 122 | [32] | 0.977 | 4.105 |
| Italians. Florence | Flor | South Europe | 48 | [33] | 0.980 | 4.152 |
| Italians. Gallura | Gal | South Europe | 27 | [34] | 0.966 | 4.422 |
| Italians. Jenne | Jen | South Europe | 103 | [35] | 0.834 | 2.957 |
| Italians. Latium | Lat | South Europe | 52 | [18] | 0.934 | 3.527 |
| Molise | Mol | South Europe | 62 | [18] | 0.930 | 3.686 |
| Italians. Murlo | Mur | South Europe | 86 | [32] | 0.976 | 4.278 |
| Italians. Ogliastra | Ogl | South Europe | 175 | [36] | 0.785 | 2.487 |
| Puglia | Pug | South Europe | 26 | [18] | 0.991 | 4.360 |
| Italians. Sicily | Sic | South Europe | 154 | [30] | 0.978 | 4.490 |
| Torino | Tor | South Europe | 50 | [33] | 0.992 | 4.744 |
| Italians. Tuscany | Tusc | South Europe | 49 | [37] | 0.969 | 5.031 |
| Italians. Vallepietra | Val | South Europe | 21 | [35] | 0.871 | 4.124 |
| Italians. Volterra | Volt | South Europe | 114 | [32] | 0.955 | 3.850 |
| Karelians | Kare | North Europe | 83 | [21] | 0.962 | 3.654 |
| Kazakhs. Kirghizs.Uyghurs | Acen | East Europe | 205 | [38] | 0.989 | 5.812 |
| Kurds | Kur | Near East | 29 | [38] | 0.958 | 4.010 |
| Macedonians | Mac | South Europe | 37 | [4] | 0.970 | 3.865 |
| Middle East | MEast | Near East | 42 | [39] | 0.995 | 6.568 |
| Norwegians | Norw | North Europe | 30 | [40] | 0.954 | 3.126 |
| Portuguese | Por | West Europe | 54 | [5] | 0.924 | 3.347 |
| Romanians | Rom | East Europe | 105 | [4] | 0.973 | 4.223 |
| Russians | Russ | East Europe | 241 | [10,21,40,41] | 0.985 | 4.131 |
| Saami | Saam | North Europe | 240 | [21] | 0.798 | 3.393 |
| Andalusian | SpaA | West Europe | 108 | [42] | 0.960 | 4.022 |
| **Population** | **ID** | **Region** | **n** | **Reference** | **Gene Diversity** | **MPWD** |
| Spaniards. Central | SpaC | West Europe | 74 | [5,12] | 0.987 | 5.251 |
| Syrians | Syr | Near East | 49 | [28] | 0.998 | 5.558 |
| Sweden | Swed | North Europe | 32 | [21,40] | 0.988 | 4.212 |
| Swiss | Swi | Central Europe | 74 | [43] | 0.956 | 3.393 |
| Turks. Anatolia | Turk | Near East | 131 | [15,17,44,45,46] | 0.983 | 5.248 |
| Volga-Finnic | Volg | North Europe | 34 | [21] | 0.973 | 3.977 |
| Welsh | Wel | North Europe | 92 | [17] | 0.931 | 3.283 |
| PIemonte Vercelli | Verc | South Europe | 75 | Present work | 0.972 | 4.359 |
| Piemonte Postua | Post | South Europe | 89 | Present work | 0.905 | 2.794 |
| PIemonte Val di Susa | ValS | South Europe | 58 | Present work | 0.959 | 3.203 |
| Nuragic Sardians | NurS | South Europe | 23 | [47] | 0.838 | 1.304 |
| Pre-Roman Iberians | PRIbe | West Europe | 17 | [48] | 0.949 | 2.118 |
| Etruscans | Etr | South Europe | 30 | [49,50] | 0.943 | 2.966 |
| Spain Medieval | SpaM | West Europe | 61 | [42] | 0.942 | 3.421 |
| Medieval Tuscans | TusM | South Europe | 27 | [51] | 0.860 | 1.972 |

**References**

1. Nasidze I, Stoneking M (2001) Mitochondrial DNA variation and language replacements in the Caucasus. Proc Biol Sci 268: 1197-1206.

2. Macaulay V, Richards M, Hickey E, Vega E, Cruciani F, et al. (1999) The emerging tree of West Eurasian mtDNAs: a synthesis of control-region sequences and RFLPs. Am J Hum Genet 64: 232-249.

3. Belledi M, Poloni ES, Casalotti R, Conterio F, Mikerezi I, et al. (2000) Maternal and paternal lineages in Albania and the genetic structure of Indo-European populations. Eur J Hum Genet 8: 480-486.

4. Bosch E, Calafell F, Gonzalez-Neira A, Flaiz C, Mateu E, et al. (2006) Paternal and maternal lineages in the Balkans show a homogeneous landscape over linguistic barriers. except for the isolated Aromuns. Ann Hum Genet 70: 459-487.

5. Corte-Real HB, Macaulay VA, Richards MB, Hariti G, Issad MS, et al. (1996) Genetic diversity in the Iberian Peninsula determined from mitochondrial sequence analysis. Ann Hum Genet 60: 331-350.

6. Rando JC, Pinto F, Gonzalez AM, Hernandez M, Larruga JM, et al. (1998) Mitochondrial DNA analysis of northwest African populations reveals genetic exchanges with European. near-eastern. and sub-Saharan populations. Ann Hum Genet 62: 531-550.

7. Handt O, Richards M, Trommsdorff M, Kilger C, Simanainen J, et al. (1994) Molecular genetic analyses of the Tyrolean Ice Man. Science 264: 1775-1778.

8. Parson W, Parsons TJ, Scheithauer R, Holland MM (1998) Population data for 101 Austrian Caucasian mitochondrial DNA d-loop sequences: application of mtDNA sequence analysis to a forensic case. Int J Legal Med 111: 124-132.

9. Bertranpetit J, Sala J, Calafell F, Underhill PA, Moral P, et al. (1995) Human mitochondrial DNA variation and the origin of Basques. Ann Hum Genet 59: 63-81.

10. Belyaeva O, Bermisheva M, Khrunin A, Slominsky P, Bebyakova N, et al. (2003) Mitochondrial DNA variations in Russian and Belorussian populations. Hum Biol 75: 647-660.

11. Decorte R, Jehaes E, Xiao F, Cassiman J-J (1996) Genetic analysis of single hair shafts by automated sequence analysis of the mitochondrial d-loop region. Advances in Forensic Haemogenetics 6: 17-19.

12. Pinto F, Gonzalez AM, Hernandez M, Larruga JM, Cabrera VM (1996) Genetic relationship between the Canary Islanders and their African and Spanish ancestors inferred from mitochondrial DNA sequences. Ann Hum Genet 60: 321-330.

13. Fadhlaoui-Zid K, Plaza S, Calafell F, Ben Amor M, Comas D, et al. (2004) Mitochondrial DNA heterogeneity in Tunisian Berbers. Ann Hum Genet 68: 222-233.

14. Piercy R, Sullivan KM, Benson N, Gill P (1993) The application of mitochondrial DNA typing to the study of white Caucasian genetic identification. Int J Legal Med 106: 85-90.

15. Calafell F, Underhill P, Tolun A, Angelicheva D, Kalaydjieva L (1996) From Asia to Europe: mitochondrial DNA sequence variability in Bulgarians and Turks. Ann Hum Genet 60: 35-49.

16. Karachanak S, Carossa V, Nesheva D, Olivieri A, Pala M, et al. (2011) Bulgarians vs the other European populations: a mitochondrial DNA perspective. Int J Legal Med.

17. Richards M, Corte-Real H, Forster P, Macaulay V, Wilkinson-Herbots H, et al. (1996) Paleolithic and neolithic lineages in the European mitochondrial gene pool. Am J Hum Genet 59: 185-203.

18. Babalini C, Martinez-Labarga C, Tolk HV, Kivisild T, Giampaolo R, et al. (2005) The population history of the Croatian linguistic minority of Molise (southern Italy): a maternal view. Eur J Hum Genet 13: 902-912.

19. Krings M, Salem AE, Bauer K, Geisert H, Malek AK, et al. (1999) mtDNA analysis of Nile River Valley populations: A genetic corridor or a barrier to migration? Am J Hum Genet 64: 1166-1176.

20. Stevanovitch A, Gilles A, Bouzaid E, Kefi R, Paris F, et al. (2004) Mitochondrial DNA sequence diversity in a sedentary population from Egypt. Ann Hum Genet 68: 23-39.

21. Sajantila A, Paabo S (1995) Language replacement in Scandinavia. Nat Genet 11: 359-360.

22. Rousselet F, Mangin P (1998) Mitochondrial DNA polymorphisms: a study of 50 French Caucasian individuals and application to forensic casework. Int J Legal Med 111: 292-298.

23. Cali F, Le Roux M, D'Anna R, Flugy A, De Leo G, et al. (2001) MtDNA control region and RFLP data for Sicily and France. Int J Legal Med 114: 229-231.

24. Salas A, Comas D, Lareu MV, Bertranpetit J, Carracedo A (1998) mtDNA analysis of the Galician population: a genetic edge of European variation. Eur J Hum Genet 6: 365-375.

25. Comas D, Calafell F, Bendukidze N, Fananas L, Bertranpetit J (2000) Georgian and kurd mtDNA sequence analysis shows a lack of correlation between languages and female genetic lineages. Am J Phys Anthropol 112: 5-16.

26. Lutz W, Scherbov S (1998) An expert-based framework for probabilistic national population projections: the example of Austria. Eur J Popul 14: 1-17.

27. Kouvatsi A, Karaiskou N, Apostolidis A, Kirmizidis G (2001) Mitochondrial DNA sequence variation in Greeks. Hum Biol 73: 855-869.

28. Vernesi C, Di Benedetto G, Caramelli D, Secchieri E, Simoni L, et al. (2001) Genetic characterization of the body attributed to the evangelist Luke. Proc Natl Acad Sci U S A 98: 13460-13463.

29. Helgason A, Sigureth ardottir S, Gulcher JR, Ward R, Stefansson K (2000) mtDNA and the origin of the Icelanders: deciphering signals of recent population history. Am J Hum Genet 66: 999-1016.

30. Ottoni C, Martinez-Labarga C, Vitelli L, Scano G, Fabrini E, et al. (2009) Human mitochondrial DNA variation in Southern Italy. Ann Hum Biol 36: 785-811.

31. Bini C, Ceccardi S, Luiselli D, Ferri G, Pelotti S, et al. (2003) Different informativeness of the three hypervariable mitochondrial DNA regions in the population of Bologna (Italy). Forensic Sci Int 135: 48-52.

32. Achilli A, Olivieri A, Pala M, Metspalu E, Fornarino S, et al. (2007) Mitochondrial DNA variation of modern Tuscans supports the near eastern origin of Etruscans. Am J Hum Genet 80: 759-768.

33. Turchi C, Buscemi L, Previdere C, Grignani P, Brandstatter A, et al. (2008) Italian mitochondrial DNA database: results of a collaborative exercise and proficiency testing. Int J Legal Med 122: 199-204.

34. Morelli L, Grosso MG, Vona G, Varesi L, Torroni A, et al. (2000) Frequency distribution of mitochondrial DNA haplogroups in Corsica and Sardinia. Hum Biol 72: 585-595.

35. Messina F, Scorrano G, Labarga CM, Rolfo MF, Rickards O (2010) Mitochondrial DNA variation in an isolated area of Central Italy. Ann Hum Biol 37: 385-402.

36. Fraumene C, Petretto E, Angius A, Pirastu M (2003) Striking differentiation of sub-populations within a genetically homogeneous isolate (Ogliastra) in Sardinia as revealed by mtDNA analysis. Hum Genet 114: 1-10.

37. Francalacci P, Bertranpetit J, Calafell F, Underhill PA (1996) Sequence diversity of the control region of mitochondrial DNA in Tuscany and its implications for the peopling of Europe. Am J Phys Anthropol 100: 443-460.

38. Comas D, Calafell F, Mateu E, Perez-Lezaun A, Bosch E, et al. (1998) Trading genes along the silk road: mtDNA sequences and the origin of central Asian populations. Am J Hum Genet 63: 1824-1838.

39. DI Rienzo A, Wilson AC (1991) Branching pattern in the evolutionary tree for human mitochondrial DNA. Prot Natl Acad Sci USA 88: 1597-1601.

40. Dupuy BM, Olaisen B (1996) mtDNA sequences in Norwegian Saami and main populations. Adv Forensic Homogen 6: 23-25.

41. Malyarchuk BA, Derenko MV (2001) Mitochondrial DNA variability in Russians and Ukrainians: implication to the origin of the Eastern Slavs. Ann Hum Genet 65: 63-78.

42. Casas MJ, Hagelberg E, Fregel R, Larruga JM, Gonzalez AM (2006) Human mitochondrial DNA diversity in an archaeological site in al-Andalus: genetic impact of migrations from North Africa in medieval Spain. Am J Phys Anthropol 131: 539-551.

43. Pult I, Sajantila A, Simanainen J, Georgiev O, Schaffner W, et al. (1994) Mitochondrial DNA sequences from Switzerland reveal striking homogeneity of European populations. Biol Chem Hoppe Seyler 375: 837-840.

44. Comas D, Calafell F, Mateu E, Perez-Lezaun A, Bertranpetit J (1996) Geographic variation in human mitochondrial DNA control region sequence: the population history of Turkey and its relationship to the European populations. Mol Biol Evol 13: 1067-1077.

45. Di Benedetto G, Erguven A, Stenico M, Castri L, Bertorelle G, et al. (2001) DNA diversity and population admixture in Anatolia. Am J Phys Anthropol 115: 144-156.

46. Quintana-Murci L, Chaix R, Wells RS, Behar DM, Sayar H, et al. (2004) Where west meets east: the complex mtDNA landscape of the southwest and Central Asian corridor. Am J Hum Genet 74: 827-845.

47. Caramelli D, Vernesi C, Sanna S, Sampietro L, Lari M, et al. (2007) Genetic variation in prehistoric Sardinia. Hum Genet 122: 327-336.

48. Sampietro ML, Caramelli D, Lao O, Calafell F, Comas D, et al. (2005) The genetics of the pre-Roman Iberian Peninsula: a mtDNA study of ancient Iberians. Ann Hum Genet 69: 535-548.

49. Vernesi C, Caramelli D, Dupanloup I, Bertorelle G, Lari M, et al. (2004) The Etruscans: a population-genetic study. Am J Hum Genet 74: 694-704.

50. Ghirotto S, Tassi F, Fumagalli E, Colonna V, Sandionigi A, et al. (2013) Origins and evolution of the Etruscans' mtDNA. PLoS One 8: e55519.

51. Guimaraes S, Ghirotto S, Benazzo A, Milani L, Lari M, et al. (2009) Genealogical discontinuities among Etruscan. Medieval. and contemporary Tuscans. Mol Biol Evol 26: 2157-2166.
