# Supplementary material for: Genealogical Relationships between Early Medieval and Modern Inhabitants of Piedmont
Source: PLoS One. 2015 Jan 30;10(1):e0116801. doi: 10.1371/journal.pone.0116801 (PMC4312042; doi:10.1371/journal.pone.0116801)
Supplement: S3 Table — Some distributions, marked by the asterisk, are model-specific. (DOCX) [file pone.0116801.s008.docx]

| Modern effective population size | loguniform (1000,100000) |
| --- | --- |
| Start of the exponential growth * | uniform(56,2000) |
| Lombard effective population size * | uniform (1000,30000) |
| **Separation Time *** | **uniform (56,2000)** |
| Ancient population sizes | uniform (10,1000) |
| Bottleneck Time * | uniform (1,400) |
| Start of the exponential growth before the bottleneck* | uniform (400, 1600) |
| Separation time before the bottleneck* | uniform (400, 1600) |
| Bottleneck population size* | uniform (100, 10000) |
| Bottleneck strength * | uniform (1.1, 10) |
| Mutation rate | uniform ( 0.00030,0.0075) |

**Table S3: Prior distributions of the simulated models. Some distributions, marked by the asterisk, are model-specific.**
