# Supplementary material for: Genealogical Relationships between Early Medieval and Modern Inhabitants of Piedmont
Source: PLoS One. 2015 Jan 30;10(1):e0116801. doi: 10.1371/journal.pone.0116801 (PMC4312042; doi:10.1371/journal.pone.0116801)
Supplement: S4 Table — These values were used in the ABC analysis. (DOCX) [file pone.0116801.s009.docx]

**Table S4. Statistics summarizing intra (A) and inter (B) population genetic diversity. These values were used in the ABC analysis.**

| **A** | | **Lombards** | **Trino Vercellese** | **Postua** | **Val Susa** | **Torino** |
| --- | --- | --- | --- | --- | --- | --- |
| Number of sequences | | 28 | 75 | 88 | 58 | 50 |
| Number of distinct haplotypes sites | | 18 | 47 | 27 | 32 | 44 |
| Segregating sites | | 23 | 53 | 37 | 30 | 48 |
| Mean pairwise difference | | 3.532 | 4.382 | 2.800 | 3.246 | 4.786 |
| Haplotype diversity | | 0.942 | 0.971 | 0.902 | 0.959 | 0.992 |
| **B** | | **Lombards** | **Trino Vercellese** | **Postua** | **Val Susa** | **Torino** |
| Fst | **Lombards** | 0.000 | 0.008 | 0.048 | 0.024 | 0.012 |
| Allele sharing | **Lombards** | 1.000 | 0.228 | 0.056 | 0.222 | 0.167 |
