# Supplementary material for: Genealogical Relationships between Early Medieval and Modern Inhabitants of Piedmont
Source: PLoS One. 2015 Jan 30;10(1):e0116801. doi: 10.1371/journal.pone.0116801 (PMC4312042; doi:10.1371/journal.pone.0116801)
Supplement: S5 Table — (DOCX) [file pone.0116801.s010.docx]

**Table S5. HVR-I motifs of the researchers who had been in contact with the ancient samples.**

| **Archaeologist/Anthropologists/**  **Molecular Anthropologists** | **Mitochondrial profiles** |
| --- | --- |
| Archaeologist | T16126C  C16184T  C16294T  C16296T |
| Anthropologist 1 | T16092C  A16183C  T16189C  C16278T |
| Anthropologist 2 | G16145A  C16176G  C16223T |
| Molecular Anthropologist 1 | T16311C |
| Molecular Anthropologist 2 | C16069T  T16126C  G16145A  T16172C  C16222T  C16261T |
