# Supplementary material for: Genealogical Relationships between Early Medieval and Modern Inhabitants of Piedmont
Source: PLoS One. 2015 Jan 30;10(1):e0116801. doi: 10.1371/journal.pone.0116801 (PMC4312042; doi:10.1371/journal.pone.0116801)
Supplement: S6 Table — Top panel: Acceptance-Rejection 100 simulations, Bottom panel: Logistic Regression 50,000 simulations. (DOCX) [file pone.0116801.s011.docx]

**Table S6. Type I Error. Top panel: Acceptance-Rejection 100 simulations, Bottom panel: Logistic Regression 50,000 simulations.**

| AR  100 |  | MODEL 1-continuity  true | MODEL 2-discontinuity |  |
| --- | --- | --- | --- | --- |
| threshold | **>0.5** |  |  |  |
|  |  | **0.993** | 0.007 | 0 |
|  | **>0.6** |  |  |  |
|  |  | **0.987** | 0.006 | 0.007 |
|  | **>0.7** |  |  |  |
|  |  | **0.981** | 0.005 | 0.014 |
|  | **>0.8** |  |  |  |
|  |  | **0.959** | 0.003 | 0.038 |
|  | **>0.9** |  |  |  |
|  |  | **0.906** | 0.002 | 0.092 |
|  |  | true positives | false positives | not assigned |
|  |  | MODEL 1-continuity | **MODEL 2-discontinuity**  **true** |  |
| threshold | **>0.5** |  |  |  |
|  |  | 0.041 | **0.959** | 0 |
|  | **>0.6** |  |  |  |
|  |  | 0.034 | **0.955** | 0.011 |
|  | **>0.7** |  |  |  |
|  |  | 0.024 | **0.946** | 0.03 |
|  | **>0.8** |  |  |  |
|  |  | 0.018 | **0.937** | 0.045 |
|  | **>0.9** |  |  |  |
|  |  | 0.011 | **0.920** | 0.069 |
|  |  | false positives | true positives | not assigned |

| LR  50,000 |  | MODEL 1-continuity  true | MODEL 2-discontinuity |  |
| --- | --- | --- | --- | --- |
| threshold | **>0.5** |  |  |  |
|  |  | **0.983** | 0.017 | 0 |
|  | **>0.6** |  |  |  |
|  |  | **0.976** | 0.011 | 0.013 |
|  | **>0.7** |  |  |  |
|  |  | **0.97** | 0.007 | 0.023 |
|  | **>0.8** |  |  |  |
|  |  | **0.949** | 0.005 | 0.046 |
|  | **>0.9** |  |  |  |
|  |  | **0.895** | 0.004 | 0.101 |
|  |  | true positives | false positives | not assigned |
|  |  | MODEL 1-continuity | **MODEL 2**-**discontinuity**  **true** |  |
| threshold | **>0.5** |  |  |  |
|  |  | 0.033 | **0.967** | 0 |
|  | **>0.6** |  |  |  |
|  |  | 0.026 | **0.963** | 0.011 |
|  | **>0.7** |  |  |  |
|  |  | 0.021 | **0.958** | 0.021 |
|  | **>0.8** |  |  |  |
|  |  | 0.017 | **0.949** | 0.034 |
|  | **>0.9** |  |  |  |
|  |  | 0.013 | **0.938** | 0.049 |
|  |  | false positives | true positives | not assigned |
